# In Silico-Identified Peptides of Five *Borrelia burgdorferi* Proteins Binding with High Affinity to Human Leukocyte Antigen (HLA) Class II Alleles

**DOI:** 10.3390/biology15070547

**Published:** 2026-03-28

**Authors:** Apostolos P. Georgopoulos, Lisa M. James, Matthew Sanders

**Affiliations:** 1 The HLA and Chronic Diseases Research Groups, Brain Sciences Center, Department of Veterans Affairs Health Care System, Minneapolis, MN 55417, USA; lmjames@umn.edu (L.M.J.); sande568@umn.edu (M.S.); 2 Department of Neuroscience, University of Minnesota Medical School, Minneapolis, MN 55455, USA; 3 Institute for Health Informatics, University of Minnesota Medical School, Minneapolis, MN 55455, USA; 4 Department of Psychiatry, University of Minnesota Medical School, Minneapolis, MN 55455, USA

**Keywords:** Lyme disease, vaccines, *Borrelia burgdorferi*, post-treatment Lyme disease syndrome (PTLDS), human leukocyte antigen (HLA)

## Abstract

To date, Lyme vaccine development has largely overlooked the vaccinee’s human leukocyte antigen (HLA) genetic makeup on which antibody production critically depends. Here, we evaluated in silico the predicted binding affinities of 192 HLA-II alleles with all 15-mer peptide sequences of five *Borrelia burgdorferi* proteins to identify peptides with strong binding affinity, as they would be the best candidates for antibody production in response to vaccination. We found the following: (a) 226 of the 1067 peptides tested (21.2%) were found to bind strongly to HLA-II molecules; (b) decorin-binding protein A had the greatest number of strongly binding peptides; and (c) 69 HLA-II alleles (primarily of the DRB1 gene) bound with strong affinity to peptides from *Borrelia burgdorferi* proteins. Finally, we tested for possible susceptibility to autoimmunity by any one of the 226 peptides above by searching for their occurrence in ~84,000 proteins of the human proteome and found overlap with only two 8-mer peptide sequences (embedded within the 226 15-mer peptides), neither of which was characterized by strong binding to HLA-I, suggesting a reduced likelihood of autoimmunity. These findings emphasize the importance of a personalized vaccine approach based on the vaccinee’s human leukocyte antigen genetic makeup and offer specific vaccine-candidate peptides that are predicted to maximize vaccine effectiveness and safety. The results of this computational study provide novel directions for future development of Lyme vaccines.

## Simple Summary

Development of a vaccine against Lyme disease is ongoing and important in light of the major public health concerns related to increased incidence and geographic spread of the disease by infected ticks and the potential for chronic sequalae due to infection. For all vaccines, key considerations are safety and effectiveness, both of which partially depend on individual variation in human leukocyte antigens which are critically involved in the human immune system response; yet, the immunogenetic makeup of vaccinees is typically not taken into consideration in vaccine development. Here, we determined the predicted ability of common human leukocyte antigens to bind with Lyme-associated proteins, a critical first step in effective antibody production to protect against Lyme disease. We identified several protein peptides that are computationally predicted to bind with strong affinity to specific human leukocyte antigens and do not overlap with proteins of the human proteome, reducing the potential for autoimmunity. We propose that these peptides may potentially be good vaccine candidates, although additional research is necessary to confirm their potential efficacy. We also identified peptides which bind with moderate affinity to various human leukocyte antigens and which could be potential vaccine candidates. These findings emphasize the importance of a personalized vaccine approach based on the vaccinee’s human leukocyte antigen genetic makeup and offer specific vaccine-candidate peptides that are predicted to maximize vaccine effectiveness and safety.

## 1. Introduction

### 1.1. Lyme Disease

Lyme disease is a common vector-borne infectious disease resulting from transmission of *Borrelia burgdorferi* (*B. burgdorferi*) or other related *Borrelia* bacteria from infected ticks [1]. Infection typically begins with local erythema migrans, a characteristic bullseye-shaped rash, at the site of the tick bite, often accompanied by malaise, fatigue, headache, and joint/muscle pain. If left untreated, the infection can spread to other skin sites and organs including the brain, heart, and joints, and may eventually lead to Lyme arthritis and other late-stage manifestations [1,2]. Typically, symptoms resolve following a course of antibiotic therapy; however, some individuals experience persistent and impairing fatigue, musculoskeletal pain, and difficulties with concentration and memory, commonly referred to as post-treatment Lyme disease syndrome (PTLDS) [3]. Persistent *B. burgdorferi* antigen has been identified in the synovial fluid of individuals with PTLDS arthritis [4]. Though endemic to certain areas, the geographic distribution of Lyme disease is expanding [5], with reported Lyme disease activities extending to many countries around the world [6]. An estimated 14.5% of the global population is seroprevalent for Lyme disease [7] and the disease incidence is increasing [8], highlighting the need for preventive strategies. Prevention of Lyme disease has largely focused on personal protective measures to limit exposure to ticks; however, other prophylactic strategies such as the use of antibiotics [9] and vaccines [10,11] have been extensively investigated for both disease prevention and treatment. With regard to the former, early administration of oral antibiotics such as doxycycline within 72 h of tick removal is recommended for prophylaxis to prevent Lyme disease from “high-risk” bites (e.g., at least 36 h attachment of tick from an identified vector species in an endemic area) but is not recommended for routine use after tick bites [12]. Longer courses of antibiotics are recommended for patients with objective evidence of infection such as erythema migrans to prevent dissemination and sequelae [12]; however, approximately 10% of patients do not respond to antibiotic treatment and develop antibiotic-refractory Lyme arthritis [1]. For those with PTLDS, antibiotic treatment is controversial, with evidence suggesting questionable benefits and enhanced adverse events compared to placebo [13,14].

### 1.2. Vaccines for Lyme Disease

Vaccines represent an alternative prevention approach. Strategies aimed at controlling Lyme disease through vaccination of animal reservoirs and development of anti-tick vaccines are promising avenues aimed at preventing Lyme disease by blocking transmission of *Borrelia* to humans [11,15] Alternative vaccine strategies have targeted the spirochete itself, largely involving outer surface protein A (OspA) of *B. burgdoferi*. LYMErix, a Lyme disease vaccine developed in the 1990s and the only Lyme vaccine approved by the Food and Drug Administration to date, was promising with regard to high efficacy, but consumer safety concerns and vaccine hesitancy resulted in it being taken off the market in 2002 [16]. Efforts to develop Lyme vaccines are ongoing with several vaccines in advanced clinical development [17]. Multivalent OspA vaccines have undergone the most clinical testing [17,18,19]; however, other *B. burgdoferi* proteins are also considered vaccine candidates that have shown promise in canine and murine studies [10,11,17]. Identification of immunogenic *B. burgdoferi* antigens is an active area of investigation [20]. Like many other pathogens, *Borrelia* changes the expression of surface antigens in an effort to evade immune system responses aimed at its elimination [2,21]. For example, OspA, the primary target of current vaccine efforts, is expressed during tick colonization, whereas OspC is expressed during early infection followed by expression of variable large protein (VLP) [2]. On the one hand, this antigenic variation thwarts the adaptive immune system responses, permitting persistence and long-term sequelae [22] and, on the other hand, offers multiple *Borrelia* antigens as potential vaccine targets.

### 1.3. Vaccines and Human Leukocyte Antigen (HLA)

Vaccination, whether preventive or therapeutic, is aimed to protect the host against non-self antigens. Preventive vaccines stimulate production of antibodies against given antigens, priming the immune system to neutralize that antigen in the event of *future* exposure, to prevent disease. Therapeutic vaccines harness the immune system to target specific antigens (e.g., viruses, bacteria, cancer antigens) associated with an *existing* disease. In both cases, human leukocyte antigens (HLAs) are instrumental. In case of an infection, HLA molecules bind and present non-self antigens to T cells to stimulate immune response aimed at eliminating non-self antigens. There are two main classes of classical HLA—Class I (HLA-A, HLA-B, and HLA-C genes) and Class II (HLA-DPB1, HLA-DQB1, and HLA-DRB1 genes). Class I molecules are expressed on the surface of all nucleated cells and are involved in presentation of small (8–10 amino acid residues; mostly 9-mer) endogenous antigen peptides to cytotoxic CD8+ T cells to signal cell destruction. Class II molecules are expressed on professional APCs including macrophages, B cells, and DCs, and present larger (15–22 amino acid residues; mostly 15-mer) exogenous antigens to CD4+ helper T cells to stimulate production of antibodies and facilitate immunological memory to enable immune system response to re-infection. HLA composition is genetically determined and includes two alleles from each of the classical Class I genes (A, B, C) and two from each Class II gene (DPB1, DQB1, DRB1) that determine the cell-surface molecules each individual possesses. Some HLA alleles are common across populations [23]; nonetheless, there is tremendous variation across individuals, as reflected in the fact that the HLA region is the most polymorphic in the human genome [24]. Most of the variability across HLA polymorphisms is located in the binding groove, determining the repertoire of antigens that can bind with sufficient affinity to promote an immune response [25,26,27,28]. Structural differences in the binding groove for HLA Class I and Class II determine the number of amino acids of a given peptide that can be accommodated [29]; the specific sequence of the binding groove determines which specific peptides can bind with high affinity, i.e., strong binding, with single amino acid differences altering binding affinity [26]. The importance of HLA–antigen binding is relevant for both natural host adaptive immunity and for vaccine effectiveness. That is, the ability of an individual to mount an effective immune response to an antigen, either through natural exposure or vaccination, is predicated on strong HLA binding to an antigen. Indeed, variation in individual HLA composition is related to vaccine response [30,31,32]. Similarly, individual variation in HLA is associated with susceptibility to, or protection from, various conditions including autoimmune diseases, cancers, and chronic illnesses [33,34,35]. With regard to HLA and *Borrelia* infection, HLA-DRB1 Class II molecules have been implicated in Lyme disease and linked to pro-inflammatory immune dysregulation and autoimmunity, potentially resulting from molecular mimicry of *Borrelia* protein (OspA) with host proteins [36,37]. Here, we investigated in silico the binding affinity of antigens from five proteins that are widely expressed in Borrelia and have been used frequently in vaccine development (particularly OspA) [10] to a large number of HLA-II molecules. We identified peptides with strong predicted binding affinity and searched for their potential presence in 83,607 proteins of the human proteome to reduce likelihood of autoimmunity, a major concern in previously approved Lyme vaccines and an important consideration for future vaccines for Lyme disease.

## 2. Materials and Methods

### 2.1. B. burgdorferi Antigens

We tested 5 protein antigens of *B. burgdorferi* (Table 1). The amino acid (AA) sequences of those proteins were obtained from the UniProt website https://www.uniprot.org/uniprotkb/ (accessed on 5 May 2024) [38] and are shown in Table S1 (Supplementary Materials).

### 2.2. HLA Alleles

We investigated 192 HLA-II alleles (Table S2) [23].

### 2.3. In Silico Determination of Predicted Binding Affinities (PBAs) to B. burgdorferi Antigens

**Figure 1:**
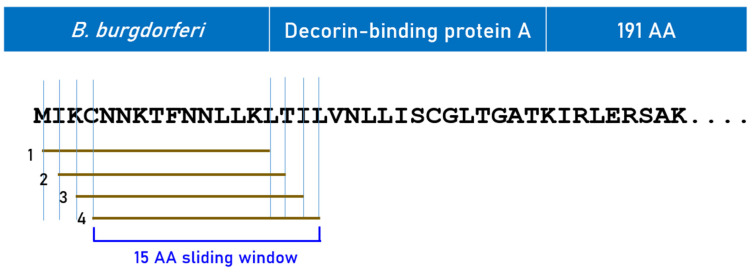
Schematic diagram to illustrate the sliding window approach to estimate in silico binding affinities of HLA alleles to decorin-binding protein A.

Predicted binding affinities were obtained for antigen peptides using the Immune Epitope Database (IEDB) NetMHCpan MHC-II Binding/Elution (ver. 4.1 BA; recommended binding predictor 2023-09) tool [39,40]. More specifically, we used the sliding window approach [41,42,43] to test exhaustively all possible linear 15-mer (HLA-II predictions) peptides of the 5 antigens analyzed (Table 1). The method is illustrated in Figure 1. For each pair of peptide–HLA molecule tested, this tool gave, as an output, the IC_50_ of the predicted binding affinity; *the smaller the IC_50_, the stronger the binding affinity*. IC_50_ values of <50 nM (nanomolar) are regarded as strong, and values of 50 nM ≤ IC_50_ < 500 nM are regarded as moderate [44]. All predicted binding affinities <500 nM were analyzed. Given a protein with an amino acid length of *N* and a peptide length of *k* AA, *N*-*k*+1 IC_50_ values were returned by the prediction tool. The numbers of peptides tested for each antigen are given in Table 1. Finally, the NetMHCpan MHC-I Binding/Elution (ver. 4.1 BA; recommended binding predictor 2023-09) tool [39,40] was used to determine the estimated binding affinity of two octamers (against 142 HLA-I alleles) encountered while testing for autoimmunity.

### 2.4. Protein Comparisons

Target sequences were compared against the human proteome dataset version 24.1, provided by The Human Protein Atlas [45], comprising a total of 83,607 human proteins. Assuming that 15-mer peptides will likely be cleaved to shorter peptides that could trigger autoimmunity by engaging the HLA-I, we also searched for the possible occurrence of shorter peptides (embedded within 15-mers) in the proteins above. For that purpose, we utilized a substring identification method to examine every potential substring of a given length (8–14 amino acids) in the target sequence against the sequences contained in the proteome dataset. We stopped at 8-mers because this is the lower limit of peptide length for strong binding to HLA Class I molecules [46,47]. Given a peptide of *m*-AA length to be tested, a sliding window of the same *m*-AA length was used to identify possible peptides with the same sequence for each one of the 83,607 proteins above. The comparison was performed using the STRPOS function built in PHP (hypertext processor). The function returns the start AA position in the protein sequence where an identical peptide was found. A return of zero means that no peptides were found of the same sequence as the test peptide. No modifications or substitutions were made to the target sequence or proteome sequences.

### 2.5. Statistical Analyses

The IBM-SPSS statistical package (version 30.0.0.0 172) was used for implementing statistical analyses. Standard statistical methods were used; all correlations are Pearson. All *p*-values reported are two-sided, *a* = 0.05.

## 3. Results

### 3.1. Predicted Binding Affinities of B. burgdoferi Antigens and 192 HLA Alleles

Here, we evaluated the predicted binding affinities of five *B. burgdorferi* antigens (Table 1; Table S1, Supplementary Materials) to 192 common HLA-II allele molecules (Table S2) by testing exhaustively in silico 1067 linear 15-mer peptides (epitopes), for a total of 1067 × 192 = 204,464 determinations (Table 1). Overall, we identified 1087/204,464 (0.532%) cases of peptide–allele complexes (pHLA) with predicted strong binding affinities (IC_50_ < 50 nM). Details of the peptide/HLA composition of these complexes are given in Table S3, including their placement in a particular protein and the associated HLA-II allele.

As expected, the number of pHLA complexes with moderate binding was much higher (20,152/204,464 = 9.86%).

### 3.2. B. burgdorferi Antigens

**Figure 2:**
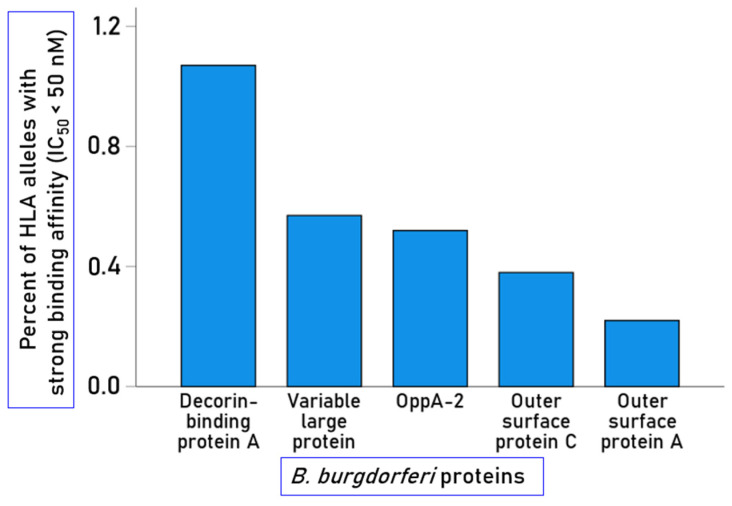
Percent of strongly binding 15-mer peptides for the 5 *B. burgdorferi* proteins tested.

The numbers and percentages of peptides identified as binding with strong affinity per *B. burgdorferi* protein are given in Table 2 and illustrated in Figure 2; the numbers and percentages of peptides binding with moderate affinity are also provided in Table 2. It can be seen that the highest percentage of strong (and moderate) binding was observed for decorin-binding protein A. The number of strong binders in a given protein was not significantly correlated with the AA protein length (r = 0.503, *p* = 0.388).

As expected, the number of peptides with moderate binding (N = 20,152, 9.837%) was much more frequent than that of strong binders (N = 1087, 0.532%). The moderate and strong counts were highly correlated (r = 0.51, *p* = 0.013, N = 5 proteins).

### 3.3. HLA-II Alleles

**Figure 3:**
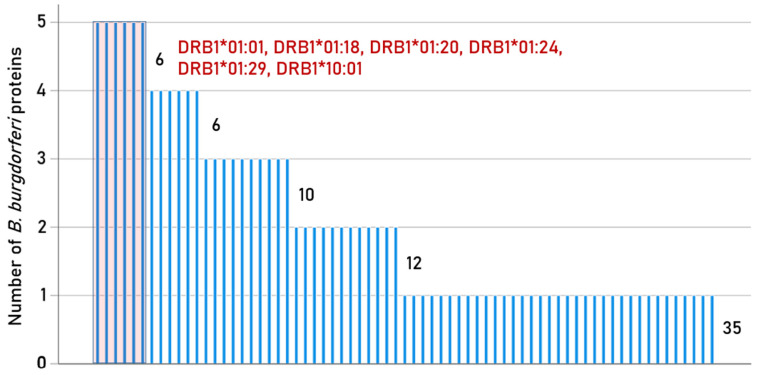
Bar graph illustrating the number of proteins (out of 5 total *B. burgdorferi* proteins) containing peptides that bind strongly to the 69 HLA-II alleles. Sixty-nine alleles bind strongly to at least 1 *B. burgdorferi* protein; six HLA-II alleles are capable of strong binding affinity to all 5 *B. burgdorferi* proteins.

Of the 192 HLA-II alleles tested, strong binding to any *B. burgdorferi* protein was observed in 69 (35.9%) cases (Table 3, Figure 3). Notably, most (66/69, 96%) of these alleles belonged to the DRB1 gene, whereas 3/69 (4%) belonged to the DBP1 gene; no strong binder was found from the DQB1 gene. Summary details of protein-HLA strong binders are given in Table S4. The estimated prevalence of 46 of the alleles in six ethnic populations obtained from donor registries spanning the globe [23] is shown in Table 4 (the prevalence of the remaining alleles was not available). It can be seen that the coverage is substantial in all populations for the 3 DRB1 alleles but very small for the 3 DPB1 alleles.

Moderate binding was observed in 169/192 (88.0%) of alleles tested (Table S7). There were 40 DPB1 alleles with moderate binding (vs. 3 with strong binding), 14 DQB1 alleles (vs. none with strong binding), and 115 DRB1 alleles with moderate binding (vs. 36 with strong binding).

### 3.4. Protein–Allele Combinations

**Figure 4:**
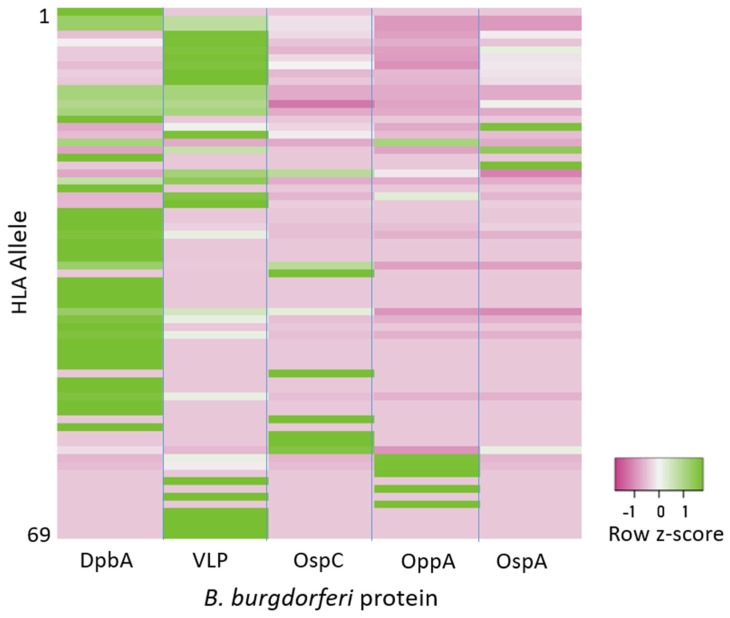
Heatmap of counts of strong binders per protein and allele, normalized (expressed as z-scores) per protein (row). Z-scores are those of data in the rows of Table 5.

The numbers of strongly binding peptides per allele and protein are given in Table 5 and illustrated in Figure 4. It can be seen that there was substantial variation in the occurrence of strong binders among proteins and alleles, with decorin-binding protein A (DpbA) having the highest allele coverage (48/69 = 69.6%) and OspA the lowest (14/69 = 20.3%).

### 3.5. Strongly Binding Peptides and Their Amino Acid Sequences

Of the 1067 peptides tested from the 5 *B. burgdorferi* proteins (Table 1), 226 (21.2%) distinct peptides were found to bind strongly (IC_50_ < 50 nM) to at least one HLA-II allele molecule. The amino acid sequences of these peptides and associated descriptive statistics (number of occurrences, mean and minimum [strongest] estimated binding affinity) are given in Table S5. Each peptide occurred only in one of the five proteins tested, as many times as the number in column N in Table S5.

As expected, the number of distinct, moderately binding peptides was much higher (731/1067 = 68.51%). The AA sequences of those peptides are given in Table S8.

### 3.6. Possible Autoimmunity of Strong Binders

We tested for possible susceptibility to autoimmunity by any one of the 226 peptides above by searching for its occurrence in any of 83,607 human proteins. We did not find any such occurrence. In addition, we searched for the presence of amino acid subsequences (8–14 mers, embedded within the 15-mer peptides), to identify peptides present in human proteins. Indeed, we found two 8-mers shown in color in Table S5 (GKLFESVE, LVKAVKTA); GKLFESVE occurred in protein Q9H2C0 (Uniprot ID; Gigaxonin), whereas LVKAVKTA occurred in protein Q6MZZ7 (Uniprot ID; Calpain-13). We then estimated the binding affinity of these two 8-mers to 142 common HLA-I alleles shown in Table S6. Neither 8-mer peptide had strong affinity to any of the 142 HLA-I allele molecules; the lowest IC_50_ was 6408. Altogether, these results indicate a reduced likelihood of possible autoimmunity by the 226 15-mer linear epitopes evaluated, at least for the HLA-I and HLA-II allele molecules tested.

## 4. Discussion

### 4.1. The Current Lyme Vaccine Landscape

Development of a safe and effective vaccine for Lyme disease is an active area of research that is aimed to stem the increasing incidence and geographic spread of Lyme disease and sequalae. The leading current vaccine candidate, VLA15, is a multivalent OspA-based vaccine for which several Phase I and Phase 2 trials have recently been completed with encouraging initial results [48,49,50,51,52,53]. Nevertheless, there are a good number of other approaches in developing a Lyme vaccine (reviewed in detail in [10,17]), including the targeting of *B. burgdorferi* proteins other than OspA such as VLsE [54], OspC [55], and other proteins [20]. In addition, different technologies are being explored, such as DNA-based [56], m-RNA lipid nanoparticle-based [57], and bioinformatic methods for vaccine design in general [58]. It should be mentioned that none of the studies above have taken into account the HLA genetic makeup of prospective vaccinees in selecting *Borrelia* epitopes, a central point in the present study, as discussed below.

### 4.2. Dependence of Vaccine Effectiveness on the Vaccinee’s HLA

The foundation of the present study rests on the fact that vaccine effectiveness depends highly on the vaccinee’s HLA composition [30,31,32,59,60,61,62]. HLA-II is involved in the production of antibodies against foreign antigens including viruses and bacteria, and in immunological memory, the very basis of preventative vaccines. Production of antibodies, however, is predicated on binding affinity between a given antigen and HLA-II, which varies across individuals. Strong HLA–peptide binding affinity is optimal for antibody production. Since *B. burgdorferi* antigen expression changes across the life cycle [21], several *Borrelia* antigens may be relevant vaccine candidates. Here, we utilized an in silico approach to evaluate the predicted binding affinities of five *B. burgdorferi* antigens to 192 common HLA-II allele molecules. Strong binding affinity was primarily associated with HLA-II DRB1 alleles, six of which (DRB1*01:01, DRB1*01:18, DRB1*01:20, DRB1*01:24, DRB1*01:29 DRB1*10:01) were found to bind strongly to all five *B. burgdorferi* proteins. Thus, individuals possessing any of those six alleles are predicted to have the most robust immune protection against Lyme disease subsequent to exposure to *B. burgdorferi* antigens, either via infection or preventive vaccination. Still, sixty additional HLA-II DRB1 alleles and three DPB1 alleles had strong binding to at least one of the *B. burgdorferi* proteins, also conferring protection against *B. burgdorferi*. Of the five *B. burgdorferi* antigens tested, the highest percentage of strong binders was documented for decorin-binding protein A (DpbA). DpbA is known to provoke an immune response, although its role in the disease course and utility for therapeutics are uncertain due to opposing findings with regard to protection conferred by DpbA antibodies [63]. All of the *B. burgdorferi* protein antigens were, to some extent, predicted to bind strongly to common HLA-II alleles; OspA, the dominant antigen used in vaccine studies [10,48], had the lowest percentage of strong binding with common Class II alleles. Notably, murine studies have shown that mice immunized with the DpbA-OspA combination were protected 100-fold more against *B. burgdorferi* challenge relative to single-antigen vaccines [64]. Those findings, coupled with predictions from the present study, suggest that vaccines combining antigens from different *Borrelia* proteins may maximize protection. Furthermore, the present findings highlight the potential for developing personalized HLA-based vaccines, in which the *B. burgdorferi* antigens with strong binding affinity for a given individual’s HLA composition are administered, along the same lines as nucleic-acid-based vaccines for other infectious diseases and cancers [65].

Obviously, the most effective vaccines are expected to be for peptide–HLA allele molecule (pHLA) complexes of high stability conferred by strong binding affinity (predicted IC_50_ < 50 nM). Such cases were observed for 69/192 (35.9%) of the HLA-II alleles tested (3 PBB1, 66 DRB1). In contrast, as expected, moderate binding affinity (lower stability) of pHLA complexes (50 nM ≤ IC_50_ < 500 nM) was observed in a wider set of HLA-II alleles (169/192 = 88.0%), namely 40 DPB1, 14 DQB1, and 115 DRB1 alleles. From the perspective of personalized HLA-based Lyme vaccine development, sequences with moderate affinity binding could be used for individuals lacking alleles with strong binding. Although such vaccines may not be as effective as those based on strong binders, they would nevertheless offer a degree of protection. It is worth noting that more than one-third of the DRB1 alleles evaluated in this study (43/115) commonly occur in global donor registries regardless of ethnicity [23], highlighting the potential global reach of HLA-based vaccines against Lyme disease.

### 4.3. Reduced Autoimmunity Risk for Strongly Binding Peptides

Given the controversial history of Lyme vaccine efforts [66], a major emphasis in the ongoing development of vaccines for Lyme disease is focused on reducing potential for autoimmune responses and molecular mimicry [67], which was previously proposed to play a role in Lyme arthritis [37]. Here, we tested all 226 unique 15-mer *B. burgdorferi* peptides that bound with strong affinity to HLA-II against 83,607 proteins of the human proteome and did not find any overlap. Furthermore, exhaustive testing of shorter peptides (8–14 mer) revealed only two 8-mer sequences that occurred in the entire human proteome, neither of which was found to bind strongly with HLA-I alleles. These findings suggest that the 226 *B. burgdorferi* peptide sequences identified as potential strong vaccine candidates here may have relatively reduced likelihood of contributing to autoimmunity.

### 4.4. Next Steps for Vaccine Application

This study provides a list of 69 HLA-II alleles which were found in silico to bind with strong affinity (predicted IC_50_ < 50 nM) to at least one of the five *B. burgdorferi* proteins tested using NetMHCpan 4.1, a gold standard in the field [68]. The key innovation of our study lies in the exhaustive testing of all possible linear epitopes of the standard 15-AA length for HLA-II presentation, thus screening for the most promising epitopes to be evaluated further by in vitro, ex vivo, and in vivo methods. In addition, selecting epitopes with strong predicted binding affinities would reduce false positives that would occur more frequently in cases of weak binding affinities.

Since discrepancies may exist between predicted and actual binding affinities, the next step would be to determine in vitro the binding affinities of the identified 1087 pHLA-II complexes in Table S3 using current methods [69,70] and select those pHLA-II complexes with strong binding affinity and stability. Next, these complexes would be evaluated in their capacity to elicit adequate CD4+ engagement and proliferation, and their capacity (alone or with a suitable adjuvant) to elicit the production of neutralizing antibodies and, ultimately, protect vaccinated animals from Lyme disease upon exposure to *Borrelia* infection. This last step would also be crucial in evaluating the safety of the vaccine, adverse reactions, etc., before entering the clinical trial stage. Now, all of this testing, evaluation, and vaccine production would be possible only for pHLA-II complexes with strong binding affinity, and it is at this very first stage that screening the predicted binding affinity in silico of these complexes is instrumental in vaccine design, a screening provided by this study.

## 5. Conclusions

In summary, we propose a novel approach to Lyme vaccination based on host immunogenetics. Each individual carries six HLA-II alleles which determine the cell-surface molecules that can bind and present foreign peptide antigens to helper T cells to stimulate antibody production. We have identified specific *Borrelia* peptide sequences that bind with strong affinity to common HLA-II (primarily HLA-DRB1) alleles (Table S3, Figure 3) and are not found to overlap with human proteins, thereby reducing the likelihood of autoimmunity. Conceivably, off-the-shelf vaccines containing multiple *Borrelia* peptide antigens that bind with strong affinity to a variety of HLA-DR alleles could be developed for testing. Alternatively, vaccines could be highly personalized, containing specific antigens that are predicted to bind with strong (or at least moderate) affinity to a given individual’s HLA-II composition. The development of effective vaccines against Lyme disease, whether personalized to an individual’s HLA composition or off-the-shelf approaches aimed at maximizing population coverage based on strong HLA binding affinity to frequently occurring alleles, will reduce the public health burden associated with Lyme disease [71,72,73]. Computational approaches to epitope identification have been successfully applied to zoonotic and vector-borne pathogens of medical and veterinary importance [74] and hold promise for the future of host-informed vaccine development against Lyme disease.

## Figures and Tables

**Table 1:** *B. burgdorferi* antigens tested. AA = amino acid length. N = the number of 15-mer sequences for a given protein. N tested refers to the total number of HLA-15-mer peptide sequences tested per protein.

	Protein	UNIPROT ID	AA	N (15-mer)	N Tested (×192 Alleles)
1	Decorin-binding protein A	O50917	191	177	33,984
2	OppA-2	Q6RH12	107	93	17,856
3	Outer surface protein A	P0CL66	273	259	49,728
4	Outer surface protein C	Q07337	210	196	37,632
5	Variable large protein	O06878	356	342	65,664
Total			1137	1067	204,864

**Table 2:** Counts (and % of tested) of strongly (IC < 50 nM) and moderately strong (50 nM ≤ IC_50_ < 500 nM) binding peptides per *B. burgdorferi* antigen. N tested reflects total number of HLA-15-mer peptide sequences tested per protein from Table 1.

Protein	UNIPROT ID	N Tested	N (%) Strong (IC_50_ < 50 nM)	N (%) Moderate (50 nM ≤ IC_50_ < 500 nM)
Decorin-binding protein A	O50917	33,984	364 (1.07)	4879 (14.36)
OppA-2	Q6RH12	17,856	93 (0.52)	2484 (13.91)
Outer surface protein A	P0CL66	49,728	109 (0.22)	3151 (6.34)
Outer surface protein C	Q07337	37,632	145 (0.38)	3635 (9.66)
Variable large protein	O06878	65,664	376 (0.57)	6003 (9.14)
Total		204,864	1087 (0.532%)	20,152 (9.837%)

**Table 3:** Counts (N) of Lyme proteins with 15-mer sequences strongly binding (IC_50_ < 50 nM) to the HLA-II allele molecule listed. The ‘x’ in the Common column indicates the availability of ethnicity prevalence of the specific allele in ref. [23].

	Allele	Common	N Proteins
1	DPB1*15:01	x	1
2	DPB1*33:01	x	3
3	DPB1*71:01	x	3
4	DRB1*01:01	x	5
5	DRB1*01:02	x	4
6	DRB1*01:11		4
7	DRB1*01:18		5
8	DRB1*01:20		5
9	DRB1*01:24		5
10	DRB1*01:29		5
11	DRB1*03:01	x	2
12	DRB1*03:04	x	2
13	DRB1*03:11		4
14	DRB1*03:13	x	2
15	DRB1*03:15	x	1
16	DRB1*04:01	x	3
17	DRB1*04:04	x	2
18	DRB1*04:05		2
19	DRB1*04:08	x	3
20	DRB1*04:10	x	1
21	DRB1*04:72		1
22	DRB1*07:01	x	4
23	DRB1*08:04		2
24	DRB1*08:24		1
25	DRB1*09:01	x	2
26	DRB1*10:01	x	5
27	DRB1*11:01	x	1
28	DRB1*11:02	x	1
29	DRB1*11:03	x	3
30	DRB1*11:04	x	3
31	DRB1*11:08	x	1
32	DRB1*11:10	x	1
33	DRB1*11:12	x	1
34	DRB1*11:13	x	3
35	DRB1*11:14	x	1
36	DRB1*11:27	x	1
37	DRB1*11:28	x	1
38	DRB1*11:29	x	1
39	DRB1*11:37		1
40	DRB1*11:42		4
41	DRB1*11:46		3
42	DRB1*11:49		1
43	DRB1*11:58		3
44	DRB1*11:62		1
45	DRB1*11:65		1
46	DRB1*11:74		1
47	DRB1*13:01	x	1
48	DRB1*13:02	x	1
49	DRB1*13:05	x	1
50	DRB1*13:07	x	1
51	DRB1*13:11	x	3
52	DRB1*13:14	x	1
53	DRB1*13:21	x	1
54	DRB1*13:23		1
55	DRB1*13:50		1
56	DRB1*13:96		1
57	DRB1*13:97		1
58	DRB1*14:32		4
59	DRB1*15:01	x	2
60	DRB1*15:02	x	2
61	DRB1*15:03	x	1
62	DRB1*15:06	x	2
63	DRB1*15:07	x	1
64	DRB1*15:15		2
65	DRB1*15:37		1
66	DRB1*16:01	x	1
67	DRB1*16:02	x	2
68	DRB1*16:05	x	1
69	DRB1*16:09		1

**Table 4:** Reported prevalences of Common alleles (Table 3) in 6 ethnic populations. Values are approximate percentages (allele frequency × 200) computed from ref. [23].

Ethnicities	DRB1 Gene (43 Alleles)	DPB1 Gene (3 Alleles)
AFA (African/African American)	70.3%	0.56%
API (Asian/Pacific Islands)	71.1	0.65
EURO (European/European descent)	84.6	0.76
MENA (Middle East/North Coast of Africa)	75.6	0.87
HIS (South or Central America/Hispanic/Latino)	61.5	0.49
NAM (Native American populations)	64.5	0.46

**Table 5:** Numbers of strongly binding peptides per *B. burgdorferi* protein and HLA allele.

	Allele	DpbA	VLP	OspC	OppA	OspA
1	DPB1*15:01	3	0	0	0	0
2	DPB1*33:01	9	7	3	0	0
3	DPB1*71:01	9	7	3	0	0
4	DRB1*01:01	14	50	18	9	21
5	DRB1*01:02	3	11	1	0	1
6	DRB1*01:11	2	12	1	0	5
7	DRB1*01:18	19	51	21	12	23
8	DRB1*01:20	14	46	22	8	20
9	DRB1*01:24	3	23	1	1	5
10	DRB1*01:29	3	26	3	1	5
11	DRB1*03:01	2	2	0	0	0
12	DRB1*03:04	2	2	0	0	0
13	DRB1*03:11	5	5	0	1	3
14	DRB1*03:13	2	2	0	0	0
15	DRB1*03:15	1	0	0	0	0
16	DRB1*04:01	0	2	1	0	6
17	DRB1*04:04	0	5	1	0	0
18	DRB1*04:05	2	0	0	2	0
19	DRB1*04:08	0	4	1	0	6
20	DRB1*04:10	2	0	0	0	0
21	DRB1*04:72	0	0	0	0	4
22	DRB1*07:01	1	7	6	3	0
23	DRB1*08:04	2	3	0	0	0
24	DRB1*08:24	3	0	0	0	0
25	DRB1*09:01	0	10	0	4	0
26	DRB1*10:01	3	35	6	5	6
27	DRB1*11:01	9	0	0	0	0
28	DRB1*11:02	7	0	0	0	0
29	DRB1*11:03	13	1	0	0	1
30	DRB1*11:04	17	6	1	0	0
31	DRB1*11:08	6	0	0	0	0
32	DRB1*11:10	9	0	0	0	0
33	DRB1*11:12	9	0	0	0	0
34	DRB1*11:13	10	2	8	0	0
35	DRB1*11:14	0	0	6	0	0
36	DRB1*11:27	3	0	0	0	0
37	DRB1*11:28	9	0	0	0	0
38	DRB1*11:29	9	0	0	0	0
39	DRB1*11:37	3	0	0	0	0
40	DRB1*11:42	20	13	11	1	0
41	DRB1*11:46	17	6	1	0	0
42	DRB1*11:49	9	0	0	0	0
43	DRB1*11:58	17	6	1	0	0
44	DRB1*11:62	9	0	0	0	0
45	DRB1*11:65	7	0	0	0	0
46	DRB1*11:74	9	0	0	0	0
47	DRB1*13:01	7	0	0	0	0
48	DRB1*13:02	0	0	6	0	0
49	DRB1*13:05	9	0	0	0	0
50	DRB1*13:07	3	0	0	0	0
51	DRB1*13:11	17	6	1	0	0
52	DRB1*13:14	9	0	0	0	0
53	DRB1*13:21	12	0	0	0	0
54	DRB1*13:23	0	0	6	0	0
55	DRB1*13:50	9	0	0	0	0
56	DRB1*13:96	0	0	3	0	0
57	DRB1*13:97	0	0	6	0	0
58	DRB1*14:32	2	1	7	0	3
59	DRB1*15:01	0	3	0	9	0
60	DRB1*15:02	0	1	0	5	0
61	DRB1*15:03	0	0	0	5	0
62	DRB1*15:06	0	3	0	0	0
63	DRB1*15:07	0	0	0	6	0
64	DRB1*15:15	0	1	0	0	0
65	DRB1*15:37	0	0	0	5	0
66	DRB1*16:01	0	4	0	0	0
67	DRB1*16:02	0	8	0	0	0
68	DRB1*16:05	0	2	0	0	0
69	DRB1*16:09	0	3	0	0	0

## Data Availability

All data used were retrieved from freely accessible websites and, as such, are publicly and freely available in ref. [38]: https://www.uniprot.org/uniprotkb (accessed on 5 May 2024); ref. [40]: http://tools.iedb.org/mhci/ (accessed on 18 October 2025); and ref. [45]: https://www.proteinatlas.org/about/download#protein_atlas_data (accessed on 14 December 2025).

## Supplementary Materials

The following supporting information can be downloaded at https://www.mdpi.com/article/10.3390/biology15070547/s1, Table S1. Amino acid sequences of the 5 proteins of B. burgdorferi used. Table S2. The 192 HLA-II alleles used. Table S3. All strong 15-mer strong binders (IC_50_ < 50 nM). Table S4. Cross-tabulation of counts of occurrences of strong binders between alleles and *B. burgdorferi* proteins. N denotes the number of proteins for which an allele showed strong binding. Total denotes the number of strong peptide binders for the associated allele and protein. Table S5. The 226 unique (distinct) peptides with strong binding affinities. N is the number of occurrences. The 8-mers in color denote subsequences that were found in 2/83,607 (0.24%) human proteins tested. See text for details. Table S6. The 142 HLA Class I alleles used to estimate predicted binding affinities of GKLFESVE, LVKAVKTA 8-mers. See text for details. Table S7. Number of 15-mer peptides with moderate predicted binding affinity to *B. burgdorferi* proteins (50 nM ≤ IC_50_ < 500 nM). Table S8. The 731 distinct 15-mer peptides with moderate predicted binding affinity to 5 *B. burgdorferi* antigens tested.
