# Supplementary material for: In Silico-Identified Peptides of Five Borrelia burgdorferi Proteins Binding with High Affinity to Human Leukocyte Antigen (HLA) Class II Alleles
Source: Biology (Basel). 2026 Mar 28;15(7):547. doi: 10.3390/biology15070547 (PMC13072006; doi:10.3390/biology15070547)
Supplement: Supplementary file 1 [file biology-15-00547-s001.zip › biology-4083062-supplementary.pdf]

Table S1. Amino acid sequences of the 5 proteins of *B. burgdorferi* used.

O50917 · DPBA_BORBU	Decorin-binding protein A (Borrelia burgdorferi)	191 AA

MIKCNNKTFNNLLKLTILVNLLISCGLTGATKIRLERSAKDITDEIDAIKKDAALKGVNFDAFKDKKTGS
GVSENPFILEAKVRATTVAEKFVIAIEEEATKLKETGSSGEFSAMYDLMFEVSKPLQKLGIQEMTKTVSD
AAEENPPTTAQGVLEIAKKMREKLQRVHTKNYCTLKKKENSTFTDEKCKNN

Q6RH12 · OppA-2_BORBG	OppA-2 (Borrelia burgdorferi)	107 AA

RAGWIGDYADPLTFLSIFTQGYTQFSSHNYSNPEYNELIKKSDLELDPIKRQDILRQAEEIIIEKDFPIA
PIYIYGNSYLFRNDKWTGWNTNFLERFDLCQLKLKNK

P0CL66 · OSPA_BORBU	Outer surface protein A (Borrelia burgdorferi)	273 AA

MKKYLLGIGLILALIACKQNVSSLDEKNSVSVDLPGEMKVLVSKEKNKDGKYDLIATVDKLELKGTSDKN
NGSGVLEGVKADKSKVKLTISDDLGQTTLEVFKEDGKTLVSKKVTSKDKSSTEEKFNEKGEVSEKIITRA
DGTRLEYTGIKSDGSGKAKEVLKGYVLEGTLTAEKTTLVVKEGTVTLSKNISKSGEVSVELNDTDSSAAT
KKTAAWNSGTSTLTITVNSKKTKDLVFTKENTITVQQYDSNGTKLEGSAVEITKLDEIKNALK

Q07337 · OSPC_BORBU	Outer surface protein C (Borrelia burgdorferi)	210 AA

MKKNTLSAILMTLFLFISCNNSGKDGNTSANSADESVKGPNLTEISKKITDSNAVLLAVKEVEALLSSID
EIAAKAIGKKIHQNNGLDTENNHNGSLLAGAYAISTLIKQKLDGLKNEGLKEKIDAAKKCSETFTNKLKE
KHTDLGKEGVTDADAKEAILKTNGTKTKGAEELGKLFESVEVLSKAAKEMLANSVKELTSPVVAESPKKP

O06878 · Vlse_BORBG	Variable large protein (Borrelia burgdorferi	356 AA

MKKISSAILLTTFFVFINCKSQVADKDDPTNKFYQSVIQLGNGFLDVFTSFGGLVAEAFGFKSDPKKSDV
KTYFTTVAAKLEKTKTDLNSLPKEKSDISSTTGKPDSTGSVGTAVEGAIKEVSELLDKLVKAVKTAEGAS
SGTAAIGEVVADADAAKVADKASVKGIAKGIKEIVEAAGGSEKLKAVAAAKGENNKGAGKLFGKAGAAAH
GDSEAASKAAGAVSAVSGEQILSAIVTAADAAEQDGKKPEEAKNPIAAAIGDKDGGAEFGQDEMKKDDQI
AAAIALRGMAKDGKFAVKDGEKEKAEGAIKGAAESAVRKVLGAITGLIGDAVSSGLRKVGDSVKAASKET
PPALNK

Table S2. The 192 HLA-II alleles used.

	DPB1 gene		DQB1 gene		DRB1 gene				
1	DPB1*01:01	1	DQB1*02:02	1	DRB1*01:01	41	DRB1*09:02	81	DRB1*13:21
2	DPB1*02:01	2	DQB1*03:01	2	DRB1*01:02	42	DRB1*10:01	82	DRB1*13:23
3	DPB1*02:02	3	DQB1*03:02	3	DRB1*01:03	43	DRB1*11:01	83	DRB1*13:26
4	DPB1*03:01	4	DQB1*03:03	4	DRB1*01:11	44	DRB1*11:02	84	DRB1*13:33
5	DPB1*04:01	5	DQB1*03:04	5	DRB1*01:18	45	DRB1*11:03	85	DRB1*13:50
6	DPB1*04:02	6	DQB1*03:05	6	DRB1*01:20	46	DRB1*11:04	86	DRB1*13:61
7	DPB1*05:01	7	DQB1*03:10	7	DRB1*01:24	47	DRB1*11:06	87	DRB1*13:66
8	DPB1*06:01	8	DQB1*03:14	8	DRB1*01:29	48	DRB1*11:07	88	DRB1*13:96
9	DPB1*09:01	9	DQB1*03:17	9	DRB1*03:01	49	DRB1*11:08	89	DRB1*13:97
10	DPB1*10:01	10	DQB1*03:19	10	DRB1*03:02	50	DRB1*11:10	90	DRB1*14:01
11	DPB1*104:01	11	DQB1*03:23	11	DRB1*03:04	51	DRB1*11:11	91	DRB1*14:02
12	DPB1*105:01	12	DQB1*03:25	12	DRB1*03:05	52	DRB1*11:12	92	DRB1*14:03
13	DPB1*11:01	13	DQB1*04:01	13	DRB1*03:11	53	DRB1*11:13	93	DRB1*14:04
14	DPB1*124:01	14	DQB1*04:02	14	DRB1*03:13	54	DRB1*11:14	94	DRB1*14:05
15	DPB1*126:01	15	DQB1*04:03	15	DRB1*03:15	55	DRB1*11:19	95	DRB1*14:06
16	DPB1*13:01	16	DQB1*05:01	16	DRB1*03:41	56	DRB1*11:27	96	DRB1*14:07
17	DPB1*14:01	17	DQB1*05:02	17	DRB1*04:01	57	DRB1*11:28	97	DRB1*14:12
18	DPB1*15:01	18	DQB1*05:03	18	DRB1*04:02	58	DRB1*11:29	98	DRB1*14:23
19	DPB1*16:01	19	DQB1*05:06	19	DRB1*04:03	59	DRB1*11:37	99	DRB1*14:27
20	DPB1*17:01	20	DQB1*05:11	20	DRB1*04:04	60	DRB1*11:42	100	DRB1*14:32
21	DPB1*19:01	21	DQB1*06:01	21	DRB1*04:05	61	DRB1*11:46	101	DRB1*14:38
22	DPB1*20:01	22	DQB1*06:02	22	DRB1*04:06	62	DRB1*11:49	102	DRB1*14:44
23	DPB1*23:01	23	DQB1*06:03	23	DRB1*04:07	63	DRB1*11:54	103	DRB1*14:54
24	DPB1*26:01	24	DQB1*06:04	24	DRB1*04:08	64	DRB1*11:58	104	DRB1*14:68
25	DPB1*28:01	25	DQB1*06:07	25	DRB1*04:10	65	DRB1*11:62	105	DRB1*15:01
26	DPB1*30:01	26	DQB1*06:08	26	DRB1*04:11	66	DRB1*11:65	106	DRB1*15:02
27	DPB1*33:01	27	DQB1*06:09	27	DRB1*04:17	67	DRB1*11:74	107	DRB1*15:03
28	DPB1*34:01	28	DQB1*06:11	28	DRB1*04:44	68	DRB1*11:84	108	DRB1*15:06
29	DPB1*35:01	29	DQB1*06:14	29	DRB1*04:53	69	DRB1*12:01	109	DRB1*15:07
30	DPB1*39:01	30	DQB1*06:15	30	DRB1*04:56	70	DRB1*12:02	110	DRB1*15:15
31	DPB1*40:01	31	DQB1*06:18	31	DRB1*04:72	71	DRB1*12:03	111	DRB1*15:37
32	DPB1*41:01	32	DQB1*06:19	32	DRB1*07:01	72	DRB1*12:16	112	DRB1*16:01
33	DPB1*46:01	33	DQB1*06:22	33	DRB1*08:01	73	DRB1*13:01	113	DRB1*16:02
34	DPB1*47:01	34	DQB1*06:27	34	DRB1*08:02	74	DRB1*13:02	114	DRB1*16:04
35	DPB1*49:01	35	DQB1*06:32	35	DRB1*08:03	75	DRB1*13:03	115	DRB1*16:05
36	DPB1*55:01			36	DRB1*08:04	76	DRB1*13:05	116	DRB1*16:09
37	DPB1*71:01			37	DRB1*08:24	77	DRB1*13:07		
38	DPB1*72:01			38	DRB1*08:30	78	DRB1*13:11		
39	DPB1*81:01			39	DRB1*08:36	79	DRB1*13:12		
40	DPB1*85:01			40	DRB1*09:01	80	DRB1*13:14		
41	DPB1*91:01								

Table S3. All strong 15-mer strong binders (IC_{50} < 50 nM).

Index	Protein	protein ID	Allele	Start	End	Peptide	
1	Decorin-binding protein A	O50917	DPB1*15:01	111	125	EFSAMYDLMFEVSKP	41.54
2	Decorin-binding protein A	O50917	DPB1*15:01	112	126	FSAMYDLMFEVSKPL	44.78
3	Decorin-binding protein A	O50917	DPB1*15:01	110	124	GEFSAMYDLMFEVSK	48.4
4	Decorin-binding protein A	O50917	DPB1*33:01	111	125	EFSAMYDLMFEVSKP	30.36
5	Decorin-binding protein A	O50917	DPB1*33:01	112	126	FSAMYDLMFEVSKPL	30.47
6	Decorin-binding protein A	O50917	DPB1*33:01	110	124	GEFSAMYDLMFEVSK	34.46
7	Decorin-binding protein A	O50917	DPB1*33:01	113	127	SAMYDLMFEVSKPLQ	42.27
8	Decorin-binding protein A	O50917	DPB1*33:01	53	67	AALKGVNFDAFKDKK	43.42
9	Decorin-binding protein A	O50917	DPB1*33:01	82	96	KVRATTVAEKFVIAI	45.48
10	Decorin-binding protein A	O50917	DPB1*33:01	50	64	KKDAALKGVNFDAFK	47.74
11	Decorin-binding protein A	O50917	DPB1*33:01	52	66	DAALKGVNFDAFKDK	47.85
12	Decorin-binding protein A	O50917	DPB1*33:01	83	97	VRATTVAEKFVIAIE	49.54
13	Decorin-binding protein A	O50917	DPB1*71:01	111	125	EFSAMYDLMFEVSKP	30.36
14	Decorin-binding protein A	O50917	DPB1*71:01	112	126	FSAMYDLMFEVSKPL	30.47
15	Decorin-binding protein A	O50917	DPB1*71:01	110	124	GEFSAMYDLMFEVSK	34.46
16	Decorin-binding protein A	O50917	DPB1*71:01	113	127	SAMYDLMFEVSKPLQ	42.27
17	Decorin-binding protein A	O50917	DPB1*71:01	53	67	AALKGVNFDAFKDKK	43.42
18	Decorin-binding protein A	O50917	DPB1*71:01	82	96	KVRATTVAEKFVIAI	45.48
19	Decorin-binding protein A	O50917	DPB1*71:01	50	64	KKDAALKGVNFDAFK	47.74
20	Decorin-binding protein A	O50917	DPB1*71:01	52	66	DAALKGVNFDAFKDK	47.85
21	Decorin-binding protein A	O50917	DPB1*71:01	83	97	VRATTVAEKFVIAIE	49.54
22	Decorin-binding protein A	O50917	DRB1*01:01	115	129	MYDLMFEVSKPLQKL	13.72
23	Decorin-binding protein A	O50917	DRB1*01:01	114	128	AMYDLMFEVSKPLQK	17.07
24	Decorin-binding protein A	O50917	DRB1*01:01	116	130	YDLMFEVSKPLQKLG	18.29
25	Decorin-binding protein A	O50917	DRB1*01:01	113	127	SAMYDLMFEVSKPLQ	29.63
26	Decorin-binding protein A	O50917	DRB1*01:01	117	131	DLMFEVSKPLQKLGI	30.3
27	Decorin-binding protein A	O50917	DRB1*01:01	75	89	NPFILEAKVRATTVA	35.7
28	Decorin-binding protein A	O50917	DRB1*01:01	74	88	ENPFILEAKVRATTV	41.34
29	Decorin-binding protein A	O50917	DRB1*01:01	89	103	AEKFVIAIEEEATKL	41.93
30	Decorin-binding protein A	O50917	DRB1*01:01	76	90	PFILEAKVRATTVAE	45.22
31	Decorin-binding protein A	O50917	DRB1*01:01	20	34	NLLISCGLTGATKIR	46.53
32	Decorin-binding protein A	O50917	DRB1*01:01	19	33	VNLLISCGLTGATKI	48.44
33	Decorin-binding protein A	O50917	DRB1*01:01	90	104	EKFVIAIEEEATKLK	48.48
34	Decorin-binding protein A	O50917	DRB1*01:01	6	20	NKTFNNLLKLTILVN	48.98
35	Decorin-binding protein A	O50917	DRB1*01:01	18	32	LVNLLISCGLTGATK	49.74
36	Decorin-binding protein A	O50917	DRB1*01:02	115	129	MYDLMFEVSKPLQKL	32.54
37	Decorin-binding protein A	O50917	DRB1*01:02	114	128	AMYDLMFEVSKPLQK	42.65
38	Decorin-binding protein A	O50917	DRB1*01:02	116	130	YDLMFEVSKPLQKLG	45.08
39	Decorin-binding protein A	O50917	DRB1*01:11	115	129	MYDLMFEVSKPLQKL	35.56

40	Decorin-binding protein A	O50917	DRB1*01:11	114	128	AMYDLMFEVSKPLQK	49.89
41	Decorin-binding protein A	O50917	DRB1*01:18	115	129	MYDLMFEVSKPLQKL	12.48
42	Decorin-binding protein A	O50917	DRB1*01:18	116	130	YDLMFEVSKPLQKLG	15.78
43	Decorin-binding protein A	O50917	DRB1*01:18	114	128	AMYDLMFEVSKPLQK	15.97
44	Decorin-binding protein A	O50917	DRB1*01:18	117	131	DLMFEVSKPLQKLGI	23.92
45	Decorin-binding protein A	O50917	DRB1*01:18	75	89	NPFILEAKVRATTVA	25.93
46	Decorin-binding protein A	O50917	DRB1*01:18	113	127	SAMYDLMFEVSKPLQ	27.77
47	Decorin-binding protein A	O50917	DRB1*01:18	74	88	ENPFILEAKVRATTV	30.04
48	Decorin-binding protein A	O50917	DRB1*01:18	6	20	NKTFNNLLKLTILVN	32.88
49	Decorin-binding protein A	O50917	DRB1*01:18	76	90	PFILEAKVRATTVAE	33.28
50	Decorin-binding protein A	O50917	DRB1*01:18	7	21	KTFNNLLKLTILVNL	36.25
51	Decorin-binding protein A	O50917	DRB1*01:18	18	32	LVNLLISCGLTGATK	36.82
52	Decorin-binding protein A	O50917	DRB1*01:18	89	103	AEKFVIAIEEEATKL	37.81
53	Decorin-binding protein A	O50917	DRB1*01:18	19	33	VNLLISCGLTGATKI	39.79
54	Decorin-binding protein A	O50917	DRB1*01:18	5	19	NNKTFNNLLKLTILV	40.77
55	Decorin-binding protein A	O50917	DRB1*01:18	90	104	EKFVIAIEEEATKLK	42.55
56	Decorin-binding protein A	O50917	DRB1*01:18	73	87	SENPFILEAKVRATT	44.81
57	Decorin-binding protein A	O50917	DRB1*01:18	118	132	LMFEVSKPLQKLGIQ	45.37
58	Decorin-binding protein A	O50917	DRB1*01:18	20	34	NLLISCGLTGATKIR	45.44
59	Decorin-binding protein A	O50917	DRB1*01:18	4	18	CNNKTFNNLLKLTIL	48.65
60	Decorin-binding protein A	O50917	DRB1*01:20	115	129	MYDLMFEVSKPLQKL	10.97
61	Decorin-binding protein A	O50917	DRB1*01:20	114	128	AMYDLMFEVSKPLQK	13.37
62	Decorin-binding protein A	O50917	DRB1*01:20	116	130	YDLMFEVSKPLQKLG	14.5
63	Decorin-binding protein A	O50917	DRB1*01:20	113	127	SAMYDLMFEVSKPLQ	23.28
64	Decorin-binding protein A	O50917	DRB1*01:20	75	89	NPFILEAKVRATTVA	24.8
65	Decorin-binding protein A	O50917	DRB1*01:20	117	131	DLMFEVSKPLQKLGI	27.7
66	Decorin-binding protein A	O50917	DRB1*01:20	74	88	ENPFILEAKVRATTV	29.68
67	Decorin-binding protein A	O50917	DRB1*01:20	76	90	PFILEAKVRATTVAE	30.07
68	Decorin-binding protein A	O50917	DRB1*01:20	19	33	VNLLISCGLTGATKI	34.01
69	Decorin-binding protein A	O50917	DRB1*01:20	18	32	LVNLLISCGLTGATK	34.31
70	Decorin-binding protein A	O50917	DRB1*01:20	20	34	NLLISCGLTGATKIR	37.89
71	Decorin-binding protein A	O50917	DRB1*01:20	73	87	SENPFILEAKVRATT	44.07
72	Decorin-binding protein A	O50917	DRB1*01:20	21	35	LLISCGLTGATKIRL	44.86
73	Decorin-binding protein A	O50917	DRB1*01:20	77	91	FILEAKVRATTVAEK	46.35
74	Decorin-binding protein A	O50917	DRB1*01:24	115	129	MYDLMFEVSKPLQKL	34.17
75	Decorin-binding protein A	O50917	DRB1*01:24	114	128	AMYDLMFEVSKPLQK	48.93
76	Decorin-binding protein A	O50917	DRB1*01:24	116	130	YDLMFEVSKPLQKLG	49.1
77	Decorin-binding protein A	O50917	DRB1*01:29	115	129	MYDLMFEVSKPLQKL	35.39
78	Decorin-binding protein A	O50917	DRB1*01:29	116	130	YDLMFEVSKPLQKLG	48.44
79	Decorin-binding protein A	O50917	DRB1*01:29	114	128	AMYDLMFEVSKPLQK	48.98
80	Decorin-binding protein A	O50917	DRB1*03:01	46	60	IDAIKKDAALKGVNF	35.89
81	Decorin-binding protein A	O50917	DRB1*03:01	45	59	EIDAIKKDAALKGVN	47.46
82	Decorin-binding protein A	O50917	DRB1*03:04	46	60	IDAIKKDAALKGVNF	35.89
83	Decorin-binding protein A	O50917	DRB1*03:04	45	59	EIDAIKKDAALKGVN	47.46

84	Decorin-binding protein A	O50917	DRB1*03:11	46	60	IDAIKKDAALKGVNF	22.69
85	Decorin-binding protein A	O50917	DRB1*03:11	45	59	EIDAIKKDAALKGVN	26.67
86	Decorin-binding protein A	O50917	DRB1*03:11	44	58	DEIDAIKKDAALKGV	30.12
87	Decorin-binding protein A	O50917	DRB1*03:11	47	61	DAIKKDAALKGVNFD	31.87
88	Decorin-binding protein A	O50917	DRB1*03:11	48	62	AIKKDAALKGVNFDA	48.57
89	Decorin-binding protein A	O50917	DRB1*03:13	46	60	IDAIKKDAALKGVNF	35.89
90	Decorin-binding protein A	O50917	DRB1*03:13	45	59	EIDAIKKDAALKGVN	47.46
91	Decorin-binding protein A	O50917	DRB1*03:15	46	60	IDAIKKDAALKGVNF	48.49
92	Decorin-binding protein A	O50917	DRB1*04:05	29	43	GATKIRLERSAKDIT	41.04
93	Decorin-binding protein A	O50917	DRB1*04:05	28	42	TGATKIRLERSAKDI	41.85
94	Decorin-binding protein A	O50917	DRB1*04:10	29	43	GATKIRLERSAKDIT	38.91
95	Decorin-binding protein A	O50917	DRB1*04:10	28	42	TGATKIRLERSAKDI	39.61
96	Decorin-binding protein A	O50917	DRB1*07:01	21	35	LLISCGLTGATKIRL	45.05
97	Decorin-binding protein A	O50917	DRB1*08:04	75	89	NPFILEAKVRATTVA	42.28
98	Decorin-binding protein A	O50917	DRB1*08:04	74	88	ENPFILEAKVRATTV	47.9
99	Decorin-binding protein A	O50917	DRB1*08:24	74	88	ENPFILEAKVRATTV	42.84
100	Decorin-binding protein A	O50917	DRB1*08:24	75	89	NPFILEAKVRATTVA	49.68
101	Decorin-binding protein A	O50917	DRB1*08:24	73	87	SENPFILEAKVRATT	49.83
102	Decorin-binding protein A	O50917	DRB1*10:01	89	103	AEKFVIAIEEEATKL	34.85
103	Decorin-binding protein A	O50917	DRB1*10:01	90	104	EKFVIAIEEEATKLK	38.82
104	Decorin-binding protein A	O50917	DRB1*10:01	115	129	MYDLMFEVSKPLQKL	43.97
105	Decorin-binding protein A	O50917	DRB1*11:01	74	88	ENPFILEAKVRATTV	18.15
106	Decorin-binding protein A	O50917	DRB1*11:01	75	89	NPFILEAKVRATTVA	18.37
107	Decorin-binding protein A	O50917	DRB1*11:01	73	87	SENPFILEAKVRATT	21.33
108	Decorin-binding protein A	O50917	DRB1*11:01	72	86	VSENPFILEAKVRAT	24.51
109	Decorin-binding protein A	O50917	DRB1*11:01	76	90	PFILEAKVRATTVAE	29.02
110	Decorin-binding protein A	O50917	DRB1*11:01	6	20	NKTFNNLLKLTILVN	35.05
111	Decorin-binding protein A	O50917	DRB1*11:01	5	19	NNKTFNNLLKLTILV	42.93
112	Decorin-binding protein A	O50917	DRB1*11:01	4	18	CNNKTFNNLLKLTIL	43.3
113	Decorin-binding protein A	O50917	DRB1*11:01	71	85	GVSENPFILEAKVRA	46.31
114	Decorin-binding protein A	O50917	DRB1*11:02	151	165	QGVLEIAKKMREKLQ	32.21
115	Decorin-binding protein A	O50917	DRB1*11:02	152	166	GVLEIAKKMREKLQR	32.82
116	Decorin-binding protein A	O50917	DRB1*11:02	150	164	AQGVLEIAKKMREKL	35.62
117	Decorin-binding protein A	O50917	DRB1*11:02	153	167	VLEIAKKMREKLQRV	35.65
118	Decorin-binding protein A	O50917	DRB1*11:02	24	38	SCGLTGATKIRLERS	43.4
119	Decorin-binding protein A	O50917	DRB1*11:02	75	89	NPFILEAKVRATTVA	44.5
120	Decorin-binding protein A	O50917	DRB1*11:02	154	168	LEIAKKMREKLQRVH	48.9
121	Decorin-binding protein A	O50917	DRB1*11:03	151	165	QGVLEIAKKMREKLQ	18.96
122	Decorin-binding protein A	O50917	DRB1*11:03	152	166	GVLEIAKKMREKLQR	19.97
123	Decorin-binding protein A	O50917	DRB1*11:03	150	164	AQGVLEIAKKMREKL	21.16
124	Decorin-binding protein A	O50917	DRB1*11:03	153	167	VLEIAKKMREKLQRV	22.79
125	Decorin-binding protein A	O50917	DRB1*11:03	154	168	LEIAKKMREKLQRVH	31.15
126	Decorin-binding protein A	O50917	DRB1*11:03	75	89	NPFILEAKVRATTVA	36.04
127	Decorin-binding protein A	O50917	DRB1*11:03	149	163	TAQGVLEIAKKMREK	37.81

128	Decorin-binding protein A	O50917	DRB1*11:03	24	38	SCGLTGATKIRLERS	39.1
129	Decorin-binding protein A	O50917	DRB1*11:03	74	88	ENPFILEAKVRATTV	39.66
130	Decorin-binding protein A	O50917	DRB1*11:03	76	90	PFILEAKVRATTVAE	45.22
131	Decorin-binding protein A	O50917	DRB1*11:03	155	169	EIAKKMREKLQRVHT	45.34
132	Decorin-binding protein A	O50917	DRB1*11:03	23	37	ISCGLTGATKIRLER	45.67
133	Decorin-binding protein A	O50917	DRB1*11:03	25	39	CGLTGATKIRLERSA	48.01
134	Decorin-binding protein A	O50917	DRB1*11:04	151	165	QGVLEIAKKMREKLQ	18.32
135	Decorin-binding protein A	O50917	DRB1*11:04	150	164	AQGVLEIAKKMREKL	18.73
136	Decorin-binding protein A	O50917	DRB1*11:04	152	166	GVLEIAKKMREKLQR	19.2
137	Decorin-binding protein A	O50917	DRB1*11:04	75	89	NPFILEAKVRATTVA	22.38
138	Decorin-binding protein A	O50917	DRB1*11:04	74	88	ENPFILEAKVRATTV	23.48
139	Decorin-binding protein A	O50917	DRB1*11:04	153	167	VLEIAKKMREKLQRV	25.93
140	Decorin-binding protein A	O50917	DRB1*11:04	149	163	TAQGVLEIAKKMREK	30.71
141	Decorin-binding protein A	O50917	DRB1*11:04	128	142	KLGIQEMTKTVSDAA	31.36
142	Decorin-binding protein A	O50917	DRB1*11:04	76	90	PFILEAKVRATTVAE	31.53
143	Decorin-binding protein A	O50917	DRB1*11:04	73	87	SENPFILEAKVRATT	33.27
144	Decorin-binding protein A	O50917	DRB1*11:04	127	141	QKLGIQEMTKTVSDA	36.55
145	Decorin-binding protein A	O50917	DRB1*11:04	116	130	YDLMFEVSKPLQKLG	43.63
146	Decorin-binding protein A	O50917	DRB1*11:04	115	129	MYDLMFEVSKPLQKL	43.73
147	Decorin-binding protein A	O50917	DRB1*11:04	72	86	VSENPFILEAKVRAT	46.47
148	Decorin-binding protein A	O50917	DRB1*11:04	129	143	LGIQEMTKTVSDAAE	48.01
149	Decorin-binding protein A	O50917	DRB1*11:04	126	140	LQKLGIQEMTKTVSD	49.23
150	Decorin-binding protein A	O50917	DRB1*11:04	24	38	SCGLTGATKIRLERS	49.97
151	Decorin-binding protein A	O50917	DRB1*11:08	74	88	ENPFILEAKVRATTV	26.24
152	Decorin-binding protein A	O50917	DRB1*11:08	75	89	NPFILEAKVRATTVA	28.66
153	Decorin-binding protein A	O50917	DRB1*11:08	73	87	SENPFILEAKVRATT	31.27
154	Decorin-binding protein A	O50917	DRB1*11:08	72	86	VSENPFILEAKVRAT	35.68
155	Decorin-binding protein A	O50917	DRB1*11:08	76	90	PFILEAKVRATTVAE	45.31
156	Decorin-binding protein A	O50917	DRB1*11:08	6	20	NKTFNNLLKLTILVN	47.88
157	Decorin-binding protein A	O50917	DRB1*11:10	74	88	ENPFILEAKVRATTV	18.15
158	Decorin-binding protein A	O50917	DRB1*11:10	75	89	NPFILEAKVRATTVA	18.37
159	Decorin-binding protein A	O50917	DRB1*11:10	73	87	SENPFILEAKVRATT	21.33
160	Decorin-binding protein A	O50917	DRB1*11:10	72	86	VSENPFILEAKVRAT	24.51
161	Decorin-binding protein A	O50917	DRB1*11:10	76	90	PFILEAKVRATTVAE	29.02
162	Decorin-binding protein A	O50917	DRB1*11:10	6	20	NKTFNNLLKLTILVN	35.05
163	Decorin-binding protein A	O50917	DRB1*11:10	5	19	NNKTFNNLLKLTILV	42.93
164	Decorin-binding protein A	O50917	DRB1*11:10	4	18	CNNKTFNNLLKLTIL	43.3
165	Decorin-binding protein A	O50917	DRB1*11:10	71	85	GVSENPFILEAKVRA	46.31
166	Decorin-binding protein A	O50917	DRB1*11:12	74	88	ENPFILEAKVRATTV	18.15
167	Decorin-binding protein A	O50917	DRB1*11:12	75	89	NPFILEAKVRATTVA	18.37
168	Decorin-binding protein A	O50917	DRB1*11:12	73	87	SENPFILEAKVRATT	21.33
169	Decorin-binding protein A	O50917	DRB1*11:12	72	86	VSENPFILEAKVRAT	24.51
170	Decorin-binding protein A	O50917	DRB1*11:12	76	90	PFILEAKVRATTVAE	29.02
171	Decorin-binding protein A	O50917	DRB1*11:12	6	20	NKTFNNLLKLTILVN	35.05

172	Decorin-binding protein A	O50917	DRB1*11:12	5	19	NNKTFNNLLKLTILV	42.93
173	Decorin-binding protein A	O50917	DRB1*11:12	4	18	CNNKTFNNLLKLTIL	43.3
174	Decorin-binding protein A	O50917	DRB1*11:12	71	85	GVSENPFILEAKVRA	46.31
175	Decorin-binding protein A	O50917	DRB1*11:13	151	165	QGVLEIAKKMREKLQ	26.81
176	Decorin-binding protein A	O50917	DRB1*11:13	150	164	AQGVLEIAKKMREKL	28
177	Decorin-binding protein A	O50917	DRB1*11:13	152	166	GVLEIAKKMREKLQR	31.24
178	Decorin-binding protein A	O50917	DRB1*11:13	24	38	SCGLTGATKIRLERS	36.72
179	Decorin-binding protein A	O50917	DRB1*11:13	23	37	ISCGLTGATKIRLER	39.69
180	Decorin-binding protein A	O50917	DRB1*11:13	153	167	VLEIAKKMREKLQRV	39.91
181	Decorin-binding protein A	O50917	DRB1*11:13	75	89	NPFILEAKVRATTVA	40.44
182	Decorin-binding protein A	O50917	DRB1*11:13	74	88	ENPFILEAKVRATTV	40.93
183	Decorin-binding protein A	O50917	DRB1*11:13	149	163	TAQGVLEIAKKMREK	43.65
184	Decorin-binding protein A	O50917	DRB1*11:13	128	142	KLGIQEMTKTVSDAA	49.15
185	Decorin-binding protein A	O50917	DRB1*11:27	74	88	ENPFILEAKVRATTV	36.08
186	Decorin-binding protein A	O50917	DRB1*11:27	75	89	NPFILEAKVRATTVA	37.51
187	Decorin-binding protein A	O50917	DRB1*11:27	73	87	SENPFILEAKVRATT	44.07
188	Decorin-binding protein A	O50917	DRB1*11:28	74	88	ENPFILEAKVRATTV	18.15
189	Decorin-binding protein A	O50917	DRB1*11:28	75	89	NPFILEAKVRATTVA	18.37
190	Decorin-binding protein A	O50917	DRB1*11:28	73	87	SENPFILEAKVRATT	21.33
191	Decorin-binding protein A	O50917	DRB1*11:28	72	86	VSENPFILEAKVRAT	24.51
192	Decorin-binding protein A	O50917	DRB1*11:28	76	90	PFILEAKVRATTVAE	29.02
193	Decorin-binding protein A	O50917	DRB1*11:28	6	20	NKTFNNLLKLTILVN	35.05
194	Decorin-binding protein A	O50917	DRB1*11:28	5	19	NNKTFNNLLKLTILV	42.93
195	Decorin-binding protein A	O50917	DRB1*11:28	4	18	CNNKTFNNLLKLTIL	43.3
196	Decorin-binding protein A	O50917	DRB1*11:28	71	85	GVSENPFILEAKVRA	46.31
197	Decorin-binding protein A	O50917	DRB1*11:29	74	88	ENPFILEAKVRATTV	18.15
198	Decorin-binding protein A	O50917	DRB1*11:29	75	89	NPFILEAKVRATTVA	18.37
199	Decorin-binding protein A	O50917	DRB1*11:29	73	87	SENPFILEAKVRATT	21.33
200	Decorin-binding protein A	O50917	DRB1*11:29	72	86	VSENPFILEAKVRAT	24.51
201	Decorin-binding protein A	O50917	DRB1*11:29	76	90	PFILEAKVRATTVAE	29.02
202	Decorin-binding protein A	O50917	DRB1*11:29	6	20	NKTFNNLLKLTILVN	35.05
203	Decorin-binding protein A	O50917	DRB1*11:29	5	19	NNKTFNNLLKLTILV	42.93
204	Decorin-binding protein A	O50917	DRB1*11:29	4	18	CNNKTFNNLLKLTIL	43.3
205	Decorin-binding protein A	O50917	DRB1*11:29	71	85	GVSENPFILEAKVRA	46.31
206	Decorin-binding protein A	O50917	DRB1*11:37	74	88	ENPFILEAKVRATTV	35.7
207	Decorin-binding protein A	O50917	DRB1*11:37	75	89	NPFILEAKVRATTVA	36.68
208	Decorin-binding protein A	O50917	DRB1*11:37	73	87	SENPFILEAKVRATT	43.13
209	Decorin-binding protein A	O50917	DRB1*11:42	150	164	AQGVLEIAKKMREKL	16.33
210	Decorin-binding protein A	O50917	DRB1*11:42	151	165	QGVLEIAKKMREKLQ	16.37
211	Decorin-binding protein A	O50917	DRB1*11:42	152	166	GVLEIAKKMREKLQR	18.76
212	Decorin-binding protein A	O50917	DRB1*11:42	75	89	NPFILEAKVRATTVA	22.84
213	Decorin-binding protein A	O50917	DRB1*11:42	74	88	ENPFILEAKVRATTV	22.88
214	Decorin-binding protein A	O50917	DRB1*11:42	149	163	TAQGVLEIAKKMREK	23.66
215	Decorin-binding protein A	O50917	DRB1*11:42	153	167	VLEIAKKMREKLQRV	24.42

216	Decorin-binding protein A	O50917	DRB1*11:42	128	142	KLGIQEMTKTVSDAA	28.43
217	Decorin-binding protein A	O50917	DRB1*11:42	24	38	SCGLTGATKIRLERS	29.86
218	Decorin-binding protein A	O50917	DRB1*11:42	73	87	SENPFILEAKVRATT	30.39
219	Decorin-binding protein A	O50917	DRB1*11:42	76	90	PFILEAKVRATTVAE	31.24
220	Decorin-binding protein A	O50917	DRB1*11:42	127	141	QKLGIQEMTKTVSDA	32.91
221	Decorin-binding protein A	O50917	DRB1*11:42	23	37	ISCGLTGATKIRLER	34.8
222	Decorin-binding protein A	O50917	DRB1*11:42	115	129	MYDLMFEVSKPLQKL	37.05
223	Decorin-binding protein A	O50917	DRB1*11:42	116	130	YDLMFEVSKPLQKLG	37.19
224	Decorin-binding protein A	O50917	DRB1*11:42	148	162	TTAQGVLEIAKKMRE	37.64
225	Decorin-binding protein A	O50917	DRB1*11:42	25	39	CGLTGATKIRLERSA	39.63
226	Decorin-binding protein A	O50917	DRB1*11:42	129	143	LGIQEMTKTVSDAAE	40.72
227	Decorin-binding protein A	O50917	DRB1*11:42	72	86	VSENPFILEAKVRAT	42.54
228	Decorin-binding protein A	O50917	DRB1*11:42	126	140	LQKLGIQEMTKTVSD	43.82
229	Decorin-binding protein A	O50917	DRB1*11:46	151	165	QGVLEIAKKMREKLQ	18.32
230	Decorin-binding protein A	O50917	DRB1*11:46	150	164	AQGVLEIAKKMREKL	18.73
231	Decorin-binding protein A	O50917	DRB1*11:46	152	166	GVLEIAKKMREKLQR	19.2
232	Decorin-binding protein A	O50917	DRB1*11:46	75	89	NPFILEAKVRATTVA	22.38
233	Decorin-binding protein A	O50917	DRB1*11:46	74	88	ENPFILEAKVRATTV	23.48
234	Decorin-binding protein A	O50917	DRB1*11:46	153	167	VLEIAKKMREKLQRV	25.93
235	Decorin-binding protein A	O50917	DRB1*11:46	149	163	TAQGVLEIAKKMREK	30.71
236	Decorin-binding protein A	O50917	DRB1*11:46	128	142	KLGIQEMTKTVSDAA	31.36
237	Decorin-binding protein A	O50917	DRB1*11:46	76	90	PFILEAKVRATTVAE	31.53
238	Decorin-binding protein A	O50917	DRB1*11:46	73	87	SENPFILEAKVRATT	33.27
239	Decorin-binding protein A	O50917	DRB1*11:46	127	141	QKLGIQEMTKTVSDA	36.55
240	Decorin-binding protein A	O50917	DRB1*11:46	116	130	YDLMFEVSKPLQKLG	43.63
241	Decorin-binding protein A	O50917	DRB1*11:46	115	129	MYDLMFEVSKPLQKL	43.73
242	Decorin-binding protein A	O50917	DRB1*11:46	72	86	VSENPFILEAKVRAT	46.47
243	Decorin-binding protein A	O50917	DRB1*11:46	129	143	LGIQEMTKTVSDAAE	48.01
244	Decorin-binding protein A	O50917	DRB1*11:46	126	140	LQKLGIQEMTKTVSD	49.23
245	Decorin-binding protein A	O50917	DRB1*11:46	24	38	SCGLTGATKIRLERS	49.97
246	Decorin-binding protein A	O50917	DRB1*11:49	74	88	ENPFILEAKVRATTV	18.15
247	Decorin-binding protein A	O50917	DRB1*11:49	75	89	NPFILEAKVRATTVA	18.37
248	Decorin-binding protein A	O50917	DRB1*11:49	73	87	SENPFILEAKVRATT	21.33
249	Decorin-binding protein A	O50917	DRB1*11:49	72	86	VSENPFILEAKVRAT	24.51
250	Decorin-binding protein A	O50917	DRB1*11:49	76	90	PFILEAKVRATTVAE	29.02
251	Decorin-binding protein A	O50917	DRB1*11:49	6	20	NKTFNNLLKLTILVN	35.05
252	Decorin-binding protein A	O50917	DRB1*11:49	5	19	NNKTFNNLLKLTILV	42.93
253	Decorin-binding protein A	O50917	DRB1*11:49	4	18	CNNKTFNNLLKLTIL	43.3
254	Decorin-binding protein A	O50917	DRB1*11:49	71	85	GVSENPFILEAKVRA	46.31
255	Decorin-binding protein A	O50917	DRB1*11:58	151	165	QGVLEIAKKMREKLQ	18.32
256	Decorin-binding protein A	O50917	DRB1*11:58	150	164	AQGVLEIAKKMREKL	18.73
257	Decorin-binding protein A	O50917	DRB1*11:58	152	166	GVLEIAKKMREKLQR	19.2
258	Decorin-binding protein A	O50917	DRB1*11:58	75	89	NPFILEAKVRATTVA	22.38
259	Decorin-binding protein A	O50917	DRB1*11:58	74	88	ENPFILEAKVRATTV	23.48

260	Decorin-binding protein A	O50917	DRB1*11:58	153	167	VLEIAKKMREKLQRV	25.93
261	Decorin-binding protein A	O50917	DRB1*11:58	149	163	TAQGVLEIAKKMREK	30.71
262	Decorin-binding protein A	O50917	DRB1*11:58	128	142	KLGIQEMTKTVSDAA	31.36
263	Decorin-binding protein A	O50917	DRB1*11:58	76	90	PFILEAKVRATTVAE	31.53
264	Decorin-binding protein A	O50917	DRB1*11:58	73	87	SENPFILEAKVRATT	33.27
265	Decorin-binding protein A	O50917	DRB1*11:58	127	141	QKLGIQEMTKTVSDA	36.55
266	Decorin-binding protein A	O50917	DRB1*11:58	116	130	YDLMFEVSKPLQKLG	43.63
267	Decorin-binding protein A	O50917	DRB1*11:58	115	129	MYDLMFEVSKPLQKL	43.73
268	Decorin-binding protein A	O50917	DRB1*11:58	72	86	VSENPFILEAKVRAT	46.47
269	Decorin-binding protein A	O50917	DRB1*11:58	129	143	LGIQEMTKTVSDAAE	48.01
270	Decorin-binding protein A	O50917	DRB1*11:58	126	140	LQKLGIQEMTKTVSD	49.23
271	Decorin-binding protein A	O50917	DRB1*11:58	24	38	SCGLTGATKIRLERS	49.97
272	Decorin-binding protein A	O50917	DRB1*11:62	74	88	ENPFILEAKVRATTV	18.15
273	Decorin-binding protein A	O50917	DRB1*11:62	75	89	NPFILEAKVRATTVA	18.37
274	Decorin-binding protein A	O50917	DRB1*11:62	73	87	SENPFILEAKVRATT	21.33
275	Decorin-binding protein A	O50917	DRB1*11:62	72	86	VSENPFILEAKVRAT	24.51
276	Decorin-binding protein A	O50917	DRB1*11:62	76	90	PFILEAKVRATTVAE	29.02
277	Decorin-binding protein A	O50917	DRB1*11:62	6	20	NKTFNNLLKLTILVN	35.05
278	Decorin-binding protein A	O50917	DRB1*11:62	5	19	NNKTFNNLLKLTILV	42.93
279	Decorin-binding protein A	O50917	DRB1*11:62	4	18	CNNKTFNNLLKLTIL	43.3
280	Decorin-binding protein A	O50917	DRB1*11:62	71	85	GVSENPFILEAKVRA	46.31
281	Decorin-binding protein A	O50917	DRB1*11:65	151	165	QGVLEIAKKMREKLQ	32.21
282	Decorin-binding protein A	O50917	DRB1*11:65	152	166	GVLEIAKKMREKLQR	32.82
283	Decorin-binding protein A	O50917	DRB1*11:65	150	164	AQGVLEIAKKMREKL	35.62
284	Decorin-binding protein A	O50917	DRB1*11:65	153	167	VLEIAKKMREKLQRV	35.65
285	Decorin-binding protein A	O50917	DRB1*11:65	24	38	SCGLTGATKIRLERS	43.4
286	Decorin-binding protein A	O50917	DRB1*11:65	75	89	NPFILEAKVRATTVA	44.5
287	Decorin-binding protein A	O50917	DRB1*11:65	154	168	LEIAKKMREKLQRVH	48.9
288	Decorin-binding protein A	O50917	DRB1*11:74	74	88	ENPFILEAKVRATTV	18.15
289	Decorin-binding protein A	O50917	DRB1*11:74	75	89	NPFILEAKVRATTVA	18.37
290	Decorin-binding protein A	O50917	DRB1*11:74	73	87	SENPFILEAKVRATT	21.33
291	Decorin-binding protein A	O50917	DRB1*11:74	72	86	VSENPFILEAKVRAT	24.51
292	Decorin-binding protein A	O50917	DRB1*11:74	76	90	PFILEAKVRATTVAE	29.02
293	Decorin-binding protein A	O50917	DRB1*11:74	6	20	NKTFNNLLKLTILVN	35.05
294	Decorin-binding protein A	O50917	DRB1*11:74	5	19	NNKTFNNLLKLTILV	42.93
295	Decorin-binding protein A	O50917	DRB1*11:74	4	18	CNNKTFNNLLKLTIL	43.3
296	Decorin-binding protein A	O50917	DRB1*11:74	71	85	GVSENPFILEAKVRA	46.31
297	Decorin-binding protein A	O50917	DRB1*13:01	151	165	QGVLEIAKKMREKLQ	32.21
298	Decorin-binding protein A	O50917	DRB1*13:01	152	166	GVLEIAKKMREKLQR	32.82
299	Decorin-binding protein A	O50917	DRB1*13:01	150	164	AQGVLEIAKKMREKL	35.62
300	Decorin-binding protein A	O50917	DRB1*13:01	153	167	VLEIAKKMREKLQRV	35.65
301	Decorin-binding protein A	O50917	DRB1*13:01	24	38	SCGLTGATKIRLERS	43.4
302	Decorin-binding protein A	O50917	DRB1*13:01	75	89	NPFILEAKVRATTVA	44.5
303	Decorin-binding protein A	O50917	DRB1*13:01	154	168	LEIAKKMREKLQRVH	48.9

304	Decorin-binding protein A	O50917	DRB1*13:05	74	88	ENPFILEAKVRATTV	18.15
305	Decorin-binding protein A	O50917	DRB1*13:05	75	89	NPFILEAKVRATTVA	18.37
306	Decorin-binding protein A	O50917	DRB1*13:05	73	87	SENPFILEAKVRATT	21.33
307	Decorin-binding protein A	O50917	DRB1*13:05	72	86	VSENPFILEAKVRAT	24.51
308	Decorin-binding protein A	O50917	DRB1*13:05	76	90	PFILEAKVRATTVAE	29.02
309	Decorin-binding protein A	O50917	DRB1*13:05	6	20	NKTFNNLLKLTILVN	35.05
310	Decorin-binding protein A	O50917	DRB1*13:05	5	19	NNKTFNNLLKLTILV	42.93
311	Decorin-binding protein A	O50917	DRB1*13:05	4	18	CNNKTFNNLLKLTIL	43.3
312	Decorin-binding protein A	O50917	DRB1*13:05	71	85	GVSENPFILEAKVRA	46.31
313	Decorin-binding protein A	O50917	DRB1*13:07	74	88	ENPFILEAKVRATTV	35.7
314	Decorin-binding protein A	O50917	DRB1*13:07	75	89	NPFILEAKVRATTVA	36.68
315	Decorin-binding protein A	O50917	DRB1*13:07	73	87	SENPFILEAKVRATT	43.13
316	Decorin-binding protein A	O50917	DRB1*13:11	151	165	QGVLEIAKKMREKLQ	18.32
317	Decorin-binding protein A	O50917	DRB1*13:11	150	164	AQGVLEIAKKMREKL	18.73
318	Decorin-binding protein A	O50917	DRB1*13:11	152	166	GVLEIAKKMREKLQR	19.2
319	Decorin-binding protein A	O50917	DRB1*13:11	75	89	NPFILEAKVRATTVA	22.38
320	Decorin-binding protein A	O50917	DRB1*13:11	74	88	ENPFILEAKVRATTV	23.48
321	Decorin-binding protein A	O50917	DRB1*13:11	153	167	VLEIAKKMREKLQRV	25.93
322	Decorin-binding protein A	O50917	DRB1*13:11	149	163	TAQGVLEIAKKMREK	30.71
323	Decorin-binding protein A	O50917	DRB1*13:11	128	142	KLGIQEMTKTVSDAA	31.36
324	Decorin-binding protein A	O50917	DRB1*13:11	76	90	PFILEAKVRATTVAE	31.53
325	Decorin-binding protein A	O50917	DRB1*13:11	73	87	SENPFILEAKVRATT	33.27
326	Decorin-binding protein A	O50917	DRB1*13:11	127	141	QKLGIQEMTKTVSDA	36.55
327	Decorin-binding protein A	O50917	DRB1*13:11	116	130	YDLMFEVSKPLQKLG	43.63
328	Decorin-binding protein A	O50917	DRB1*13:11	115	129	MYDLMFEVSKPLQKL	43.73
329	Decorin-binding protein A	O50917	DRB1*13:11	72	86	VSENPFILEAKVRAT	46.47
330	Decorin-binding protein A	O50917	DRB1*13:11	129	143	LGIQEMTKTVSDAAE	48.01
331	Decorin-binding protein A	O50917	DRB1*13:11	126	140	LQKLGIQEMTKTVSD	49.23
332	Decorin-binding protein A	O50917	DRB1*13:11	24	38	SCGLTGATKIRLERS	49.97
333	Decorin-binding protein A	O50917	DRB1*13:14	74	88	ENPFILEAKVRATTV	18.15
334	Decorin-binding protein A	O50917	DRB1*13:14	75	89	NPFILEAKVRATTVA	18.37
335	Decorin-binding protein A	O50917	DRB1*13:14	73	87	SENPFILEAKVRATT	21.33
336	Decorin-binding protein A	O50917	DRB1*13:14	72	86	VSENPFILEAKVRAT	24.51
337	Decorin-binding protein A	O50917	DRB1*13:14	76	90	PFILEAKVRATTVAE	29.02
338	Decorin-binding protein A	O50917	DRB1*13:14	6	20	NKTFNNLLKLTILVN	35.05
339	Decorin-binding protein A	O50917	DRB1*13:14	5	19	NNKTFNNLLKLTILV	42.93
340	Decorin-binding protein A	O50917	DRB1*13:14	4	18	CNNKTFNNLLKLTIL	43.3
341	Decorin-binding protein A	O50917	DRB1*13:14	71	85	GVSENPFILEAKVRA	46.31
342	Decorin-binding protein A	O50917	DRB1*13:21	150	164	AQGVLEIAKKMREKL	30.58
343	Decorin-binding protein A	O50917	DRB1*13:21	152	166	GVLEIAKKMREKLQR	32.17
344	Decorin-binding protein A	O50917	DRB1*13:21	151	165	QGVLEIAKKMREKLQ	33.18
345	Decorin-binding protein A	O50917	DRB1*13:21	74	88	ENPFILEAKVRATTV	36.61
346	Decorin-binding protein A	O50917	DRB1*13:21	75	89	NPFILEAKVRATTVA	38.86
347	Decorin-binding protein A	O50917	DRB1*13:21	3	17	KCNNKTFNNLLKLTI	41.55

348	Decorin-binding protein A	O50917	DRB1*13:21	73	87	SENPFILEAKVRATT	41.93
349	Decorin-binding protein A	O50917	DRB1*13:21	153	167	VLEIAKKMREKLQRV	42.04
350	Decorin-binding protein A	O50917	DRB1*13:21	6	20	NKTFNNLLKLTILVN	43.25
351	Decorin-binding protein A	O50917	DRB1*13:21	72	86	VSENPFILEAKVRAT	43.88
352	Decorin-binding protein A	O50917	DRB1*13:21	4	18	CNNKTFNNLLKLTIL	44.43
353	Decorin-binding protein A	O50917	DRB1*13:21	149	163	TAQGVLEIAKKMREK	47
354	Decorin-binding protein A	O50917	DRB1*13:50	74	88	ENPFILEAKVRATTV	18.15
355	Decorin-binding protein A	O50917	DRB1*13:50	75	89	NPFILEAKVRATTVA	18.37
356	Decorin-binding protein A	O50917	DRB1*13:50	73	87	SENPFILEAKVRATT	21.33
357	Decorin-binding protein A	O50917	DRB1*13:50	72	86	VSENPFILEAKVRAT	24.51
358	Decorin-binding protein A	O50917	DRB1*13:50	76	90	PFILEAKVRATTVAE	29.02
359	Decorin-binding protein A	O50917	DRB1*13:50	6	20	NKTFNNLLKLTILVN	35.05
360	Decorin-binding protein A	O50917	DRB1*13:50	5	19	NNKTFNNLLKLTILV	42.93
361	Decorin-binding protein A	O50917	DRB1*13:50	4	18	CNNKTFNNLLKLTIL	43.3
362	Decorin-binding protein A	O50917	DRB1*13:50	71	85	GVSENPFILEAKVRA	46.31
363	Decorin-binding protein A	O50917	DRB1*14:32	23	37	ISCGLTGATKIRLER	47.78
364	Decorin-binding protein A	O50917	DRB1*14:32	24	38	SCGLTGATKIRLERS	49.46
365	OppA-2	Q6RH12	DRB1*01:01	69	83	IAPIYIYGNSYLFRN	20.5
366	OppA-2	Q6RH12	DRB1*01:01	70	84	APIYIYGNSYLFRND	23.43
367	OppA-2	Q6RH12	DRB1*01:01	68	82	PIAPIYIYGNSYLFR	28.63
368	OppA-2	Q6RH12	DRB1*01:01	71	85	PIYIYGNSYLFRNDK	29.63
369	OppA-2	Q6RH12	DRB1*01:01	11	25	PLTFLSIFTQGYTQF	35.81
370	OppA-2	Q6RH12	DRB1*01:01	10	24	DPLTFLSIFTQGYTQ	38.96
371	OppA-2	Q6RH12	DRB1*01:01	12	26	LTFLSIFTQGYTQFS	46.15
372	OppA-2	Q6RH12	DRB1*01:01	13	27	TFLSIFTQGYTQFSS	48.93
373	OppA-2	Q6RH12	DRB1*01:01	9	23	ADPLTFLSIFTQGYT	49.87
374	OppA-2	Q6RH12	DRB1*01:18	69	83	IAPIYIYGNSYLFRN	17.27
375	OppA-2	Q6RH12	DRB1*01:18	70	84	APIYIYGNSYLFRND	19.8
376	OppA-2	Q6RH12	DRB1*01:18	68	82	PIAPIYIYGNSYLFR	22.41
377	OppA-2	Q6RH12	DRB1*01:18	71	85	PIYIYGNSYLFRNDK	24.99
378	OppA-2	Q6RH12	DRB1*01:18	11	25	PLTFLSIFTQGYTQF	25.28
379	OppA-2	Q6RH12	DRB1*01:18	10	24	DPLTFLSIFTQGYTQ	27.25
380	OppA-2	Q6RH12	DRB1*01:18	9	23	ADPLTFLSIFTQGYT	32.85
381	OppA-2	Q6RH12	DRB1*01:18	12	26	LTFLSIFTQGYTQFS	33.75
382	OppA-2	Q6RH12	DRB1*01:18	13	27	TFLSIFTQGYTQFSS	40.58
383	OppA-2	Q6RH12	DRB1*01:18	58	72	AEEIIIEKDFPIAPI	42.03
384	OppA-2	Q6RH12	DRB1*01:18	72	86	IYIYGNSYLFRNDKW	44.91
385	OppA-2	Q6RH12	DRB1*01:18	59	73	EEIIIEKDFPIAPIY	49.74
386	OppA-2	Q6RH12	DRB1*01:20	58	72	AEEIIIEKDFPIAPI	32.39
387	OppA-2	Q6RH12	DRB1*01:20	59	73	EEIIIEKDFPIAPIY	36.15
388	OppA-2	Q6RH12	DRB1*01:20	69	83	IAPIYIYGNSYLFRN	36.95
389	OppA-2	Q6RH12	DRB1*01:20	70	84	APIYIYGNSYLFRND	41.04
390	OppA-2	Q6RH12	DRB1*01:20	68	82	PIAPIYIYGNSYLFR	43.05
391	OppA-2	Q6RH12	DRB1*01:20	56	70	RQAEEIIIEKDFPIA	44.61

392	OppA-2	Q6RH12	DRB1*01:20	60	74	EIIIEKDFPIAPIYI	46.88
393	OppA-2	Q6RH12	DRB1*01:20	57	71	QAEEIIIEKDFPIAP	48.34
394	OppA-2	Q6RH12	DRB1*01:24	69	83	IAPIYIYGNSYLFRN	46.64
395	OppA-2	Q6RH12	DRB1*01:29	69	83	IAPIYIYGNSYLFRN	46.78
396	OppA-2	Q6RH12	DRB1*03:11	41	55	KSDLELDPIKRQDIL	45.93
397	OppA-2	Q6RH12	DRB1*04:05	11	25	PLTFLSIFTQGYTQF	47.57
398	OppA-2	Q6RH12	DRB1*04:05	10	24	DPLTFLSIFTQGYTQ	48.85
399	OppA-2	Q6RH12	DRB1*07:01	69	83	IAPIYIYGNSYLFRN	33.98
400	OppA-2	Q6RH12	DRB1*07:01	68	82	PIAPIYIYGNSYLFR	35.7
401	OppA-2	Q6RH12	DRB1*07:01	70	84	APIYIYGNSYLFRND	44.5
402	OppA-2	Q6RH12	DRB1*09:01	69	83	IAPIYIYGNSYLFRN	40.3
403	OppA-2	Q6RH12	DRB1*09:01	68	82	PIAPIYIYGNSYLFR	44.7
404	OppA-2	Q6RH12	DRB1*09:01	70	84	APIYIYGNSYLFRND	46.58
405	OppA-2	Q6RH12	DRB1*09:01	71	85	PIYIYGNSYLFRNDK	49.56
406	OppA-2	Q6RH12	DRB1*10:01	10	24	DPLTFLSIFTQGYTQ	29.56
407	OppA-2	Q6RH12	DRB1*10:01	11	25	PLTFLSIFTQGYTQF	30.71
408	OppA-2	Q6RH12	DRB1*10:01	9	23	ADPLTFLSIFTQGYT	37.01
409	OppA-2	Q6RH12	DRB1*10:01	13	27	TFLSIFTQGYTQFSS	43.89
410	OppA-2	Q6RH12	DRB1*10:01	12	26	LTFLSIFTQGYTQFS	46.19
411	OppA-2	Q6RH12	DRB1*11:42	44	58	LELDPIKRQDILRQA	46.47
412	OppA-2	Q6RH12	DRB1*15:01	69	83	IAPIYIYGNSYLFRN	9.61
413	OppA-2	Q6RH12	DRB1*15:01	70	84	APIYIYGNSYLFRND	9.74
414	OppA-2	Q6RH12	DRB1*15:01	68	82	PIAPIYIYGNSYLFR	10.04
415	OppA-2	Q6RH12	DRB1*15:01	71	85	PIYIYGNSYLFRNDK	10.86
416	OppA-2	Q6RH12	DRB1*15:01	67	81	FPIAPIYIYGNSYLF	14.51
417	OppA-2	Q6RH12	DRB1*15:01	66	80	DFPIAPIYIYGNSYL	22.43
418	OppA-2	Q6RH12	DRB1*15:01	58	72	AEEIIIEKDFPIAPI	43.54
419	OppA-2	Q6RH12	DRB1*15:01	72	86	IYIYGNSYLFRNDKW	47.55
420	OppA-2	Q6RH12	DRB1*15:01	59	73	EEIIIEKDFPIAPIY	48.91
421	OppA-2	Q6RH12	DRB1*15:02	69	83	IAPIYIYGNSYLFRN	18.25
422	OppA-2	Q6RH12	DRB1*15:02	68	82	PIAPIYIYGNSYLFR	19.48
423	OppA-2	Q6RH12	DRB1*15:02	70	84	APIYIYGNSYLFRND	21.42
424	OppA-2	Q6RH12	DRB1*15:02	71	85	PIYIYGNSYLFRNDK	28.63
425	OppA-2	Q6RH12	DRB1*15:02	67	81	FPIAPIYIYGNSYLF	29.69
426	OppA-2	Q6RH12	DRB1*15:03	70	84	APIYIYGNSYLFRND	20.09
427	OppA-2	Q6RH12	DRB1*15:03	69	83	IAPIYIYGNSYLFRN	20.24
428	OppA-2	Q6RH12	DRB1*15:03	68	82	PIAPIYIYGNSYLFR	23.04
429	OppA-2	Q6RH12	DRB1*15:03	71	85	PIYIYGNSYLFRNDK	23.19
430	OppA-2	Q6RH12	DRB1*15:03	67	81	FPIAPIYIYGNSYLF	38.58
431	OppA-2	Q6RH12	DRB1*15:06	69	83	IAPIYIYGNSYLFRN	9.61
432	OppA-2	Q6RH12	DRB1*15:06	70	84	APIYIYGNSYLFRND	9.74
433	OppA-2	Q6RH12	DRB1*15:06	68	82	PIAPIYIYGNSYLFR	10.04
434	OppA-2	Q6RH12	DRB1*15:06	71	85	PIYIYGNSYLFRNDK	10.86
435	OppA-2	Q6RH12	DRB1*15:06	67	81	FPIAPIYIYGNSYLF	14.51

436	OppA-2	Q6RH12	DRB1*15:06	66	80	DFPIAPIYIYGNSYL	22.43
437	OppA-2	Q6RH12	DRB1*15:06	58	72	AEEIIIEKDFPIAPI	43.54
438	OppA-2	Q6RH12	DRB1*15:06	72	86	IYIYGNSYLFRNDKW	47.55
439	OppA-2	Q6RH12	DRB1*15:06	59	73	EEIIIEKDFPIAPIY	48.91
440	OppA-2	Q6RH12	DRB1*15:07	69	83	IAPIYIYGNSYLFRN	14.43
441	OppA-2	Q6RH12	DRB1*15:07	70	84	APIYIYGNSYLFRND	14.95
442	OppA-2	Q6RH12	DRB1*15:07	68	82	PIAPIYIYGNSYLFR	15.62
443	OppA-2	Q6RH12	DRB1*15:07	71	85	PIYIYGNSYLFRNDK	18.14
444	OppA-2	Q6RH12	DRB1*15:07	67	81	FPIAPIYIYGNSYLF	25.55
445	OppA-2	Q6RH12	DRB1*15:07	66	80	DFPIAPIYIYGNSYL	36.56
446	OppA-2	Q6RH12	DRB1*15:15	69	83	IAPIYIYGNSYLFRN	27.08
447	OppA-2	Q6RH12	DRB1*15:15	70	84	APIYIYGNSYLFRND	29.3
448	OppA-2	Q6RH12	DRB1*15:15	68	82	PIAPIYIYGNSYLFR	33.86
449	OppA-2	Q6RH12	DRB1*15:15	71	85	PIYIYGNSYLFRNDK	41.31
450	OppA-2	Q6RH12	DRB1*15:37	69	83	IAPIYIYGNSYLFRN	16.36
451	OppA-2	Q6RH12	DRB1*15:37	68	82	PIAPIYIYGNSYLFR	17.18
452	OppA-2	Q6RH12	DRB1*15:37	70	84	APIYIYGNSYLFRND	17.69
453	OppA-2	Q6RH12	DRB1*15:37	71	85	PIYIYGNSYLFRNDK	21.84
454	OppA-2	Q6RH12	DRB1*15:37	67	81	FPIAPIYIYGNSYLF	25.72
455	OppA-2	Q6RH12	DRB1*16:02	69	83	IAPIYIYGNSYLFRN	35.71
456	OppA-2	Q6RH12	DRB1*16:02	70	84	APIYIYGNSYLFRND	42.94
457	OppA-2	Q6RH12	DRB1*16:02	68	82	PIAPIYIYGNSYLFR	45.15
458	Outer surface protein A	P0CL66	DRB1*01:01	161	175	VLKGYVLEGTLTAEK	11.08
459	Outer surface protein A	P0CL66	DRB1*01:01	162	176	LKGYVLEGTLTAEKT	11.16
460	Outer surface protein A	P0CL66	DRB1*01:01	163	177	KGYVLEGTLTAEKTT	14.35
461	Outer surface protein A	P0CL66	DRB1*01:01	160	174	EVLKGYVLEGTLTAE	18.53
462	Outer surface protein A	P0CL66	DRB1*01:01	134	148	EKIITRADGTRLEYT	23.18
463	Outer surface protein A	P0CL66	DRB1*01:01	133	147	SEKIITRADGTRLEY	25.64
464	Outer surface protein A	P0CL66	DRB1*01:01	135	149	KIITRADGTRLEYTG	26.11
465	Outer surface protein A	P0CL66	DRB1*01:01	234	248	DLVFTKENTITVQQY	26.81
466	Outer surface protein A	P0CL66	DRB1*01:01	167	181	LEGTLTAEKTTLVVK	26.85
467	Outer surface protein A	P0CL66	DRB1*01:01	235	249	LVFTKENTITVQQYD	26.93
468	Outer surface protein A	P0CL66	DRB1*01:01	164	178	GYVLEGTLTAEKTTL	28.18
469	Outer surface protein A	P0CL66	DRB1*01:01	168	182	EGTLTAEKTTLVVKE	30.55
470	Outer surface protein A	P0CL66	DRB1*01:01	1	15	MKKYLLGIGLILALI	30.7
471	Outer surface protein A	P0CL66	DRB1*01:01	169	183	GTLTAEKTTLVVKEG	32.48
472	Outer surface protein A	P0CL66	DRB1*01:01	2	16	KKYLLGIGLILALIA	34.31
473	Outer surface protein A	P0CL66	DRB1*01:01	159	173	KEVLKGYVLEGTLTA	34.75
474	Outer surface protein A	P0CL66	DRB1*01:01	233	247	KDLVFTKENTITVQQ	35.65
475	Outer surface protein A	P0CL66	DRB1*01:01	132	146	VSEKIITRADGTRLE	44.54
476	Outer surface protein A	P0CL66	DRB1*01:01	176	190	TTLVVKEGTVTLSKN	47.95
477	Outer surface protein A	P0CL66	DRB1*01:01	232	246	TKDLVFTKENTITVQ	48.81
478	Outer surface protein A	P0CL66	DRB1*01:01	175	189	KTTLVVKEGTVTLSK	49.01
479	Outer surface protein A	P0CL66	DRB1*01:02	134	148	EKIITRADGTRLEYT	46.22

480	Outer surface protein A	P0CL66	DRB1*01:11	162	176	LKGYVLEGTLTAEKT	26.28
481	Outer surface protein A	P0CL66	DRB1*01:11	161	175	VLKGYVLEGTLTAEK	28.09
482	Outer surface protein A	P0CL66	DRB1*01:11	163	177	KGYVLEGTLTAEKTT	37.55
483	Outer surface protein A	P0CL66	DRB1*01:11	1	15	MKKYLLGIGLILALI	42.86
484	Outer surface protein A	P0CL66	DRB1*01:11	2	16	KKYLLGIGLILALIA	46.12
485	Outer surface protein A	P0CL66	DRB1*01:18	161	175	VLKGYVLEGTLTAEK	14.3
486	Outer surface protein A	P0CL66	DRB1*01:18	162	176	LKGYVLEGTLTAEKT	14.73
487	Outer surface protein A	P0CL66	DRB1*01:18	163	177	KGYVLEGTLTAEKTT	19.93
488	Outer surface protein A	P0CL66	DRB1*01:18	1	15	MKKYLLGIGLILALI	21.08
489	Outer surface protein A	P0CL66	DRB1*01:18	160	174	EVLKGYVLEGTLTAE	22.7
490	Outer surface protein A	P0CL66	DRB1*01:18	2	16	KKYLLGIGLILALIA	23.79
491	Outer surface protein A	P0CL66	DRB1*01:18	134	148	EKIITRADGTRLEYT	28.62
492	Outer surface protein A	P0CL66	DRB1*01:18	7	21	GIGLILALIACKQNV	32.54
493	Outer surface protein A	P0CL66	DRB1*01:18	133	147	SEKIITRADGTRLEY	32.86
494	Outer surface protein A	P0CL66	DRB1*01:18	235	249	LVFTKENTITVQQYD	32.94
495	Outer surface protein A	P0CL66	DRB1*01:18	234	248	DLVFTKENTITVQQY	33.19
496	Outer surface protein A	P0CL66	DRB1*01:18	135	149	KIITRADGTRLEYTG	33.5
497	Outer surface protein A	P0CL66	DRB1*01:18	9	23	GLILALIACKQNVSS	35.14
498	Outer surface protein A	P0CL66	DRB1*01:18	6	20	LGIGLILALIACKQN	35.58
499	Outer surface protein A	P0CL66	DRB1*01:18	8	22	IGLILALIACKQNVS	36.09
500	Outer surface protein A	P0CL66	DRB1*01:18	159	173	KEVLKGYVLEGTLTA	37.52
501	Outer surface protein A	P0CL66	DRB1*01:18	164	178	GYVLEGTLTAEKTTL	40.92
502	Outer surface protein A	P0CL66	DRB1*01:18	233	247	KDLVFTKENTITVQQ	41.83
503	Outer surface protein A	P0CL66	DRB1*01:18	167	181	LEGTLTAEKTTLVVK	41.86
504	Outer surface protein A	P0CL66	DRB1*01:18	175	189	KTTLVVKEGTVTLSK	44.12
505	Outer surface protein A	P0CL66	DRB1*01:18	168	182	EGTLTAEKTTLVVKE	46.07
506	Outer surface protein A	P0CL66	DRB1*01:18	176	190	TTLVVKEGTVTLSKN	48.84
507	Outer surface protein A	P0CL66	DRB1*01:18	169	183	GTLTAEKTTLVVKEG	49.7
508	Outer surface protein A	P0CL66	DRB1*01:20	134	148	EKIITRADGTRLEYT	18.39
509	Outer surface protein A	P0CL66	DRB1*01:20	133	147	SEKIITRADGTRLEY	19.64
510	Outer surface protein A	P0CL66	DRB1*01:20	135	149	KIITRADGTRLEYTG	20.04
511	Outer surface protein A	P0CL66	DRB1*01:20	161	175	VLKGYVLEGTLTAEK	29.06
512	Outer surface protein A	P0CL66	DRB1*01:20	132	146	VSEKIITRADGTRLE	30.21
513	Outer surface protein A	P0CL66	DRB1*01:20	167	181	LEGTLTAEKTTLVVK	31.09
514	Outer surface protein A	P0CL66	DRB1*01:20	162	176	LKGYVLEGTLTAEKT	32.45
515	Outer surface protein A	P0CL66	DRB1*01:20	9	23	GLILALIACKQNVSS	32.55
516	Outer surface protein A	P0CL66	DRB1*01:20	168	182	EGTLTAEKTTLVVKE	34.9
517	Outer surface protein A	P0CL66	DRB1*01:20	169	183	GTLTAEKTTLVVKEG	36.55
518	Outer surface protein A	P0CL66	DRB1*01:20	176	190	TTLVVKEGTVTLSKN	37.17
519	Outer surface protein A	P0CL66	DRB1*01:20	175	189	KTTLVVKEGTVTLSK	37.24
520	Outer surface protein A	P0CL66	DRB1*01:20	8	22	IGLILALIACKQNVS	37.41
521	Outer surface protein A	P0CL66	DRB1*01:20	7	21	GIGLILALIACKQNV	39.68
522	Outer surface protein A	P0CL66	DRB1*01:20	6	20	LGIGLILALIACKQN	40.63
523	Outer surface protein A	P0CL66	DRB1*01:20	160	174	EVLKGYVLEGTLTAE	45.38

524	Outer surface protein A	P0CL66	DRB1*01:20	177	191	TLVVKEGTVTLSKNI	45.87
525	Outer surface protein A	P0CL66	DRB1*01:20	136	150	IITRADGTRLEYTGI	47.85
526	Outer surface protein A	P0CL66	DRB1*01:20	10	24	LILALIACKQNVSSL	47.94
527	Outer surface protein A	P0CL66	DRB1*01:20	163	177	KGYVLEGTLTAEKTT	49.28
528	Outer surface protein A	P0CL66	DRB1*01:24	1	15	MKKYLLGIGLILALI	25.34
529	Outer surface protein A	P0CL66	DRB1*01:24	2	16	KKYLLGIGLILALIA	25.49
530	Outer surface protein A	P0CL66	DRB1*01:24	162	176	LKGYVLEGTLTAEKT	30.78
531	Outer surface protein A	P0CL66	DRB1*01:24	161	175	VLKGYVLEGTLTAEK	31.5
532	Outer surface protein A	P0CL66	DRB1*01:24	163	177	KGYVLEGTLTAEKTT	45.41
533	Outer surface protein A	P0CL66	DRB1*01:29	162	176	LKGYVLEGTLTAEKT	28.58
534	Outer surface protein A	P0CL66	DRB1*01:29	161	175	VLKGYVLEGTLTAEK	29.43
535	Outer surface protein A	P0CL66	DRB1*01:29	163	177	KGYVLEGTLTAEKTT	40.47
536	Outer surface protein A	P0CL66	DRB1*01:29	1	15	MKKYLLGIGLILALI	45.61
537	Outer surface protein A	P0CL66	DRB1*01:29	160	174	EVLKGYVLEGTLTAE	49.94
538	Outer surface protein A	P0CL66	DRB1*03:11	147	161	YTGIKSDGSGKAKEV	40.39
539	Outer surface protein A	P0CL66	DRB1*03:11	99	113	LEVFKEDGKTLVSKK	43.53
540	Outer surface protein A	P0CL66	DRB1*03:11	98	112	TLEVFKEDGKTLVSK	47.85
541	Outer surface protein A	P0CL66	DRB1*04:01	162	176	LKGYVLEGTLTAEKT	17.83
542	Outer surface protein A	P0CL66	DRB1*04:01	161	175	VLKGYVLEGTLTAEK	17.95
543	Outer surface protein A	P0CL66	DRB1*04:01	163	177	KGYVLEGTLTAEKTT	20.88
544	Outer surface protein A	P0CL66	DRB1*04:01	160	174	EVLKGYVLEGTLTAE	25.21
545	Outer surface protein A	P0CL66	DRB1*04:01	164	178	GYVLEGTLTAEKTTL	32.51
546	Outer surface protein A	P0CL66	DRB1*04:01	159	173	KEVLKGYVLEGTLTA	40.57
547	Outer surface protein A	P0CL66	DRB1*04:08	162	176	LKGYVLEGTLTAEKT	22.86
548	Outer surface protein A	P0CL66	DRB1*04:08	161	175	VLKGYVLEGTLTAEK	22.99
549	Outer surface protein A	P0CL66	DRB1*04:08	163	177	KGYVLEGTLTAEKTT	28.7
550	Outer surface protein A	P0CL66	DRB1*04:08	160	174	EVLKGYVLEGTLTAE	29.56
551	Outer surface protein A	P0CL66	DRB1*04:08	164	178	GYVLEGTLTAEKTTL	46.23
552	Outer surface protein A	P0CL66	DRB1*04:08	159	173	KEVLKGYVLEGTLTA	48.5
553	Outer surface protein A	P0CL66	DRB1*04:72	162	176	LKGYVLEGTLTAEKT	28.45
554	Outer surface protein A	P0CL66	DRB1*04:72	161	175	VLKGYVLEGTLTAEK	28.69
555	Outer surface protein A	P0CL66	DRB1*04:72	163	177	KGYVLEGTLTAEKTT	36.94
556	Outer surface protein A	P0CL66	DRB1*04:72	160	174	EVLKGYVLEGTLTAE	39.66
557	Outer surface protein A	P0CL66	DRB1*10:01	162	176	LKGYVLEGTLTAEKT	19.36
558	Outer surface protein A	P0CL66	DRB1*10:01	161	175	VLKGYVLEGTLTAEK	19.57
559	Outer surface protein A	P0CL66	DRB1*10:01	163	177	KGYVLEGTLTAEKTT	24.12
560	Outer surface protein A	P0CL66	DRB1*10:01	160	174	EVLKGYVLEGTLTAE	29.07
561	Outer surface protein A	P0CL66	DRB1*10:01	164	178	GYVLEGTLTAEKTTL	39.7
562	Outer surface protein A	P0CL66	DRB1*10:01	159	173	KEVLKGYVLEGTLTA	48.5
563	Outer surface protein A	P0CL66	DRB1*11:03	222	236	TLTITVNSKKTKDLV	44.48
564	Outer surface protein A	P0CL66	DRB1*14:32	50	64	GKYDLIATVDKLELK	37.71
565	Outer surface protein A	P0CL66	DRB1*14:32	51	65	KYDLIATVDKLELKG	41.91
566	Outer surface protein A	P0CL66	DRB1*14:32	52	66	YDLIATVDKLELKGT	44.17
567	Outer surface protein C	Q07337	DPB1*33:01	170	184	AEELGKLFESVEVLS	35.2

568	Outer surface protein C	Q07337	DPB1*33:01	169	183	GAEELGKLFESVEVL	36.63
569	Outer surface protein C	Q07337	DPB1*33:01	171	185	EELGKLFESVEVLSK	43.24
570	Outer surface protein C	Q07337	DPB1*71:01	170	184	AEELGKLFESVEVLS	35.2
571	Outer surface protein C	Q07337	DPB1*71:01	169	183	GAEELGKLFESVEVL	36.63
572	Outer surface protein C	Q07337	DPB1*71:01	171	185	EELGKLFESVEVLSK	43.24
573	Outer surface protein C	Q07337	DRB1*01:01	46	60	SKKITDSNAVLLAVK	21.56
574	Outer surface protein C	Q07337	DRB1*01:01	95	109	GSLLAGAYAISTLIK	24.18
575	Outer surface protein C	Q07337	DRB1*01:01	47	61	KKITDSNAVLLAVKE	28.92
576	Outer surface protein C	Q07337	DRB1*01:01	45	59	ISKKITDSNAVLLAV	31.57
577	Outer surface protein C	Q07337	DRB1*01:01	93	107	HNGSLLAGAYAISTL	31.89
578	Outer surface protein C	Q07337	DRB1*01:01	94	108	NGSLLAGAYAISTLI	32.5
579	Outer surface protein C	Q07337	DRB1*01:01	96	110	SLLAGAYAISTLIKQ	33.48
580	Outer surface protein C	Q07337	DRB1*01:01	192	206	ANSVKELTSPVVAES	34.22
581	Outer surface protein C	Q07337	DRB1*01:01	92	106	NHNGSLLAGAYAIST	38.08
582	Outer surface protein C	Q07337	DRB1*01:01	191	205	LANSVKELTSPVVAE	39.48
583	Outer surface protein C	Q07337	DRB1*01:01	109	123	KQKLDGLKNEGLKEK	40.98
584	Outer surface protein C	Q07337	DRB1*01:01	97	111	LLAGAYAISTLIKQK	41.84
585	Outer surface protein C	Q07337	DRB1*01:01	190	204	MLANSVKELTSPVVA	44.76
586	Outer surface protein C	Q07337	DRB1*01:01	193	207	NSVKELTSPVVAESP	44.83
587	Outer surface protein C	Q07337	DRB1*01:01	11	25	MTLFLFISCNNSGKD	44.91
588	Outer surface protein C	Q07337	DRB1*01:01	91	105	NNHNGSLLAGAYAIS	47.67
589	Outer surface protein C	Q07337	DRB1*01:01	184	198	SKAAKEMLANSVKEL	48.05
590	Outer surface protein C	Q07337	DRB1*01:01	12	26	TLFLFISCNNSGKDG	49.13
591	Outer surface protein C	Q07337	DRB1*01:02	46	60	SKKITDSNAVLLAVK	45.25
592	Outer surface protein C	Q07337	DRB1*01:11	46	60	SKKITDSNAVLLAVK	47.46
593	Outer surface protein C	Q07337	DRB1*01:18	46	60	SKKITDSNAVLLAVK	22.02
594	Outer surface protein C	Q07337	DRB1*01:18	95	109	GSLLAGAYAISTLIK	26.41
595	Outer surface protein C	Q07337	DRB1*01:18	47	61	KKITDSNAVLLAVKE	28.6
596	Outer surface protein C	Q07337	DRB1*01:18	192	206	ANSVKELTSPVVAES	31.29
597	Outer surface protein C	Q07337	DRB1*01:18	45	59	ISKKITDSNAVLLAV	31.31
598	Outer surface protein C	Q07337	DRB1*01:18	93	107	HNGSLLAGAYAISTL	31.39
599	Outer surface protein C	Q07337	DRB1*01:18	96	110	SLLAGAYAISTLIKQ	32.75
600	Outer surface protein C	Q07337	DRB1*01:18	94	108	NGSLLAGAYAISTLI	33.29
601	Outer surface protein C	Q07337	DRB1*01:18	92	106	NHNGSLLAGAYAIST	35.55
602	Outer surface protein C	Q07337	DRB1*01:18	191	205	LANSVKELTSPVVAE	36.28
603	Outer surface protein C	Q07337	DRB1*01:18	97	111	LLAGAYAISTLIKQK	39.02
604	Outer surface protein C	Q07337	DRB1*01:18	193	207	NSVKELTSPVVAESP	40.17
605	Outer surface protein C	Q07337	DRB1*01:18	190	204	MLANSVKELTSPVVA	40.19
606	Outer surface protein C	Q07337	DRB1*01:18	11	25	MTLFLFISCNNSGKD	41.49
607	Outer surface protein C	Q07337	DRB1*01:18	109	123	KQKLDGLKNEGLKEK	42.08
608	Outer surface protein C	Q07337	DRB1*01:18	12	26	TLFLFISCNNSGKDG	45.41
609	Outer surface protein C	Q07337	DRB1*01:18	91	105	NNHNGSLLAGAYAIS	46.12
610	Outer surface protein C	Q07337	DRB1*01:18	66	80	LSSIDEIAAKAIGKK	46.49
611	Outer surface protein C	Q07337	DRB1*01:18	184	198	SKAAKEMLANSVKEL	49.27

612	Outer surface protein C	Q07337	DRB1*01:18	10	24	LMTLFLFISCNNSGK	49.44
613	Outer surface protein C	Q07337	DRB1*01:18	98	112	LAGAYAISTLIKQKL	49.67
614	Outer surface protein C	Q07337	DRB1*01:20	46	60	SKKITDSNAVLLAVK	16.27
615	Outer surface protein C	Q07337	DRB1*01:20	47	61	KKITDSNAVLLAVKE	20.42
616	Outer surface protein C	Q07337	DRB1*01:20	45	59	ISKKITDSNAVLLAV	22.57
617	Outer surface protein C	Q07337	DRB1*01:20	95	109	GSLLAGAYAISTLIK	23.21
618	Outer surface protein C	Q07337	DRB1*01:20	93	107	HNGSLLAGAYAISTL	29.45
619	Outer surface protein C	Q07337	DRB1*01:20	192	206	ANSVKELTSPVVAES	30.24
620	Outer surface protein C	Q07337	DRB1*01:20	94	108	NGSLLAGAYAISTLI	31.12
621	Outer surface protein C	Q07337	DRB1*01:20	96	110	SLLAGAYAISTLIKQ	32.35
622	Outer surface protein C	Q07337	DRB1*01:20	191	205	LANSVKELTSPVVAE	33.88
623	Outer surface protein C	Q07337	DRB1*01:20	109	123	KQKLDGLKNEGLKEK	35.06
624	Outer surface protein C	Q07337	DRB1*01:20	92	106	NHNGSLLAGAYAIST	35.7
625	Outer surface protein C	Q07337	DRB1*01:20	190	204	MLANSVKELTSPVVA	36.15
626	Outer surface protein C	Q07337	DRB1*01:20	193	207	NSVKELTSPVVAESP	36.5
627	Outer surface protein C	Q07337	DRB1*01:20	44	58	EISKKITDSNAVLLA	38.36
628	Outer surface protein C	Q07337	DRB1*01:20	97	111	LLAGAYAISTLIKQK	40.5
629	Outer surface protein C	Q07337	DRB1*01:20	66	80	LSSIDEIAAKAIGKK	43.08
630	Outer surface protein C	Q07337	DRB1*01:20	98	112	LAGAYAISTLIKQKL	44.06
631	Outer surface protein C	Q07337	DRB1*01:20	48	62	KITDSNAVLLAVKEV	47.61
632	Outer surface protein C	Q07337	DRB1*01:20	108	122	IKQKLDGLKNEGLKE	47.61
633	Outer surface protein C	Q07337	DRB1*01:20	156	170	KEAILKTNGTKTKGA	49.13
634	Outer surface protein C	Q07337	DRB1*01:20	107	121	LIKQKLDGLKNEGLK	49.71
635	Outer surface protein C	Q07337	DRB1*01:20	54	68	AVLLAVKEVEALLSS	49.88
636	Outer surface protein C	Q07337	DRB1*01:24	46	60	SKKITDSNAVLLAVK	49.91
637	Outer surface protein C	Q07337	DRB1*01:29	95	109	GSLLAGAYAISTLIK	38.9
638	Outer surface protein C	Q07337	DRB1*01:29	46	60	SKKITDSNAVLLAVK	48.5
639	Outer surface protein C	Q07337	DRB1*01:29	94	108	NGSLLAGAYAISTLI	49.31
640	Outer surface protein C	Q07337	DRB1*04:01	11	25	MTLFLFISCNNSGKD	49.37
641	Outer surface protein C	Q07337	DRB1*04:04	12	26	TLFLFISCNNSGKDG	49.63
642	Outer surface protein C	Q07337	DRB1*04:08	11	25	MTLFLFISCNNSGKD	47.57
643	Outer surface protein C	Q07337	DRB1*07:01	46	60	SKKITDSNAVLLAVK	16.69
644	Outer surface protein C	Q07337	DRB1*07:01	45	59	ISKKITDSNAVLLAV	16.78
645	Outer surface protein C	Q07337	DRB1*07:01	44	58	EISKKITDSNAVLLA	20.14
646	Outer surface protein C	Q07337	DRB1*07:01	47	61	KKITDSNAVLLAVKE	21.31
647	Outer surface protein C	Q07337	DRB1*07:01	43	57	TEISKKITDSNAVLL	28.46
648	Outer surface protein C	Q07337	DRB1*07:01	48	62	KITDSNAVLLAVKEV	48.44
649	Outer surface protein C	Q07337	DRB1*10:01	174	188	GKLFESVEVLSKAAK	41.09
650	Outer surface protein C	Q07337	DRB1*10:01	11	25	MTLFLFISCNNSGKD	44.18
651	Outer surface protein C	Q07337	DRB1*10:01	175	189	KLFESVEVLSKAAKE	44.34
652	Outer surface protein C	Q07337	DRB1*10:01	173	187	LGKLFESVEVLSKAA	45.54
653	Outer surface protein C	Q07337	DRB1*10:01	95	109	GSLLAGAYAISTLIK	45.57
654	Outer surface protein C	Q07337	DRB1*10:01	12	26	TLFLFISCNNSGKDG	49.6
655	Outer surface protein C	Q07337	DRB1*11:04	177	191	FESVEVLSKAAKEML	47.59

656	Outer surface protein C	Q07337	DRB1*11:13	101	115	AYAISTLIKQKLDGL	31.98
657	Outer surface protein C	Q07337	DRB1*11:13	100	114	GAYAISTLIKQKLDG	35.69
658	Outer surface protein C	Q07337	DRB1*11:13	99	113	AGAYAISTLIKQKLD	35.8
659	Outer surface protein C	Q07337	DRB1*11:13	102	116	YAISTLIKQKLDGLK	35.94
660	Outer surface protein C	Q07337	DRB1*11:13	98	112	LAGAYAISTLIKQKL	45.9
661	Outer surface protein C	Q07337	DRB1*11:13	178	192	ESVEVLSKAAKEMLA	46.22
662	Outer surface protein C	Q07337	DRB1*11:13	177	191	FESVEVLSKAAKEML	49.07
663	Outer surface protein C	Q07337	DRB1*11:13	179	193	SVEVLSKAAKEMLAN	49.61
664	Outer surface protein C	Q07337	DRB1*11:14	46	60	SKKITDSNAVLLAVK	15.22
665	Outer surface protein C	Q07337	DRB1*11:14	45	59	ISKKITDSNAVLLAV	18.04
666	Outer surface protein C	Q07337	DRB1*11:14	47	61	KKITDSNAVLLAVKE	18.79
667	Outer surface protein C	Q07337	DRB1*11:14	44	58	EISKKITDSNAVLLA	25.82
668	Outer surface protein C	Q07337	DRB1*11:14	48	62	KITDSNAVLLAVKEV	40.01
669	Outer surface protein C	Q07337	DRB1*11:14	43	57	TEISKKITDSNAVLL	47.33
670	Outer surface protein C	Q07337	DRB1*11:42	177	191	FESVEVLSKAAKEML	29.06
671	Outer surface protein C	Q07337	DRB1*11:42	101	115	AYAISTLIKQKLDGL	31.13
672	Outer surface protein C	Q07337	DRB1*11:42	178	192	ESVEVLSKAAKEMLA	33
673	Outer surface protein C	Q07337	DRB1*11:42	99	113	AGAYAISTLIKQKLD	33.26
674	Outer surface protein C	Q07337	DRB1*11:42	100	114	GAYAISTLIKQKLDG	35.04
675	Outer surface protein C	Q07337	DRB1*11:42	102	116	YAISTLIKQKLDGLK	35.36
676	Outer surface protein C	Q07337	DRB1*11:42	179	193	SVEVLSKAAKEMLAN	35.87
677	Outer surface protein C	Q07337	DRB1*11:42	176	190	LFESVEVLSKAAKEM	37.57
678	Outer surface protein C	Q07337	DRB1*11:42	98	112	LAGAYAISTLIKQKL	38.82
679	Outer surface protein C	Q07337	DRB1*11:42	175	189	KLFESVEVLSKAAKE	42.08
680	Outer surface protein C	Q07337	DRB1*11:42	174	188	GKLFESVEVLSKAAK	49
681	Outer surface protein C	Q07337	DRB1*11:46	177	191	FESVEVLSKAAKEML	47.59
682	Outer surface protein C	Q07337	DRB1*11:58	177	191	FESVEVLSKAAKEML	47.59
683	Outer surface protein C	Q07337	DRB1*13:02	46	60	SKKITDSNAVLLAVK	15.22
684	Outer surface protein C	Q07337	DRB1*13:02	45	59	ISKKITDSNAVLLAV	18.04
685	Outer surface protein C	Q07337	DRB1*13:02	47	61	KKITDSNAVLLAVKE	18.79
686	Outer surface protein C	Q07337	DRB1*13:02	44	58	EISKKITDSNAVLLA	25.82
687	Outer surface protein C	Q07337	DRB1*13:02	48	62	KITDSNAVLLAVKEV	40.01
688	Outer surface protein C	Q07337	DRB1*13:02	43	57	TEISKKITDSNAVLL	47.33
689	Outer surface protein C	Q07337	DRB1*13:11	177	191	FESVEVLSKAAKEML	47.59
690	Outer surface protein C	Q07337	DRB1*13:23	46	60	SKKITDSNAVLLAVK	15.22
691	Outer surface protein C	Q07337	DRB1*13:23	45	59	ISKKITDSNAVLLAV	18.04
692	Outer surface protein C	Q07337	DRB1*13:23	47	61	KKITDSNAVLLAVKE	18.79
693	Outer surface protein C	Q07337	DRB1*13:23	44	58	EISKKITDSNAVLLA	25.82
694	Outer surface protein C	Q07337	DRB1*13:23	48	62	KITDSNAVLLAVKEV	40.01
695	Outer surface protein C	Q07337	DRB1*13:23	43	57	TEISKKITDSNAVLL	47.33
696	Outer surface protein C	Q07337	DRB1*13:96	46	60	SKKITDSNAVLLAVK	30.67
697	Outer surface protein C	Q07337	DRB1*13:96	45	59	ISKKITDSNAVLLAV	36.38
698	Outer surface protein C	Q07337	DRB1*13:96	47	61	KKITDSNAVLLAVKE	39.52
699	Outer surface protein C	Q07337	DRB1*13:97	46	60	SKKITDSNAVLLAVK	15.22

700	Outer surface protein C	Q07337	DRB1*13:97	45	59	ISKKITDSNAVLLAV	18.04
701	Outer surface protein C	Q07337	DRB1*13:97	47	61	KKITDSNAVLLAVKE	18.79
702	Outer surface protein C	Q07337	DRB1*13:97	44	58	EISKKITDSNAVLLA	25.82
703	Outer surface protein C	Q07337	DRB1*13:97	48	62	KITDSNAVLLAVKEV	40.01
704	Outer surface protein C	Q07337	DRB1*13:97	43	57	TEISKKITDSNAVLL	47.33
705	Outer surface protein C	Q07337	DRB1*14:32	101	115	AYAISTLIKQKLDGL	30.2
706	Outer surface protein C	Q07337	DRB1*14:32	99	113	AGAYAISTLIKQKLD	31.95
707	Outer surface protein C	Q07337	DRB1*14:32	100	114	GAYAISTLIKQKLDG	35.19
708	Outer surface protein C	Q07337	DRB1*14:32	102	116	YAISTLIKQKLDGLK	35.46
709	Outer surface protein C	Q07337	DRB1*14:32	98	112	LAGAYAISTLIKQKL	36.18
710	Outer surface protein C	Q07337	DRB1*14:32	178	192	ESVEVLSKAAKEMLA	48.23
711	Outer surface protein C	Q07337	DRB1*14:32	179	193	SVEVLSKAAKEMLAN	48.84
712	Variable large protein	O06878	DPB1*33:01	67	81	KSDVKTYFTTVAAKL	32.11
713	Variable large protein	O06878	DPB1*33:01	68	82	SDVKTYFTTVAAKLE	36.4
714	Variable large protein	O06878	DPB1*33:01	69	83	DVKTYFTTVAAKLEK	37.08
715	Variable large protein	O06878	DPB1*33:01	70	84	VKTYFTTVAAKLEKT	38.78
716	Variable large protein	O06878	DPB1*33:01	36	50	SVIQLGNGFLDVFTS	45.2
717	Variable large protein	O06878	DPB1*33:01	35	49	QSVIQLGNGFLDVFT	46.18
718	Variable large protein	O06878	DPB1*33:01	2	16	KKISSAILLTTFFVF	49.73
719	Variable large protein	O06878	DPB1*71:01	67	81	KSDVKTYFTTVAAKL	32.11
720	Variable large protein	O06878	DPB1*71:01	68	82	SDVKTYFTTVAAKLE	36.4
721	Variable large protein	O06878	DPB1*71:01	69	83	DVKTYFTTVAAKLEK	37.08
722	Variable large protein	O06878	DPB1*71:01	70	84	VKTYFTTVAAKLEKT	38.78
723	Variable large protein	O06878	DPB1*71:01	36	50	SVIQLGNGFLDVFTS	45.2
724	Variable large protein	O06878	DPB1*71:01	35	49	QSVIQLGNGFLDVFT	46.18
725	Variable large protein	O06878	DPB1*71:01	2	16	KKISSAILLTTFFVF	49.73
726	Variable large protein	O06878	DRB1*01:01	70	84	VKTYFTTVAAKLEKT	6.41
727	Variable large protein	O06878	DRB1*01:01	69	83	DVKTYFTTVAAKLEK	6.55
728	Variable large protein	O06878	DRB1*01:01	71	85	KTYFTTVAAKLEKTK	7.12
729	Variable large protein	O06878	DRB1*01:01	68	82	SDVKTYFTTVAAKLE	8.36
730	Variable large protein	O06878	DRB1*01:01	72	86	TYFTTVAAKLEKTKT	11.31
731	Variable large protein	O06878	DRB1*01:01	278	292	DQIAAAIALRGMAKD	11.32
732	Variable large protein	O06878	DRB1*01:01	12	26	TFFVFINCKSQVADK	11.91
733	Variable large protein	O06878	DRB1*01:01	277	291	DDQIAAAIALRGMAK	12.15
734	Variable large protein	O06878	DRB1*01:01	67	81	KSDVKTYFTTVAAKL	12.18
735	Variable large protein	O06878	DRB1*01:01	13	27	FFVFINCKSQVADKD	12.61
736	Variable large protein	O06878	DRB1*01:01	314	328	ESAVRKVLGAITGLI	13.3
737	Variable large protein	O06878	DRB1*01:01	279	293	QIAAAIALRGMAKDG	15.51
738	Variable large protein	O06878	DRB1*01:01	276	290	KDDQIAAAIALRGMA	15.88
739	Variable large protein	O06878	DRB1*01:01	313	327	AESAVRKVLGAITGL	16.23
740	Variable large protein	O06878	DRB1*01:01	315	329	SAVRKVLGAITGLIG	16.27
741	Variable large protein	O06878	DRB1*01:01	318	332	RKVLGAITGLIGDAV	16.68
742	Variable large protein	O06878	DRB1*01:01	46	60	DVFTSFGGLVAEAFG	17.23
743	Variable large protein	O06878	DRB1*01:01	45	59	LDVFTSFGGLVAEAF	17.53

744	Variable large protein	O06878	DRB1*01:01	317	331	VRKVLGAITGLIGDA	18.18
745	Variable large protein	O06878	DRB1*01:01	44	58	FLDVFTSFGGLVAEA	19.18
746	Variable large protein	O06878	DRB1*01:01	316	330	AVRKVLGAITGLIGD	19.52
747	Variable large protein	O06878	DRB1*01:01	11	25	TTFFVFINCKSQVAD	19.8
748	Variable large protein	O06878	DRB1*01:01	47	61	VFTSFGGLVAEAFGF	19.94
749	Variable large protein	O06878	DRB1*01:01	14	28	FVFINCKSQVADKDD	20.04
750	Variable large protein	O06878	DRB1*01:01	48	62	FTSFGGLVAEAFGFK	23.05
751	Variable large protein	O06878	DRB1*01:01	275	289	KKDDQIAAAIALRGM	23.06
752	Variable large protein	O06878	DRB1*01:01	319	333	KVLGAITGLIGDAVS	25.33
753	Variable large protein	O06878	DRB1*01:01	312	326	AAESAVRKVLGAITG	26.11
754	Variable large protein	O06878	DRB1*01:01	228	242	GEQILSAIVTAADAA	27.22
755	Variable large protein	O06878	DRB1*01:01	180	194	GSEKLKAVAAAKGEN	28.11
756	Variable large protein	O06878	DRB1*01:01	181	195	SEKLKAVAAAKGENN	28.14
757	Variable large protein	O06878	DRB1*01:01	43	57	GFLDVFTSFGGLVAE	29.63
758	Variable large protein	O06878	DRB1*01:01	320	334	VLGAITGLIGDAVSS	31.28
759	Variable large protein	O06878	DRB1*01:01	227	241	SGEQILSAIVTAADA	34.14
760	Variable large protein	O06878	DRB1*01:01	179	193	GGSEKLKAVAAAKGE	34.6
761	Variable large protein	O06878	DRB1*01:01	34	48	YQSVIQLGNGFLDVF	34.71
762	Variable large protein	O06878	DRB1*01:01	229	243	EQILSAIVTAADAAE	35.93
763	Variable large protein	O06878	DRB1*01:01	321	335	LGAITGLIGDAVSSG	36.38
764	Variable large protein	O06878	DRB1*01:01	182	196	EKLKAVAAAKGENNK	36.6
765	Variable large protein	O06878	DRB1*01:01	33	47	FYQSVIQLGNGFLDV	37.99
766	Variable large protein	O06878	DRB1*01:01	32	46	KFYQSVIQLGNGFLD	40.82
767	Variable large protein	O06878	DRB1*01:01	73	87	YFTTVAAKLEKTKTD	40.93
768	Variable large protein	O06878	DRB1*01:01	31	45	NKFYQSVIQLGNGFL	42.59
769	Variable large protein	O06878	DRB1*01:01	30	44	TNKFYQSVIQLGNGF	42.99
770	Variable large protein	O06878	DRB1*01:01	49	63	TSFGGLVAEAFGFKS	44.67
771	Variable large protein	O06878	DRB1*01:01	10	24	LTTFFVFINCKSQVA	46.26
772	Variable large protein	O06878	DRB1*01:01	226	240	VSGEQILSAIVTAAD	46.97
773	Variable large protein	O06878	DRB1*01:01	15	29	VFINCKSQVADKDDP	47.04
774	Variable large protein	O06878	DRB1*01:01	35	49	QSVIQLGNGFLDVFT	49.09
775	Variable large protein	O06878	DRB1*01:01	274	288	MKKDDQIAAAIALRG	49.8
776	Variable large protein	O06878	DRB1*01:02	278	292	DQIAAAIALRGMAKD	22.12
777	Variable large protein	O06878	DRB1*01:02	277	291	DDQIAAAIALRGMAK	23.19
778	Variable large protein	O06878	DRB1*01:02	279	293	QIAAAIALRGMAKDG	25.3
779	Variable large protein	O06878	DRB1*01:02	276	290	KDDQIAAAIALRGMA	32.1
780	Variable large protein	O06878	DRB1*01:02	314	328	ESAVRKVLGAITGLI	34.89
781	Variable large protein	O06878	DRB1*01:02	315	329	SAVRKVLGAITGLIG	35.48
782	Variable large protein	O06878	DRB1*01:02	318	332	RKVLGAITGLIGDAV	40.73
783	Variable large protein	O06878	DRB1*01:02	316	330	AVRKVLGAITGLIGD	42.03
784	Variable large protein	O06878	DRB1*01:02	317	331	VRKVLGAITGLIGDA	44.44
785	Variable large protein	O06878	DRB1*01:02	275	289	KKDDQIAAAIALRGM	45.83
786	Variable large protein	O06878	DRB1*01:02	313	327	AESAVRKVLGAITGL	47.37
787	Variable large protein	O06878	DRB1*01:11	70	84	VKTYFTTVAAKLEKT	13.24

788	Variable large protein	O06878	DRB1*01:11	69	83	DVKTYFTTVAAKLEK	13.9
789	Variable large protein	O06878	DRB1*01:11	71	85	KTYFTTVAAKLEKTK	16.59
790	Variable large protein	O06878	DRB1*01:11	68	82	SDVKTYFTTVAAKLE	20.65
791	Variable large protein	O06878	DRB1*01:11	72	86	TYFTTVAAKLEKTKT	33.05
792	Variable large protein	O06878	DRB1*01:11	12	26	TFFVFINCKSQVADK	33.96
793	Variable large protein	O06878	DRB1*01:11	67	81	KSDVKTYFTTVAAKL	34.68
794	Variable large protein	O06878	DRB1*01:11	13	27	FFVFINCKSQVADKD	37.59
795	Variable large protein	O06878	DRB1*01:11	278	292	DQIAAAIALRGMAKD	39.47
796	Variable large protein	O06878	DRB1*01:11	277	291	DDQIAAAIALRGMAK	40.01
797	Variable large protein	O06878	DRB1*01:11	314	328	ESAVRKVLGAITGLI	43.01
798	Variable large protein	O06878	DRB1*01:11	315	329	SAVRKVLGAITGLIG	49.51
799	Variable large protein	O06878	DRB1*01:18	70	84	VKTYFTTVAAKLEKT	6.06
800	Variable large protein	O06878	DRB1*01:18	69	83	DVKTYFTTVAAKLEK	6.18
801	Variable large protein	O06878	DRB1*01:18	71	85	KTYFTTVAAKLEKTK	6.8
802	Variable large protein	O06878	DRB1*01:18	68	82	SDVKTYFTTVAAKLE	7.93
803	Variable large protein	O06878	DRB1*01:18	72	86	TYFTTVAAKLEKTKT	10.49
804	Variable large protein	O06878	DRB1*01:18	278	292	DQIAAAIALRGMAKD	11.22
805	Variable large protein	O06878	DRB1*01:18	67	81	KSDVKTYFTTVAAKL	11.79
806	Variable large protein	O06878	DRB1*01:18	277	291	DDQIAAAIALRGMAK	12.06
807	Variable large protein	O06878	DRB1*01:18	314	328	ESAVRKVLGAITGLI	12.31
808	Variable large protein	O06878	DRB1*01:18	12	26	TFFVFINCKSQVADK	12.69
809	Variable large protein	O06878	DRB1*01:18	45	59	LDVFTSFGGLVAEAF	13.26
810	Variable large protein	O06878	DRB1*01:18	13	27	FFVFINCKSQVADKD	13.76
811	Variable large protein	O06878	DRB1*01:18	46	60	DVFTSFGGLVAEAFG	14.02
812	Variable large protein	O06878	DRB1*01:18	315	329	SAVRKVLGAITGLIG	14.4
813	Variable large protein	O06878	DRB1*01:18	279	293	QIAAAIALRGMAKDG	14.54
814	Variable large protein	O06878	DRB1*01:18	313	327	AESAVRKVLGAITGL	15.4
815	Variable large protein	O06878	DRB1*01:18	276	290	KDDQIAAAIALRGMA	15.57
816	Variable large protein	O06878	DRB1*01:18	44	58	FLDVFTSFGGLVAEA	15.84
817	Variable large protein	O06878	DRB1*01:18	47	61	VFTSFGGLVAEAFGF	16.3
818	Variable large protein	O06878	DRB1*01:18	318	332	RKVLGAITGLIGDAV	17.06
819	Variable large protein	O06878	DRB1*01:18	317	331	VRKVLGAITGLIGDA	17.11
820	Variable large protein	O06878	DRB1*01:18	316	330	AVRKVLGAITGLIGD	17.42
821	Variable large protein	O06878	DRB1*01:18	11	25	TTFFVFINCKSQVAD	18.77
822	Variable large protein	O06878	DRB1*01:18	275	289	KKDDQIAAAIALRGM	20.61
823	Variable large protein	O06878	DRB1*01:18	48	62	FTSFGGLVAEAFGFK	20.72
824	Variable large protein	O06878	DRB1*01:18	14	28	FVFINCKSQVADKDD	21.68
825	Variable large protein	O06878	DRB1*01:18	43	57	GFLDVFTSFGGLVAE	22.8
826	Variable large protein	O06878	DRB1*01:18	312	326	AAESAVRKVLGAITG	24.39
827	Variable large protein	O06878	DRB1*01:18	319	333	KVLGAITGLIGDAVS	25.39
828	Variable large protein	O06878	DRB1*01:18	181	195	SEKLKAVAAAKGENN	26.08
829	Variable large protein	O06878	DRB1*01:18	180	194	GSEKLKAVAAAKGEN	27.01
830	Variable large protein	O06878	DRB1*01:18	34	48	YQSVIQLGNGFLDVF	28.1
831	Variable large protein	O06878	DRB1*01:18	228	242	GEQILSAIVTAADAA	30.49

832	Variable large protein	O06878	DRB1*01:18	320	334	VLGAITGLIGDAVSS	30.59
833	Variable large protein	O06878	DRB1*01:18	33	47	FYQSVIQLGNGFLDV	31.2
834	Variable large protein	O06878	DRB1*01:18	182	196	EKLKAVAAAKGENNK	33.86
835	Variable large protein	O06878	DRB1*01:18	179	193	GGSEKLKAVAAAKGE	33.96
836	Variable large protein	O06878	DRB1*01:18	32	46	KFYQSVIQLGNGFLD	34
837	Variable large protein	O06878	DRB1*01:18	31	45	NKFYQSVIQLGNGFL	34.25
838	Variable large protein	O06878	DRB1*01:18	321	335	LGAITGLIGDAVSSG	35.3
839	Variable large protein	O06878	DRB1*01:18	227	241	SGEQILSAIVTAADA	36.15
840	Variable large protein	O06878	DRB1*01:18	10	24	LTTFFVFINCKSQVA	36.23
841	Variable large protein	O06878	DRB1*01:18	30	44	TNKFYQSVIQLGNGF	37.15
842	Variable large protein	O06878	DRB1*01:18	35	49	QSVIQLGNGFLDVFT	37.39
843	Variable large protein	O06878	DRB1*01:18	49	63	TSFGGLVAEAFGFKS	39.26
844	Variable large protein	O06878	DRB1*01:18	229	243	EQILSAIVTAADAAE	39.39
845	Variable large protein	O06878	DRB1*01:18	42	56	NGFLDVFTSFGGLVA	42.46
846	Variable large protein	O06878	DRB1*01:18	73	87	YFTTVAAKLEKTKTD	42.7
847	Variable large protein	O06878	DRB1*01:18	274	288	MKKDDQIAAAIALRG	43.78
848	Variable large protein	O06878	DRB1*01:18	226	240	VSGEQILSAIVTAAD	44.47
849	Variable large protein	O06878	DRB1*01:18	225	239	AVSGEQILSAIVTAA	48.82
850	Variable large protein	O06878	DRB1*01:20	278	292	DQIAAAIALRGMAKD	8.36
851	Variable large protein	O06878	DRB1*01:20	277	291	DDQIAAAIALRGMAK	9.38
852	Variable large protein	O06878	DRB1*01:20	279	293	QIAAAIALRGMAKDG	10.24
853	Variable large protein	O06878	DRB1*01:20	276	290	KDDQIAAAIALRGMA	11.49
854	Variable large protein	O06878	DRB1*01:20	314	328	ESAVRKVLGAITGLI	11.69
855	Variable large protein	O06878	DRB1*01:20	318	332	RKVLGAITGLIGDAV	12.47
856	Variable large protein	O06878	DRB1*01:20	317	331	VRKVLGAITGLIGDA	13.07
857	Variable large protein	O06878	DRB1*01:20	315	329	SAVRKVLGAITGLIG	13.42
858	Variable large protein	O06878	DRB1*01:20	316	330	AVRKVLGAITGLIGD	14.44
859	Variable large protein	O06878	DRB1*01:20	313	327	AESAVRKVLGAITGL	15.15
860	Variable large protein	O06878	DRB1*01:20	275	289	KKDDQIAAAIALRGM	15.42
861	Variable large protein	O06878	DRB1*01:20	319	333	KVLGAITGLIGDAVS	16.89
862	Variable large protein	O06878	DRB1*01:20	34	48	YQSVIQLGNGFLDVF	18.8
863	Variable large protein	O06878	DRB1*01:20	33	47	FYQSVIQLGNGFLDV	21.06
864	Variable large protein	O06878	DRB1*01:20	320	334	VLGAITGLIGDAVSS	22.5
865	Variable large protein	O06878	DRB1*01:20	228	242	GEQILSAIVTAADAA	22.79
866	Variable large protein	O06878	DRB1*01:20	312	326	AAESAVRKVLGAITG	23.51
867	Variable large protein	O06878	DRB1*01:20	35	49	QSVIQLGNGFLDVFT	23.66
868	Variable large protein	O06878	DRB1*01:20	69	83	DVKTYFTTVAAKLEK	26.21
869	Variable large protein	O06878	DRB1*01:20	70	84	VKTYFTTVAAKLEKT	26.32
870	Variable large protein	O06878	DRB1*01:20	321	335	LGAITGLIGDAVSSG	26.36
871	Variable large protein	O06878	DRB1*01:20	227	241	SGEQILSAIVTAADA	28.16
872	Variable large protein	O06878	DRB1*01:20	180	194	GSEKLKAVAAAKGEN	28.26
873	Variable large protein	O06878	DRB1*01:20	229	243	EQILSAIVTAADAAE	28.67
874	Variable large protein	O06878	DRB1*01:20	181	195	SEKLKAVAAAKGENN	29.42
875	Variable large protein	O06878	DRB1*01:20	32	46	KFYQSVIQLGNGFLD	29.64

876	Variable large protein	O06878	DRB1*01:20	71	85	KTYFTTVAAKLEKTK	29.95
877	Variable large protein	O06878	DRB1*01:20	179	193	GGSEKLKAVAAAKGE	32.18
878	Variable large protein	O06878	DRB1*01:20	182	196	EKLKAVAAAKGENNK	32.42
879	Variable large protein	O06878	DRB1*01:20	12	26	TFFVFINCKSQVADK	33.55
880	Variable large protein	O06878	DRB1*01:20	68	82	SDVKTYFTTVAAKLE	34.46
881	Variable large protein	O06878	DRB1*01:20	13	27	FFVFINCKSQVADKD	35.87
882	Variable large protein	O06878	DRB1*01:20	274	288	MKKDDQIAAAIALRG	36.41
883	Variable large protein	O06878	DRB1*01:20	280	294	IAAAIALRGMAKDGK	37.63
884	Variable large protein	O06878	DRB1*01:20	67	81	KSDVKTYFTTVAAKL	38.86
885	Variable large protein	O06878	DRB1*01:20	322	336	GAITGLIGDAVSSGL	39.51
886	Variable large protein	O06878	DRB1*01:20	44	58	FLDVFTSFGGLVAEA	40.53
887	Variable large protein	O06878	DRB1*01:20	226	240	VSGEQILSAIVTAAD	41.32
888	Variable large protein	O06878	DRB1*01:20	183	197	KLKAVAAAKGENNKG	41.66
889	Variable large protein	O06878	DRB1*01:20	45	59	LDVFTSFGGLVAEAF	41.91
890	Variable large protein	O06878	DRB1*01:20	46	60	DVFTSFGGLVAEAFG	42.34
891	Variable large protein	O06878	DRB1*01:20	72	86	TYFTTVAAKLEKTKT	47.09
892	Variable large protein	O06878	DRB1*01:20	311	325	GAAESAVRKVLGAIT	47.35
893	Variable large protein	O06878	DRB1*01:20	230	244	QILSAIVTAADAAEQ	47.42
894	Variable large protein	O06878	DRB1*01:20	36	50	SVIQLGNGFLDVFTS	47.97
895	Variable large protein	O06878	DRB1*01:20	47	61	VFTSFGGLVAEAFGF	48.13
896	Variable large protein	O06878	DRB1*01:24	70	84	VKTYFTTVAAKLEKT	12.41
897	Variable large protein	O06878	DRB1*01:24	69	83	DVKTYFTTVAAKLEK	12.98
898	Variable large protein	O06878	DRB1*01:24	71	85	KTYFTTVAAKLEKTK	14.94
899	Variable large protein	O06878	DRB1*01:24	68	82	SDVKTYFTTVAAKLE	17.91
900	Variable large protein	O06878	DRB1*01:24	67	81	KSDVKTYFTTVAAKL	27.24
901	Variable large protein	O06878	DRB1*01:24	12	26	TFFVFINCKSQVADK	27.42
902	Variable large protein	O06878	DRB1*01:24	72	86	TYFTTVAAKLEKTKT	28.06
903	Variable large protein	O06878	DRB1*01:24	314	328	ESAVRKVLGAITGLI	28.07
904	Variable large protein	O06878	DRB1*01:24	278	292	DQIAAAIALRGMAKD	28.9
905	Variable large protein	O06878	DRB1*01:24	315	329	SAVRKVLGAITGLIG	30
906	Variable large protein	O06878	DRB1*01:24	277	291	DDQIAAAIALRGMAK	30.48
907	Variable large protein	O06878	DRB1*01:24	13	27	FFVFINCKSQVADKD	33.23
908	Variable large protein	O06878	DRB1*01:24	279	293	QIAAAIALRGMAKDG	33.32
909	Variable large protein	O06878	DRB1*01:24	45	59	LDVFTSFGGLVAEAF	37.16
910	Variable large protein	O06878	DRB1*01:24	276	290	KDDQIAAAIALRGMA	42.56
911	Variable large protein	O06878	DRB1*01:24	11	25	TTFFVFINCKSQVAD	42.93
912	Variable large protein	O06878	DRB1*01:24	313	327	AESAVRKVLGAITGL	43.03
913	Variable large protein	O06878	DRB1*01:24	316	330	AVRKVLGAITGLIGD	43.39
914	Variable large protein	O06878	DRB1*01:24	46	60	DVFTSFGGLVAEAFG	43.75
915	Variable large protein	O06878	DRB1*01:24	318	332	RKVLGAITGLIGDAV	45.13
916	Variable large protein	O06878	DRB1*01:24	47	61	VFTSFGGLVAEAFGF	45.17
917	Variable large protein	O06878	DRB1*01:24	44	58	FLDVFTSFGGLVAEA	46.41
918	Variable large protein	O06878	DRB1*01:24	317	331	VRKVLGAITGLIGDA	49
919	Variable large protein	O06878	DRB1*01:29	70	84	VKTYFTTVAAKLEKT	12.01

920	Variable large protein	O06878	DRB1*01:29	69	83	DVKTYFTTVAAKLEK	12.56
921	Variable large protein	O06878	DRB1*01:29	71	85	KTYFTTVAAKLEKTK	14.14
922	Variable large protein	O06878	DRB1*01:29	68	82	SDVKTYFTTVAAKLE	17.68
923	Variable large protein	O06878	DRB1*01:29	278	292	DQIAAAIALRGMAKD	22.63
924	Variable large protein	O06878	DRB1*01:29	277	291	DDQIAAAIALRGMAK	22.65
925	Variable large protein	O06878	DRB1*01:29	72	86	TYFTTVAAKLEKTKT	23.59
926	Variable large protein	O06878	DRB1*01:29	314	328	ESAVRKVLGAITGLI	24.69
927	Variable large protein	O06878	DRB1*01:29	12	26	TFFVFINCKSQVADK	24.82
928	Variable large protein	O06878	DRB1*01:29	67	81	KSDVKTYFTTVAAKL	26.56
929	Variable large protein	O06878	DRB1*01:29	13	27	FFVFINCKSQVADKD	27.51
930	Variable large protein	O06878	DRB1*01:29	315	329	SAVRKVLGAITGLIG	27.7
931	Variable large protein	O06878	DRB1*01:29	279	293	QIAAAIALRGMAKDG	29.35
932	Variable large protein	O06878	DRB1*01:29	276	290	KDDQIAAAIALRGMA	30.36
933	Variable large protein	O06878	DRB1*01:29	313	327	AESAVRKVLGAITGL	33.54
934	Variable large protein	O06878	DRB1*01:29	318	332	RKVLGAITGLIGDAV	33.81
935	Variable large protein	O06878	DRB1*01:29	317	331	VRKVLGAITGLIGDA	34.66
936	Variable large protein	O06878	DRB1*01:29	46	60	DVFTSFGGLVAEAFG	35.09
937	Variable large protein	O06878	DRB1*01:29	316	330	AVRKVLGAITGLIGD	35.23
938	Variable large protein	O06878	DRB1*01:29	45	59	LDVFTSFGGLVAEAF	35.68
939	Variable large protein	O06878	DRB1*01:29	47	61	VFTSFGGLVAEAFGF	38.1
940	Variable large protein	O06878	DRB1*01:29	44	58	FLDVFTSFGGLVAEA	38.74
941	Variable large protein	O06878	DRB1*01:29	275	289	KKDDQIAAAIALRGM	42.12
942	Variable large protein	O06878	DRB1*01:29	11	25	TTFFVFINCKSQVAD	42.41
943	Variable large protein	O06878	DRB1*01:29	48	62	FTSFGGLVAEAFGFK	44.16
944	Variable large protein	O06878	DRB1*01:29	14	28	FVFINCKSQVADKDD	48.08
945	Variable large protein	O06878	DRB1*03:01	284	298	IALRGMAKDGKFAVK	42.11
946	Variable large protein	O06878	DRB1*03:01	285	299	ALRGMAKDGKFAVKD	48.83
947	Variable large protein	O06878	DRB1*03:04	284	298	IALRGMAKDGKFAVK	42.11
948	Variable large protein	O06878	DRB1*03:04	285	299	ALRGMAKDGKFAVKD	48.83
949	Variable large protein	O06878	DRB1*03:11	284	298	IALRGMAKDGKFAVK	20.51
950	Variable large protein	O06878	DRB1*03:11	285	299	ALRGMAKDGKFAVKD	21.92
951	Variable large protein	O06878	DRB1*03:11	286	300	LRGMAKDGKFAVKDG	24.4
952	Variable large protein	O06878	DRB1*03:11	287	301	RGMAKDGKFAVKDGE	34.9
953	Variable large protein	O06878	DRB1*03:11	283	297	AIALRGMAKDGKFAV	40.75
954	Variable large protein	O06878	DRB1*03:13	284	298	IALRGMAKDGKFAVK	42.11
955	Variable large protein	O06878	DRB1*03:13	285	299	ALRGMAKDGKFAVKD	48.83
956	Variable large protein	O06878	DRB1*04:01	12	26	TFFVFINCKSQVADK	35.76
957	Variable large protein	O06878	DRB1*04:01	13	27	FFVFINCKSQVADKD	40.28
958	Variable large protein	O06878	DRB1*04:04	228	242	GEQILSAIVTAADAA	30.46
959	Variable large protein	O06878	DRB1*04:04	229	243	EQILSAIVTAADAAE	34.73
960	Variable large protein	O06878	DRB1*04:04	227	241	SGEQILSAIVTAADA	38.1
961	Variable large protein	O06878	DRB1*04:04	278	292	DQIAAAIALRGMAKD	39.83
962	Variable large protein	O06878	DRB1*04:04	277	291	DDQIAAAIALRGMAK	42.55
963	Variable large protein	O06878	DRB1*04:08	12	26	TFFVFINCKSQVADK	29.89

964	Variable large protein	O06878	DRB1*04:08	13	27	FFVFINCKSQVADKD	35.57
965	Variable large protein	O06878	DRB1*04:08	11	25	TTFFVFINCKSQVAD	37.71
966	Variable large protein	O06878	DRB1*04:08	228	242	GEQILSAIVTAADAA	48.36
967	Variable large protein	O06878	DRB1*07:01	67	81	KSDVKTYFTTVAAKL	7.02
968	Variable large protein	O06878	DRB1*07:01	68	82	SDVKTYFTTVAAKLE	8.45
969	Variable large protein	O06878	DRB1*07:01	69	83	DVKTYFTTVAAKLEK	8.51
970	Variable large protein	O06878	DRB1*07:01	70	84	VKTYFTTVAAKLEKT	9.33
971	Variable large protein	O06878	DRB1*07:01	71	85	KTYFTTVAAKLEKTK	10.2
972	Variable large protein	O06878	DRB1*07:01	72	86	TYFTTVAAKLEKTKT	14.53
973	Variable large protein	O06878	DRB1*07:01	314	328	ESAVRKVLGAITGLI	42.94
974	Variable large protein	O06878	DRB1*08:04	278	292	DQIAAAIALRGMAKD	32.53
975	Variable large protein	O06878	DRB1*08:04	277	291	DDQIAAAIALRGMAK	36.27
976	Variable large protein	O06878	DRB1*08:04	279	293	QIAAAIALRGMAKDG	42.99
977	Variable large protein	O06878	DRB1*09:01	69	83	DVKTYFTTVAAKLEK	23.87
978	Variable large protein	O06878	DRB1*09:01	67	81	KSDVKTYFTTVAAKL	25.36
979	Variable large protein	O06878	DRB1*09:01	70	84	VKTYFTTVAAKLEKT	25.96
980	Variable large protein	O06878	DRB1*09:01	68	82	SDVKTYFTTVAAKLE	26.64
981	Variable large protein	O06878	DRB1*09:01	71	85	KTYFTTVAAKLEKTK	29.28
982	Variable large protein	O06878	DRB1*09:01	315	329	SAVRKVLGAITGLIG	36.93
983	Variable large protein	O06878	DRB1*09:01	314	328	ESAVRKVLGAITGLI	40.25
984	Variable large protein	O06878	DRB1*09:01	72	86	TYFTTVAAKLEKTKT	40.53
985	Variable large protein	O06878	DRB1*09:01	316	330	AVRKVLGAITGLIGD	40.99
986	Variable large protein	O06878	DRB1*09:01	317	331	VRKVLGAITGLIGDA	41.93
987	Variable large protein	O06878	DRB1*10:01	69	83	DVKTYFTTVAAKLEK	12.16
988	Variable large protein	O06878	DRB1*10:01	70	84	VKTYFTTVAAKLEKT	12.18
989	Variable large protein	O06878	DRB1*10:01	71	85	KTYFTTVAAKLEKTK	13
990	Variable large protein	O06878	DRB1*10:01	68	82	SDVKTYFTTVAAKLE	15.52
991	Variable large protein	O06878	DRB1*10:01	30	44	TNKFYQSVIQLGNGF	16.23
992	Variable large protein	O06878	DRB1*10:01	31	45	NKFYQSVIQLGNGFL	16.95
993	Variable large protein	O06878	DRB1*10:01	278	292	DQIAAAIALRGMAKD	19.44
994	Variable large protein	O06878	DRB1*10:01	72	86	TYFTTVAAKLEKTKT	19.92
995	Variable large protein	O06878	DRB1*10:01	29	43	PTNKFYQSVIQLGNG	20.71
996	Variable large protein	O06878	DRB1*10:01	67	81	KSDVKTYFTTVAAKL	21.05
997	Variable large protein	O06878	DRB1*10:01	277	291	DDQIAAAIALRGMAK	21.23
998	Variable large protein	O06878	DRB1*10:01	228	242	GEQILSAIVTAADAA	22.14
999	Variable large protein	O06878	DRB1*10:01	32	46	KFYQSVIQLGNGFLD	23.5
1000	Variable large protein	O06878	DRB1*10:01	12	26	TFFVFINCKSQVADK	23.56
1001	Variable large protein	O06878	DRB1*10:01	229	243	EQILSAIVTAADAAE	24.66
1002	Variable large protein	O06878	DRB1*10:01	28	42	DPTNKFYQSVIQLGN	25.36
1003	Variable large protein	O06878	DRB1*10:01	279	293	QIAAAIALRGMAKDG	25.55
1004	Variable large protein	O06878	DRB1*10:01	13	27	FFVFINCKSQVADKD	26.02
1005	Variable large protein	O06878	DRB1*10:01	276	290	KDDQIAAAIALRGMA	26.92
1006	Variable large protein	O06878	DRB1*10:01	46	60	DVFTSFGGLVAEAFG	28.38
1007	Variable large protein	O06878	DRB1*10:01	227	241	SGEQILSAIVTAADA	28.51

1008	Variable large protein	O06878	DRB1*10:01	47	61	VFTSFGGLVAEAFGF	28.84
1009	Variable large protein	O06878	DRB1*10:01	48	62	FTSFGGLVAEAFGFK	29.64
1010	Variable large protein	O06878	DRB1*10:01	11	25	TTFFVFINCKSQVAD	33.04
1011	Variable large protein	O06878	DRB1*10:01	45	59	LDVFTSFGGLVAEAF	34.75
1012	Variable large protein	O06878	DRB1*10:01	230	244	QILSAIVTAADAAEQ	35.54
1013	Variable large protein	O06878	DRB1*10:01	275	289	KKDDQIAAAIALRGM	36.03
1014	Variable large protein	O06878	DRB1*10:01	318	332	RKVLGAITGLIGDAV	38.58
1015	Variable large protein	O06878	DRB1*10:01	315	329	SAVRKVLGAITGLIG	38.63
1016	Variable large protein	O06878	DRB1*10:01	317	331	VRKVLGAITGLIGDA	38.69
1017	Variable large protein	O06878	DRB1*10:01	316	330	AVRKVLGAITGLIGD	39.05
1018	Variable large protein	O06878	DRB1*10:01	314	328	ESAVRKVLGAITGLI	39.68
1019	Variable large protein	O06878	DRB1*10:01	226	240	VSGEQILSAIVTAAD	45.14
1020	Variable large protein	O06878	DRB1*10:01	73	87	YFTTVAAKLEKTKTD	46.67
1021	Variable large protein	O06878	DRB1*10:01	14	28	FVFINCKSQVADKDD	48.73
1022	Variable large protein	O06878	DRB1*11:03	161	175	KASVKGIAKGIKEIV	41.28
1023	Variable large protein	O06878	DRB1*11:04	278	292	DQIAAAIALRGMAKD	26.14
1024	Variable large protein	O06878	DRB1*11:04	277	291	DDQIAAAIALRGMAK	28.7
1025	Variable large protein	O06878	DRB1*11:04	279	293	QIAAAIALRGMAKDG	33.96
1026	Variable large protein	O06878	DRB1*11:04	161	175	KASVKGIAKGIKEIV	36.44
1027	Variable large protein	O06878	DRB1*11:04	122	136	VSELLDKLVKAVKTA	45.01
1028	Variable large protein	O06878	DRB1*11:04	125	139	LLDKLVKAVKTAEGA	48.95
1029	Variable large protein	O06878	DRB1*11:13	277	291	DDQIAAAIALRGMAK	46.09
1030	Variable large protein	O06878	DRB1*11:13	278	292	DQIAAAIALRGMAKD	49.38
1031	Variable large protein	O06878	DRB1*11:42	277	291	DDQIAAAIALRGMAK	20.58
1032	Variable large protein	O06878	DRB1*11:42	278	292	DQIAAAIALRGMAKD	20.69
1033	Variable large protein	O06878	DRB1*11:42	279	293	QIAAAIALRGMAKDG	29.41
1034	Variable large protein	O06878	DRB1*11:42	122	136	VSELLDKLVKAVKTA	31.84
1035	Variable large protein	O06878	DRB1*11:42	161	175	KASVKGIAKGIKEIV	32.06
1036	Variable large protein	O06878	DRB1*11:42	276	290	KDDQIAAAIALRGMA	32.84
1037	Variable large protein	O06878	DRB1*11:42	123	137	SELLDKLVKAVKTAE	36
1038	Variable large protein	O06878	DRB1*11:42	121	135	EVSELLDKLVKAVKT	37.76
1039	Variable large protein	O06878	DRB1*11:42	124	138	ELLDKLVKAVKTAEG	38.04
1040	Variable large protein	O06878	DRB1*11:42	125	139	LLDKLVKAVKTAEGA	38.34
1041	Variable large protein	O06878	DRB1*11:42	162	176	ASVKGIAKGIKEIVE	42.29
1042	Variable large protein	O06878	DRB1*11:42	120	134	KEVSELLDKLVKAVK	46.66
1043	Variable large protein	O06878	DRB1*11:42	160	174	DKASVKGIAKGIKEI	46.67
1044	Variable large protein	O06878	DRB1*11:46	278	292	DQIAAAIALRGMAKD	26.14
1045	Variable large protein	O06878	DRB1*11:46	277	291	DDQIAAAIALRGMAK	28.7
1046	Variable large protein	O06878	DRB1*11:46	279	293	QIAAAIALRGMAKDG	33.96
1047	Variable large protein	O06878	DRB1*11:46	161	175	KASVKGIAKGIKEIV	36.44
1048	Variable large protein	O06878	DRB1*11:46	122	136	VSELLDKLVKAVKTA	45.01
1049	Variable large protein	O06878	DRB1*11:46	125	139	LLDKLVKAVKTAEGA	48.95
1050	Variable large protein	O06878	DRB1*11:58	278	292	DQIAAAIALRGMAKD	26.14
1051	Variable large protein	O06878	DRB1*11:58	277	291	DDQIAAAIALRGMAK	28.7

1052	Variable large protein	O06878	DRB1*11:58	279	293	QIAAAIALRGMAKDG	33.96
1053	Variable large protein	O06878	DRB1*11:58	161	175	KASVKGIAKGIKEIV	36.44
1054	Variable large protein	O06878	DRB1*11:58	122	136	VSELLDKLVKAVKTA	45.01
1055	Variable large protein	O06878	DRB1*11:58	125	139	LLDKLVKAVKTAEGA	48.95
1056	Variable large protein	O06878	DRB1*13:11	278	292	DQIAAAIALRGMAKD	26.14
1057	Variable large protein	O06878	DRB1*13:11	277	291	DDQIAAAIALRGMAK	28.7
1058	Variable large protein	O06878	DRB1*13:11	279	293	QIAAAIALRGMAKDG	33.96
1059	Variable large protein	O06878	DRB1*13:11	161	175	KASVKGIAKGIKEIV	36.44
1060	Variable large protein	O06878	DRB1*13:11	122	136	VSELLDKLVKAVKTA	45.01
1061	Variable large protein	O06878	DRB1*13:11	125	139	LLDKLVKAVKTAEGA	48.95
1062	Variable large protein	O06878	DRB1*14:32	12	26	TFFVFINCKSQVADK	48.75
1063	Variable large protein	O06878	DRB1*15:01	67	81	KSDVKTYFTTVAAKL	31.59
1064	Variable large protein	O06878	DRB1*15:01	68	82	SDVKTYFTTVAAKLE	46.72
1065	Variable large protein	O06878	DRB1*15:01	66	80	KKSDVKTYFTTVAAK	49.06
1066	Variable large protein	O06878	DRB1*15:02	45	59	LDVFTSFGGLVAEAF	46.6
1067	Variable large protein	O06878	DRB1*15:06	67	81	KSDVKTYFTTVAAKL	31.59
1068	Variable large protein	O06878	DRB1*15:06	68	82	SDVKTYFTTVAAKLE	46.72
1069	Variable large protein	O06878	DRB1*15:06	66	80	KKSDVKTYFTTVAAK	49.06
1070	Variable large protein	O06878	DRB1*15:15	46	60	DVFTSFGGLVAEAFG	48.73
1071	Variable large protein	O06878	DRB1*16:01	70	84	VKTYFTTVAAKLEKT	39.36
1072	Variable large protein	O06878	DRB1*16:01	12	26	TFFVFINCKSQVADK	40.59
1073	Variable large protein	O06878	DRB1*16:01	69	83	DVKTYFTTVAAKLEK	40.97
1074	Variable large protein	O06878	DRB1*16:01	71	85	KTYFTTVAAKLEKTK	49.57
1075	Variable large protein	O06878	DRB1*16:02	69	83	DVKTYFTTVAAKLEK	21.52
1076	Variable large protein	O06878	DRB1*16:02	70	84	VKTYFTTVAAKLEKT	22.13
1077	Variable large protein	O06878	DRB1*16:02	68	82	SDVKTYFTTVAAKLE	26.43
1078	Variable large protein	O06878	DRB1*16:02	71	85	KTYFTTVAAKLEKTK	27.04
1079	Variable large protein	O06878	DRB1*16:02	67	81	KSDVKTYFTTVAAKL	31.38
1080	Variable large protein	O06878	DRB1*16:02	12	26	TFFVFINCKSQVADK	44.7
1081	Variable large protein	O06878	DRB1*16:02	44	58	FLDVFTSFGGLVAEA	48.36
1082	Variable large protein	O06878	DRB1*16:02	45	59	LDVFTSFGGLVAEAF	48.66
1083	Variable large protein	O06878	DRB1*16:05	70	84	VKTYFTTVAAKLEKT	47.7
1084	Variable large protein	O06878	DRB1*16:05	69	83	DVKTYFTTVAAKLEK	47.87
1085	Variable large protein	O06878	DRB1*16:09	70	84	VKTYFTTVAAKLEKT	39.27
1086	Variable large protein	O06878	DRB1*16:09	69	83	DVKTYFTTVAAKLEK	40.76
1087	Variable large protein	O06878	DRB1*16:09	12	26	TFFVFINCKSQVADK	46.99

Table S4. Cross-tabulation of counts of occurrences of strong binders between alleles and *B. burgdorferi* proteins. N denotes the number of proteins for which an allele showed strong binding. Total denotes the number of strong peptide binders for the associated allele and protein.

				IC_{50}			Protein				
Index	Allele	N	Protein	Mean	Strongest	Total	O50917	Q6RH12	P0CL66	Q07337	O06878
							Decorin-binding protein A	OppA-2	Outer surface protein A	Outer surface protein C	Variable large protein
1	DPB1*15:01	1	O50917	44.91	41.54	3	3				
2	DPB1*33:01	3	O06878	40.78	32.11	7	9			3	7
3			O50917	41.29	30.36	9					
4			Q07337	38.36	35.20	3					
5	DPB1*71:01	3	O06878	40.78	32.11	7	9			3	7
6			O50917	41.29	30.36	9					
7			Q07337	38.36	35.20	3					
8	DRB1*01:01	5	O06878	25.77	6.41	50	14	9	21	18	50
9			O50917	36.81	13.72	14					
10			P0CL66	29.88	11.08	21					
11			Q07337	37.67	21.56	18					
12			Q6RH12	35.77	20.50	9					
13	DRB1*01:02	4	O06878	35.77	22.12	11	3		1	1	11
14			O50917	40.09	32.54	3					
15			P0CbL66	46.22	46.22	1					
16			Q07337	45.25	45.25	1					
17	DRB1*01:11	4	O06878	31.31	13.24	12	2		5	1	12
18			O50917	42.73	35.56	2					
19			P0CL66	36.18	26.28	5					
20			Q07337	47.46	47.46	1					
21	DRB1*01:18	5	O06878	24.10	6.06	51	19	12	23	21	51
22			O50917	33.49	12.48	19					
23			P0CL66	33.38	14.30	23					
24			Q07337	38.01	22.02	21					
25			Q6RH12	31.74	17.27	12					
26	DRB1*01:20	5	O06878	28.35	8.36	46	14	8	20	22	46
27			O50917	29.70	10.97	14					
28			P0CL66	35.67	18.39	20					
29			Q07337	36.04	16.27	22					
30			Q6RH12	41.18	32.39	8					
31	DRB1*01:24	5	O06878	33.20	12.41	23	3	1	5	1	23
32			O50917	44.07	34.17	3					
33			P0CL66	31.70	25.34	5					

34			Q07337	49.91	49.91	1					
35			Q6RH12	46.64	46.64	1					
36	DRB1*01:29	5	O06878	29.92	12.01	26	3	1	5	3	26
37			O50917	44.27	35.39	3					
38			P0CL66	38.81	28.58	5					
39			Q07337	45.57	38.90	3					
40			Q6RH12	46.78	46.78	1					
41	DRB1*03:01	2	O06878	45.47	42.11	2	2				2
42			O50917	41.68	35.89	2					
43	DRB1*03:04	2	O06878	45.47	42.11	2	2				2
44			O50917	41.68	35.89	2					
45	DRB1*03:11	4	O06878	28.50	20.51	5	5	1	3		5
46			O50917	31.98	22.69	5					
47			P0CL66	43.92	40.39	3					
48			Q6RH12	45.93	45.93	1					
49	DRB1*03:13	2	O06878	45.47	42.11	2	2				2
50			O50917	41.68	35.89	2					
51	DRB1*03:15	1	O50917	48.49	48.49	1	1				
52	DRB1*04:01	3	O06878	38.02	35.76	2			6	1	2
53			P0CL66	25.83	17.83	6					
54			Q07337	49.37	49.37	1					
55	DRB1*04:04	2	O06878	37.13	30.46	5				1	5
56			Q07337	49.63	49.63	1					
57	DRB1*04:05	2	O50917	41.45	41.04	2	2	2			
58			Q6RH12	48.21	47.57	2					
59	DRB1*04:08	3	O06878	37.88	29.89	4			6	1	4
60			P0CL66	33.14	22.86	6					
61			Q07337	47.57	47.57	1					
62	DRB1*04:10	1	O50917	39.26	38.91	2	2				
63	DRB1*04:72	1	P0CL66	33.44	28.45	4			4		
64	DRB1*07:01	4	O06878	14.43	7.02	7	1	3		6	7
65			O50917	45.05	45.05	1					
66			Q07337	25.30	16.69	6					
67			Q6RH12	38.06	33.98	3					
68	DRB1*08:04	2	O06878	37.26	32.53	3	2				3
69			O50917	45.09	42.28	2					
70	DRB1*08:24	1	O50917	47.45	42.84	3	3				
71	DRB1*09:01	2	O06878	33.17	23.87	10		4			10
72			Q6RH12	45.29	40.30	4					
73	DRB1*10:01	5	O06878	27.89	12.16	35	3	5	6	6	35
74			O50917	39.21	34.85	3					
75			P0CL66	30.05	19.36	6					
76			Q07337	45.05	41.09	6					

77			Q6RH12	37.47	29.56	5					
78	DRB1*11:01	1	O50917	31.00	18.15	9	9				
79	DRB1*11:02	1	O50917	39.01	32.21	7	7				
80	DRB1*11:03	3	O06878	41.28	41.28	1	13		1		1
81			O50917	34.68	18.96	13					
82			P0CL66	44.48	44.48	1					
83	DRB1*11:04	3	O06878	36.53	26.14	6	17			1	6
84			O50917	33.68	18.32	17					
85			Q07337	47.59	47.59	1					
86	DRB1*11:08	1	O50917	35.84	26.24	6	6				
87	DRB1*11:10	1	O50917	31.00	18.15	9	9				
88	DRB1*11:12	1	O50917	31.00	18.15	9	9				
89	DRB1*11:13	3	O06878	47.74	46.09	2	10			8	2
90			O50917	37.65	26.81	10					
91			Q07337	41.28	31.98	8					
92	DRB1*11:14	1	Q07337	27.54	15.22	6				6	
93	DRB1*11:27	1	O50917	39.22	36.08	3	3				
94	DRB1*11:28	1	O50917	31.00	18.15	9	9				
95	DRB1*11:29	1	O50917	31.00	18.15	9	9				
96	DRB1*11:37	1	O50917	38.50	35.70	3	3				
97	DRB1*11:42	4	O06878	34.86	20.58	13	20	1		11	13
98			O50917	30.57	16.33	20					
99			Q07337	36.38	29.06	11					
100			Q6RH12	46.47	46.47	1					
101	DRB1*11:46	3	O06878	36.53	26.14	6	17			1	6
102			O50917	33.68	18.32	17					
103			Q07337	47.59	47.59	1					
104	DRB1*11:49	1	O50917	31.00	18.15	9	9				
105	DRB1*11:58	3	O06878	36.53	26.14	6	17			1	6
106			O50917	33.68	18.32	17					
107			Q07337	47.59	47.59	1					
108	DRB1*11:62	1	O50917	31.00	18.15	9	9				
109	DRB1*11:65	1	O50917	39.01	32.21	7	7				
110	DRB1*11:74	1	O50917	31.00	18.15	9	9				
111	DRB1*13:01	1	O50917	39.01	32.21	7	7				
112	DRB1*13:02	1	Q07337	27.54	15.22	6				6	
113	DRB1*13:05	1	O50917	31.00	18.15	9	9				
114	DRB1*13:07	1	O50917	38.50	35.70	3	3				
115	DRB1*13:11	3	O06878	36.53	26.14	6	17			1	6
116			O50917	33.68	18.32	17					
117			Q07337	47.59	47.59	1					
118	DRB1*13:14	1	O50917	31.00	18.15	9	9				
119	DRB1*13:21	1	O50917	39.62	30.58	12	12				

120	DRB1*13:23	1	Q07337	27.54	15.22	6				6	
121	DRB1*13:50	1	O50917	31.00	18.15	9	9				
122	DRB1*13:96	1	Q07337	35.52	30.67	3				3	
123	DRB1*13:97	1	Q07337	27.54	15.22	6				6	
124	DRB1*14:32	4	O06878	48.75	48.75	1	2		3	7	1
125			O50917	48.62	47.78	2					
126			P0CL66	41.26	37.71	3					
127			Q07337	38.01	30.20	7					
128	DRB1*15:01	2	O06878	42.46	31.59	3		9			3
129			Q6RH12	24.13	9.61	9					
130	DRB1*15:02	2	O06878	46.60	46.60	1		5			1
131			Q6RH12	23.49	18.25	5					
132	DRB1*15:03	1	Q6RH12	25.03	20.09	5		5			
133	DRB1*15:06	2	O06878	42.46	31.59	3					3
134			Q6RH12	24.13	9.61	9		9			
135	DRB1*15:07	1	Q6RH12	20.88	14.43	6		6			
136	DRB1*15:15	2	O06878	48.73	48.73	1					1
137			Q6RH12	32.89	27.08	4		4			
138	DRB1*15:37	1	Q6RH12	19.76	16.36	5		5			
139	DRB1*16:01	1	O06878	42.62	39.36	4					4
140	DRB1*16:02	2	O06878	33.78	21.52	8					8
141			Q6RH12	41.27	35.71	3		3			
142	DRB1*16:05	1	O06878	47.79	47.70	2					2
143	DRB1*16:09	1	O06878	42.34	39.27	3					3

Table S5. The 226 unique (distinct) peptides with strong binding affinities. N is the number of occurrences. The 8-mers in color denote subsequences that were found in 2/83607 (0.24%) human proteins tested. See text for details.

			IC_{50}	
Index	Peptide	N	Mean	Strongest
1	NPFILEAKVRATTVA	31	28.43	18.37
2	ENPFILEAKVRATTV	28	27.11	18.15
3	SENPFILEAKVRATT	24	30.85	21.33
4	PFILEAKVRATTVAE	21	32.18	29.02
5	VSENPFILEAKVRAT	18	32.09	24.51
6	DDQIAAAIALRGMAK	16	26.97	9.38
7	DQIAAAIALRGMAKD	16	25.65	8.36
8	DVKTYFTTVAAKLEK	15	23.21	6.18
9	IAPIYIYGNSYLFRN	15	26.25	9.61
10	NKTFNNLLKLTILVN	15	37.24	32.88
11	VKTYFTTVAAKLEKT	15	23.33	6.06
12	KSDVKTYFTTVAAKL	14	25.97	7.02
13	SDVKTYFTTVAAKLE	14	25.02	7.93
14	APIYIYGNSYLFRND	13	26.25	9.74
15	CNNKTFNNLLKLTIL	13	43.80	43.30
16	MYDLMFEVSKPLQKL	13	33.14	10.97
17	PIAPIYIYGNSYLFR	13	26.84	10.04
18	QIAAAIALRGMAKDG	13	27.85	10.24
19	SKKITDSNAVLLAVK	13	27.63	15.22
20	TFFVFINCKSQVADK	13	31.89	11.91
21	NNKTFNNLLKLTILV	12	42.75	40.77
22	AQGVLEIAKKMREKL	11	25.26	16.33
23	GVLEIAKKMREKLQR	11	25.22	18.76
24	GVSENPFILEAKVRA	11	46.31	46.31
25	KTYFTTVAAKLEKTK	11	19.88	6.80
26	QGVLEIAKKMREKLQ	11	24.11	16.37
27	SCGLTGATKIRLERS	11	44.11	29.86
28	VLEIAKKMREKLQRV	11	30.89	22.79
29	YDLMFEVSKPLQKLG	11	36.63	14.50
30	ESAVRKVLGAITGLI	10	29.08	11.69
31	KGYVLEGTLTAEKTT	10	31.76	14.35
32	LKGYVLEGTLTAEKT	10	23.25	11.16
33	PIYIYGNSYLFRNDK	10	25.90	10.86
34	VLKGYVLEGTLTAEK	10	23.27	11.08
35	FFVFINCKSQVADKD	9	29.16	12.61
36	ISKKITDSNAVLLAV	9	23.42	16.78
37	KKITDSNAVLLAVKE	9	23.77	18.79
38	SAVRKVLGAITGLIG	9	29.15	13.42

39	TYFTTVAAKLEKTKT	9	25.40	10.49
40	AVRKVLGAITGLIGD	8	31.51	14.44
41	EVLKGYVLEGTLTAE	8	32.51	18.53
42	KDDQIAAAIALRGMA	8	25.97	11.49
43	LDVFTSFGGLVAEAF	8	34.44	13.26
44	TAQGVLEIAKKMREK	8	34.37	23.66
45	VRKVLGAITGLIGDA	8	32.14	13.07
46	AMYDLMFEVSKPLQK	7	33.84	13.37
47	DVFTSFGGLVAEAFG	7	32.79	14.02
48	RKVLGAITGLIGDAV	7	29.21	12.47
49	AESAVRKVLGAITGL	6	28.45	15.15
50	EISKKITDSNAVLLA	6	26.96	20.14
51	FESVEVLSKAAKEML	6	44.75	29.06
52	FLDVFTSFGGLVAEA	6	34.84	15.84
53	FPIAPIYIYGNSYLF	6	24.76	14.51
54	GEQILSAIVTAADAA	6	30.24	22.14
55	KASVKGIAKGIKEIV	6	36.52	32.06
56	KITDSNAVLLAVKEV	6	42.68	40.01
57	KKDDQIAAAIALRGM	6	30.51	15.42
58	KLGIQEMTKTVSDAA	6	33.84	28.43
59	TTFFVFINCKSQVAD	6	32.44	18.77
60	VFTSFGGLVAEAFGF	6	32.75	16.30
61	EQILSAIVTAADAAE	5	32.68	24.66
62	GSLLAGAYAISTLIK	5	31.65	23.21
63	GYVLEGTLTAEKTTL	5	37.51	28.18
64	IDAIKKDAALKGVNF	5	35.77	22.69
65	KEVLKGYVLEGTLTA	5	41.97	34.75
66	LAGAYAISTLIKQKL	5	42.93	36.18
67	LGIQEMTKTVSDAAE	5	46.55	40.72
68	LLDK**LVKAVKTA**EGA	5	46.83	38.34
69	LQKLGIQEMTKTVSD	5	48.15	43.82
70	MKKYLLGIGLILALI	5	33.12	21.08
71	MTLFLFISCNNSGKD	5	45.50	41.49
72	QKLGIQEMTKTVSDA	5	35.82	32.91
73	QSVIQLGNGFLDVFT	5	40.50	23.66
74	SAMYDLMFEVSKPLQ	5	33.04	23.28
75	SGEQILSAIVTAADA	5	33.01	28.16
76	TEISKKITDSNAVLL	5	43.56	28.46
77	VSELLDK**LVKAVKTA**	5	42.38	31.84
78	AEEIIIEKDFPIAPI	4	40.38	32.39
79	ALRGMAKDGKFAVKD	4	42.10	21.92
80	DPLTFLSIFTQGYTQ	4	36.16	27.25
81	EEIIIEKDFPIAPIY	4	45.93	36.15
82	EIDAIKKDAALKGVN	4	42.26	26.67

83	EKIITRADGTRLEYT	4	29.10	18.39
84	FTSFGGLVAEAFGFK	4	29.39	20.72
85	FVFINCKSQVADKDD	4	34.63	20.04
86	IALRGMAKDGKFAVK	4	36.71	20.51
87	ISCGLTGATKIRLER	4	41.99	34.80
88	KFYQSVIQLGNGFLD	4	31.99	23.50
89	KKYLLGIGLILALIA	4	32.43	23.79
90	LEIAKKMREKLQRVH	4	44.46	31.15
91	NGSLLAGAYAISTLI	4	36.56	31.12
92	PLTFLSIFTQGYTQF	4	34.84	25.28
93	TLFLFISCNNSGKDG	4	48.44	45.41
94	VSGEQILSAIVTAAD	4	44.48	41.32
95	AAESAVRKVLGAITG	3	24.67	23.51
96	ADPLTFLSIFTQGYT	3	39.91	32.85
97	AEKFVIAIEEEATKL	3	38.20	34.85
98	AGAYAISTLIKQKLD	3	33.67	31.95
99	ANSVKELTSPVVAES	3	31.92	30.24
100	AYAISTLIKQKLDGL	3	31.10	30.20
101	DFPIAPIYIYGNSYL	3	27.14	22.43
102	DLMFEVSKPLQKLGI	3	27.31	23.92
103	EFSAMYDLMFEVSKP	3	34.09	30.36
104	EGTLTAEKTTLVVKE	3	37.17	30.55
105	EKFVIAIEEEATKLK	3	43.28	38.82
106	EKLKAVAAAKGENNK	3	34.29	32.42
107	ESVEVLSKAAKEMLA	3	42.48	33.00
108	FSAMYDLMFEVSKPL	3	35.24	30.47
109	FYQSVIQLGNGFLDV	3	30.08	21.06
110	GAYAISTLIKQKLDG	3	35.31	35.04
111	GEFSAMYDLMFEVSK	3	39.11	34.46
112	GGSEKLKAVAAAKGE	3	33.58	32.18
113	GSEKLKAVAAAKGEN	3	27.79	27.01
114	GTLTAEKTTLVVKEG	3	39.58	32.48
115	HNGSLLAGAYAISTL	3	30.91	29.45
116	IYIYGNSYLFRNDKW	3	46.67	44.91
117	KIITRADGTRLEYTG	3	26.55	20.04
118	KQKLDGLKNEGLKEK	3	39.37	35.06
119	KTTLVVKEGTVTLSK	3	43.46	37.24
120	KVLGAITGLIGDAVS	3	22.54	16.89
121	LANSVKELTSPVVAE	3	36.55	33.88
122	LEGTLTAEKTTLVVK	3	33.27	26.85
123	LGAITGLIGDAVSSG	3	32.68	26.36
124	LLAGAYAISTLIKQK	3	40.45	39.02
125	LTFLSIFTQGYTQFS	3	42.03	33.75
126	LVNLLISCGLTGATK	3	40.29	34.31

127	MKKDDQIAAAIALRG	3	43.33	36.41
128	MLANSVKELTSPVVA	3	40.37	36.15
129	NHNGSLLAGAYAIST	3	36.44	35.55
130	NKFYQSVIQLGNGFL	3	31.26	16.95
131	NLLISCGLTGATKIR	3	43.29	37.89
132	NSVKELTSPVVAESP	3	40.50	36.50
133	SEKIITRADGTRLEY	3	26.05	19.64
134	SEKLKAVAAAKGENN	3	27.88	26.08
135	SLLAGAYAISTLIKQ	3	32.86	32.35
136	SVEVLSKAAKEMLAN	3	44.77	35.87
137	SVIQLGNGFLDVFTS	3	46.12	45.20
138	TFLSIFTQGYTQFSS	3	44.47	40.58
139	TNKFYQSVIQLGNGF	3	32.12	16.23
140	TTLVVKEGTVTLSKN	3	44.65	37.17
141	VLGAITGLIGDAVSS	3	28.12	22.50
142	VNLLISCGLTGATKI	3	40.75	34.01
143	YAISTLIKQKLDGLK	3	35.59	35.36
144	YFTTVAAKLEKTKTD	3	43.43	40.93
145	YQSVIQLGNGFLDVF	3	27.20	18.80
146	AALKGVNFDAFKDKK	2	43.42	43.42
147	AEEL**GKLFESVE**VLS	2	35.20	35.20
148	CGLTGATKIRLERSA	2	43.82	39.63
149	DAALKGVNFDAFKDK	2	47.85	47.85
150	DLVFTKENTITVQQY	2	30.00	26.81
151	EEL**GKLFESVE**VLSK	2	43.24	43.24
152	GAEEL**GKLFESVE**VL	2	36.63	36.63
153	GATKIRLERSAKDIT	2	39.98	38.91
154	GFLDVFTSFGGLVAE	2	26.22	22.80
155	GIGLILALIACKQNV	2	36.11	32.54
156	**GKLFESVE**VLSKAAK	2	45.05	41.09
157	GLILALIACKQNVSS	2	33.85	32.55
158	IGLILALIACKQNVS	2	36.75	36.09
159	KDLVFTKENTITVQQ	2	38.74	35.65
160	KKDAALKGVNFDAFK	2	47.74	47.74
161	KKISSAILLTTFFVF	2	49.73	49.73
162	KKSDVKTYFTTVAAK	2	49.06	49.06
163	KLFESVEVLSKAAKE	2	43.21	42.08
164	KVRATTVAEKFVIAI	2	45.48	45.48
165	LGIGLILALIACKQN	2	38.11	35.58
166	LLISCGLTGATKIRL	2	44.96	44.86
167	LSSIDEIAAKAIGKK	2	44.79	43.08
168	LTTFFVFINCKSQVA	2	41.25	36.23
169	LVFTKENTITVQQYD	2	29.94	26.93
170	NNHNGSLLAGAYAIS	2	46.90	46.12

171	QILSAIVTAADAAEQ	2	41.48	35.54
172	SKAAKEMLANSVKEL	2	48.66	48.05
173	TGATKIRLERSAKDI	2	40.73	39.61
174	TSFGGLVAEAFGFKS	2	41.97	39.26
175	VRATTVAEKFVIAIE	2	49.54	49.54
176	VSEKIITRADGTRLE	2	37.38	30.21
177	AIALRGMAKDGKFAV	1	40.75	40.75
178	AIKKDAALKGVNFDA	1	48.57	48.57
179	ASVKGIAKGIKEIVE	1	42.29	42.29
180	AVLLAVKEVEALLSS	1	49.88	49.88
181	AVSGEQILSAIVTAA	1	48.82	48.82
182	DAIKKDAALKGVNFD	1	31.87	31.87
183	DEIDAIKKDAALKGV	1	30.12	30.12
184	DKASVKGIAKGIKEI	1	46.67	46.67
185	DPTNKFYQSVIQLGN	1	25.36	25.36
186	EIAKKMREKLQRVHT	1	45.34	45.34
187	EIIIEKDFPIAPIYI	1	46.88	46.88
188	ELLDK**LVKAVKTA**EG	1	38.04	38.04
189	EVSELLDKLVKAVKT	1	37.76	37.76
190	FILEAKVRATTVAEK	1	46.35	46.35
191	GAAESAVRKVLGAIT	1	47.35	47.35
192	GAITGLIGDAVSSGL	1	39.51	39.51
193	GKYDLIATVDKLELK	1	37.71	37.71
194	IAAAIALRGMAKDGK	1	37.63	37.63
195	IITRADGTRLEYTGI	1	47.85	47.85
196	IKQKLDGLKNEGLKE	1	47.61	47.61
197	KCNNKTFNNLLKLTI	1	41.55	41.55
198	KEAILKTNGTKTKGA	1	49.13	49.13
199	KEVSELLDKLVKAVK	1	46.66	46.66
200	KLKAVAAAKGENNKG	1	41.66	41.66
201	KSDLELDPIKRQDIL	1	45.93	45.93
202	KTFNNLLKLTILVNL	1	36.25	36.25
203	KYDLIATVDKLELKG	1	41.91	41.91
204	LELDPIKRQDILRQA	1	46.47	46.47
205	LEVFKEDGKTLVSKK	1	43.53	43.53
206	LFESVEVLSKAAKEM	1	37.57	37.57
207	L**GKLFESVE**VLSKAA	1	45.54	45.54
208	LIKQKLDGLKNEGLK	1	49.71	49.71
209	LILALIACKQNVSSL	1	47.94	47.94
210	LMFEVSKPLQKLGIQ	1	45.37	45.37
211	LMTLFLFISCNNSGK	1	49.44	49.44
212	LRGMAKDGKFAVKDG	1	24.40	24.40
213	NGFLDVFTSFGGLVA	1	42.46	42.46
214	PTNKFYQSVIQLGNG	1	20.71	20.71

215	QAEEIIIEKDFPIAP	1	48.34	48.34
216	RGMAKDGKFAVKDGE	1	34.90	34.90
217	RQAEEIIIEKDFPIA	1	44.61	44.61
218	SELLDK**LVKAVKTA**E	1	36.00	36.00
219	TKDLVFTKENTITVQ	1	48.81	48.81
220	TLEVFKEDGKTLVSK	1	47.85	47.85
221	TLTITVNSKKTKDLV	1	44.48	44.48
222	TLVVKEGTVTLSKNI	1	45.87	45.87
223	TTAQGVLEIAKKMRE	1	37.64	37.64
224	VFINCKSQVADKDDP	1	47.04	47.04
225	YDLIATVDKLELKGT	1	44.17	44.17
226	YTGIKSDGSGKAKEV	1	40.39	40.39

Table S6. The 142 HLA Class I alleles used to estimate predicted binding affinities of GKLFESVE, LVKAVKTA 8-mers. See text for details.

	Gene A		Gene B		Gene C
1	A*01:01	1	B*07:02	1	C*01:02
2	A*02:01	2	B*07:04	2	C*02:02
3	A*02:02	3	B*07:05	3	C*02:10
4	A*02:05	4	B*08:01	4	C*03:02
5	A*02:06	5	B*13:02	5	C*03:03
6	A*02:17	6	B*14:01	6	C*03:04
7	A*02:30	7	B*14:02	7	C*04:01
8	A*02:35	8	B*14:03	8	C*05:01
9	A*02:63	9	B*15:01	9	C*06:02
10	A*02:77	10	B*15:03	10	C*07:01
11	A*03:01	11	B*15:07	11	C*07:02
12	A*03:02	12	B*15:09	12	C*07:04
13	A*03:81	13	B*15:10	13	C*07:19
14	A*11:01	14	B*15:16	14	C*08:01
15	A*11:02	15	B*15:17	15	C*08:02
16	A*23:01	16	B*15:18	16	C*08:03
17	A*24:02	17	B*15:24	17	C*12:02
18	A*24:03	18	B*15:35	18	C*12:03
19	A*25:01	19	B*18:01	19	C*14:02
20	A*26:01	20	B*18:09	20	C*15:02
21	A*26:08	21	B*27:02	21	C*15:04
22	A*26:12	22	B*27:04	22	C*15:05
23	A*29:01	23	B*27:05	23	C*15:06
24	A*29:02	24	B*27:07	24	C*15:09
25	A*30:01	25	B*27:08	25	C*16:01
26	A*30:02	26	B*27:10	26	C*16:02
27	A*30:04	27	B*35:01	27	C*16:04
28	A*31:01	28	B*35:02	28	C*17:01
29	A*32:01	29	B*35:03	29	C*18:01
30	A*33:01	30	B*35:08		
31	A*33:03	31	B*35:17		
32	A*34:01	32	B*37:01		
33	A*34:02	33	B*38:01		
34	A*36:01	34	B*39:01		
35	A*66:01	35	B*39:02		
36	A*68:01	36	B*39:05		
37	A*68:02	37	B*39:06		
38	A*68:37	38	B*39:24		
39	A*74:01	39	B*40:01		
40	A*74:03	40	B*40:02		
41	A*80:01	41	B*41:01		

	42	B*41:02	
	43	B*42:02	
	44	B*44:02	
	45	B*44:03	
	46	B*44:04	
	47	B*44:05	
	48	B*44:07	
	49	B*44:27	
	50	B*45:01	
	51	B*47:01	
	52	B*48:01	
	53	B*48:07	
	54	B*49:01	
	55	B*50:01	
	56	B*50:02	
	57	B*51:01	
	58	B*51:02	
	59	B*51:07	
	60	B*51:09	
	61	B*52:01	
	62	B*53:01	
	63	B*54:01	
	64	B*55:01	
	65	B*56:01	
	66	B*57:01	
	67	B*57:02	
	68	B*57:03	
	69	B*58:01	
	70	B*58:02	
	71	B*59:01	
	72	B*81:01	

Table S7. Number of 15-mer peptides with moderate predicted binding affinity to *B. burgdorferi* proteins (50 nM ≤ IC_{50} < 500 nM).

Index	Allele	N
1	DPB1*01:01	148
2	DPB1*02:01	117
3	DPB1*02:02	136
4	DPB1*03:01	19
5	DPB1*04:01	71
6	DPB1*04:02	7
7	DPB1*05:01	31
8	DPB1*06:01	11
9	DPB1*09:01	8
10	DPB1*104:01	19
11	DPB1*105:01	7
12	DPB1*11:01	19
13	DPB1*124:01	19
14	DPB1*126:01	71
15	DPB1*13:01	11
16	DPB1*14:01	21
17	DPB1*15:01	150
18	DPB1*16:01	67
19	DPB1*17:01	4
20	DPB1*19:01	59
21	DPB1*20:01	20
22	DPB1*23:01	71
23	DPB1*26:01	10
24	DPB1*28:01	14
25	DPB1*30:01	5
26	DPB1*33:01	207
27	DPB1*34:01	72
28	DPB1*35:01	7
29	DPB1*39:01	71
30	DPB1*40:01	42
31	DPB1*41:01	54
32	DPB1*46:01	117
33	DPB1*47:01	136
34	DPB1*49:01	7
35	DPB1*55:01	28
36	DPB1*71:01	207
37	DPB1*72:01	107
38	DPB1*81:01	117
39	DPB1*85:01	13
40	DPB1*91:01	20

41	DQB1*03:01	25
42	DQB1*03:10	25
43	DQB1*03:19	25
44	DQB1*05:01	35
45	DQB1*05:03	8
46	DQB1*05:06	8
47	DQB1*05:11	35
48	DQB1*06:01	54
49	DQB1*06:02	8
50	DQB1*06:08	6
51	DQB1*06:09	1
52	DQB1*06:11	12
53	DQB1*06:18	3
54	DQB1*06:19	8
55	DRB1*01:01	354
56	DRB1*01:02	290
57	DRB1*01:03	34
58	DRB1*01:11	260
59	DRB1*01:18	386
60	DRB1*01:20	420
61	DRB1*01:24	248
62	DRB1*01:29	306
63	DRB1*03:01	74
64	DRB1*03:04	74
65	DRB1*03:05	33
66	DRB1*03:11	229
67	DRB1*03:13	74
68	DRB1*03:15	146
69	DRB1*03:41	6
70	DRB1*04:01	171
71	DRB1*04:02	43
72	DRB1*04:03	26
73	DRB1*04:04	229
74	DRB1*04:05	147
75	DRB1*04:06	26
76	DRB1*04:07	37
77	DRB1*04:08	173
78	DRB1*04:10	191
79	DRB1*04:11	9
80	DRB1*04:17	19
81	DRB1*04:44	138
82	DRB1*04:53	30
83	DRB1*04:56	118
84	DRB1*04:72	98

85	DRB1*07:01	222
86	DRB1*08:01	124
87	DRB1*08:02	112
88	DRB1*08:03	108
89	DRB1*08:04	235
90	DRB1*08:24	111
91	DRB1*08:30	117
92	DRB1*08:36	108
93	DRB1*09:01	214
94	DRB1*09:02	108
95	DRB1*10:01	333
96	DRB1*11:01	174
97	DRB1*11:02	275
98	DRB1*11:03	288
99	DRB1*11:04	266
100	DRB1*11:06	88
101	DRB1*11:07	51
102	DRB1*11:08	253
103	DRB1*11:10	174
104	DRB1*11:11	130
105	DRB1*11:12	174
106	DRB1*11:13	440
107	DRB1*11:14	154
108	DRB1*11:19	96
109	DRB1*11:27	47
110	DRB1*11:28	174
111	DRB1*11:29	174
112	DRB1*11:37	92
113	DRB1*11:42	383
114	DRB1*11:46	266
115	DRB1*11:49	174
116	DRB1*11:54	63
117	DRB1*11:58	266
118	DRB1*11:62	174
119	DRB1*11:65	275
120	DRB1*11:74	174
121	DRB1*11:84	134
122	DRB1*12:01	51
123	DRB1*12:02	93
124	DRB1*12:03	205
125	DRB1*12:16	199
126	DRB1*13:01	275
127	DRB1*13:02	154
128	DRB1*13:03	70

129	DRB1*13:05	174
130	DRB1*13:07	92
131	DRB1*13:11	266
132	DRB1*13:12	70
133	DRB1*13:14	174
134	DRB1*13:21	198
135	DRB1*13:23	154
136	DRB1*13:26	12
137	DRB1*13:33	23
138	DRB1*13:50	174
139	DRB1*13:61	111
140	DRB1*13:66	132
141	DRB1*13:96	111
142	DRB1*13:97	154
143	DRB1*14:01	172
144	DRB1*14:02	139
145	DRB1*14:03	47
146	DRB1*14:04	177
147	DRB1*14:05	80
148	DRB1*14:06	309
149	DRB1*14:07	75
150	DRB1*14:12	171
151	DRB1*14:23	80
152	DRB1*14:27	8
153	DRB1*14:32	472
154	DRB1*14:38	76
155	DRB1*14:44	20
156	DRB1*14:54	172
157	DRB1*14:68	79
158	DRB1*15:01	148
159	DRB1*15:02	95
160	DRB1*15:03	214
161	DRB1*15:06	148
162	DRB1*15:07	107
163	DRB1*15:15	135
164	DRB1*15:37	67
165	DRB1*16:01	104
166	DRB1*16:02	173
167	DRB1*16:04	77
168	DRB1*16:05	78
169	DRB1*16:09	124
170	Total	20152

Table S8. The 731 distinct 15-mer peptides with moderate predicted binding affinity to 5 *B. burgdorferi* antigens tested.

Index	Peptide (15-mer)
1	AAAIALRGMAKDGKF
2	AAESAVRKVLGAITG
3	AAGAVSAVSGEQILS
4	AAGGSEKLKAVAAAK
5	AAIALRGMAKDGKFA
6	AAIGEVVADADAAKV
7	AAKAIGKKIHQNNGL
8	AAKEMLANSVKELTS
9	AAKKCSETFTNKLKE
10	AAKVADKASVKGIAK
11	AALKGVNFDAFKDKK
12	AASKAAGAVSAVSGE
13	AAWNSGTSTLTITVN
14	ADAAKVADKASVKGI
15	ADAKEAILKTNGTKT
16	ADKASVKGIAKGIKE
17	ADKSKVKLTISDDLG
18	ADPLTFLSIFTQGYT
19	AEAFGFKSDPKKSDV
20	AEEIIIEKDFPIAPI
21	AEELGKLFESVEVLS
22	AEGAIKGAAESAVRK
23	AEKFVIAIEEEATKL
24	AEKTTLVVKEGTVTL
25	AESAVRKVLGAITGL
26	AFGFKSDPKKSDVKT
27	AGAVSAVSGEQILSA
28	AGAYAISTLIKQKLD
29	AGGSEKLKAVAAAKG
30	AGKLFGKAGAAAHGD
31	AGWIGDYADPLTFLS
32	AIALRGMAKDGKFAV
33	AIGEVVADADAAKVA
34	AIGKKIHQNNGLDTE
35	AIKEVSELLDKLVKA
36	AIKGAAESAVRKVLG
37	AIKKDAALKGVNFDA
38	AILKTNGTKTKGAEE
39	AILLTTFFVFINCKS
40	AILMTLFLFISCNNS

41	AISTLIKQKLDGLKN
42	AITGLIGDAVSSGLR
43	AKEAILKTNGTKTKG
44	AKEMLANSVKELTSP
45	AKEVLKGYVLEGTLT
46	AKKCSETFTNKLKEK
47	AKKMREKLQRVHTKN
48	AKVADKASVKGIAKG
49	AKVRATTVAEKFVIA
50	ALIACKQNVSSLDEK
51	ALKGVNFDAFKDKKT
52	ALLSSIDEIAAKAIG
53	ALRGMAKDGKFAVKD
54	AMYDLMFEVSKPLQK
55	ANSVKELTSPVVAES
56	APIYIYGNSYLFRND
57	AQGVLEIAKKMREKL
58	ASKAAGAVSAVSGEQ
59	ASVKGIAKGIKEIVE
60	ATKIRLERSAKDITD
61	ATTVAEKFVIAIEEE
62	AVEGAIKEVSELLDK
63	AVEITKLDEIKNALK
64	AVKEVEALLSSIDEI
65	AVKTAEGASSGTAAI
66	AVLLAVKEVEALLSS
67	AVRKVLGAITGLIGD
68	AVSAVSGEQILSAIV
69	AVSGEQILSAIVTAA
70	AWNSGTSTLTITVNS
71	AYAISTLIKQKLDGL
72	CGLTGATKIRLERSA
73	CKQNVSSLDEKNSVS
74	CNNKTFNNLLKLTIL
75	CSETFTNKLKEKHTD
76	DAAKKCSETFTNKLK
77	DAAKVADKASVKGIA
78	DAALKGVNFDAFKDK
79	DAIKKDAALKGVNFD
80	DAKEAILKTNGTKTK
81	DDPTNKFYQSVIQLG
82	DDQIAAAIALRGMAK
83	DEIAAKAIGKKIHQN
84	DEIDAIKKDAALKGV

85	DEKNSVSVDLPGEMK
86	DEMKKDDQIAAAIAL
87	DFPIAPIYIYGNSYL
88	DGKTLVSKKVTSKDK
89	DGKYDLIATVDKLEL
90	DGSGKAKEVLKGYVL
91	DGTRLEYTGIKSDGS
92	DILRQAEEIIIEKDF
93	DITDEIDAIKKDAAL
94	DKASVKGIAKGIKEI
95	DKLVKAVKTAEGASS
96	DKNNGSGVLEGVKAD
97	DKSKVKLTISDDLGQ
98	DKWTGWNTNFLERFD
99	DLELDPIKRQDILRQ
100	DLIATVDKLELKGTS
101	DLMFEVSKPLQKLGI
102	DLNSLPKEKSDISST
103	DLPGEMKVLVSKEKN
104	DLVFTKENTITVQQY
105	DPIKRQDILRQAEEI
106	DPKKSDVKTYFTTVA
107	DPLTFLSIFTQGYTQ
108	DPTNKFYQSVIQLGN
109	DQIAAAIALRGMAKD
110	DSEAASKAAGAVSAV
111	DSNAVLLAVKEVEAL
112	DSNGTKLEGSAVEIT
113	DSTGSVGTAVEGAIK
114	DTENNHNGSLLAGAY
115	DVFTSFGGLVAEAFG
116	DVKTYFTTVAAKLEK
117	DYADPLTFLSIFTQG
118	EAAGGSEKLKAVAAA
119	EAASKAAGAVSAVSG
120	EAFGFKSDPKKSDVK
121	EAILKTNGTKTKGAE
122	EAKVRATTVAEKFVI
123	EALLSSIDEIAAKAI
124	EDGKTLVSKKVTSKD
125	EEIIIEKDFPIAPIY
126	EELGKLFESVEVLSK
127	EFGQDEMKKDDQIAA
128	EFSAMYDLMFEVSKP

129	EGAIKEVSELLDKLV
130	EGAIKGAAESAVRKV
131	EGSAVEITKLDEIKN
132	EGTLTAEKTTLVVKE
133	EGTVTLSKNISKSGE
134	EGVKADKSKVKLTIS
135	EGVTDADAKEAILKT
136	EIAAKAIGKKIHQNN
137	EIAKKMREKLQRVHT
138	EIDAIKKDAALKGVN
139	EIIIEKDFPIAPIYI
140	EISKKITDSNAVLLA
141	EKDFPIAPIYIYGNS
142	EKFVIAIEEEATKLK
143	EKIITRADGTRLEYT
144	EKLKAVAAAKGENNK
145	EKLQRVHTKNYCTLK
146	EKNSVSVDLPGEMKV
147	EKTKTDLNSLPKEKS
148	EKTTLVVKEGTVTLS
149	ELDPIKRQDILRQAE
150	ELGKLFESVEVLSKA
151	ELIKKSDLELDPIKR
152	ELLDKLVKAVKTAEG
153	EMKKDDQIAAAIALR
154	EMKVLVSKEKNKDGK
155	EMLANSVKELTSPVV
156	ENNHNGSLLAGAYAI
157	ENPFILEAKVRATTV
158	ENTITVQQYDSNGTK
159	EQILSAIVTAADAAE
160	ESAVRKVLGAITGLI
161	ESVEVLSKAAKEMLA
162	ETFTNKLKEKHTDLG
163	EVEALLSSIDEIAAK
164	EVFKEDGKTLVSKKV
165	EVLKGYVLEGTLTAE
166	EVLSKAAKEMLANSV
167	EVSEKIITRADGTRL
168	EVSELLDKLVKAVKT
169	EVSKPLQKLGIQEMT
170	EVVADADAAKVADKA
171	EYNELIKKSDLELDP
172	EYTGIKSDGSGKAKE

173	FESVEVLSKAAKEML
174	FEVSKPLQKLGIQEM
175	FFVFINCKSQVADKD
176	FGFKSDPKKSDVKTY
177	FGGLVAEAFGFKSDP
178	FGQDEMKKDDQIAAA
179	FILEAKVRATTVAEK
180	FINCKSQVADKDDPT
181	FKEDGKTLVSKKVTS
182	FLDVFTSFGGLVAEA
183	FLERFDLCQLKLKNK
184	FLFISCNNSGKDGNT
185	FLSIFTQGYTQFSSH
186	FNNLLKLTILVNLLI
187	FPIAPIYIYGNSYLF
188	FRNDKWTGWNTNFLE
189	FSAMYDLMFEVSKPL
190	FTQGYTQFSSHNYSN
191	FTSFGGLVAEAFGFK
192	FTTVAAKLEKTKTDL
193	FVFINCKSQVADKDD
194	FVIAIEEEATKLKET
195	FYQSVIQLGNGFLDV
196	GAAESAVRKVLGAIT
197	GAEELGKLFESVEVL
198	GAGKLFGKAGAAAHG
199	GAIKEVSELLDKLVK
200	GAIKGAAESAVRKVL
201	GAITGLIGDAVSSGL
202	GATKIRLERSAKDIT
203	GAVSAVSGEQILSAI
204	GAYAISTLIKQKLDG
205	GDSEAASKAAGAVSA
206	GDYADPLTFLSIFTQ
207	GEFSAMYDLMFEVSK
208	GEMKVLVSKEKNKDG
209	GEQILSAIVTAADAA
210	GEVSEKIITRADGTR
211	GEVVADADAAKVADK
212	GFKSDPKKSDVKTYF
213	GFLDVFTSFGGLVAE
214	GGLVAEAFGFKSDPK
215	GGSEKLKAVAAAKGE
216	GIAKGIKEIVEAAGG

217	GIGLILALIACKQNV
218	GIKEIVEAAGGSEKL
219	GIKSDGSGKAKEVLK
220	GIQEMTKTVSDAAEE
221	GKAKEVLKGYVLEGT
222	GKKIHQNNGLDTENN
223	GKLFESVEVLSKAAK
224	GKLFGKAGAAAHGDS
225	GKTLVSKKVTSKDKS
226	GKYDLIATVDKLELK
227	GLIGDAVSSGLRKVG
228	GLILALIACKQNVSS
229	GLKEKIDAAKKCSET
230	GLRKVGDSVKAASKE
231	GLTGATKIRLERSAK
232	GLVAEAFGFKSDPKK
233	GMAKDGKFAVKDGEK
234	GNGFLDVFTSFGGLV
235	GNSYLFRNDKWTGWN
236	GPNLTEISKKITDSN
237	GQDEMKKDDQIAAAI
238	GSAVEITKLDEIKNA
239	GSEKLKAVAAAKGEN
240	GSGKAKEVLKGYVLE
241	GSGVLEGVKADKSKV
242	GSGVSENPFILEAKV
243	GSLLAGAYAISTLIK
244	GSSGEFSAMYDLMFE
245	GTAVEGAIKEVSELL
246	GTKLEGSAVEITKLD
247	GTLTAEKTTLVVKEG
248	GTRLEYTGIKSDGSG
249	GTSDKNNGSGVLEGV
250	GTSTLTITVNSKKTK
251	GTVTLSKNISKSGEV
252	GVKADKSKVKLTISD
253	GVLEGVKADKSKVKL
254	GVLEIAKKMREKLQR
255	GVNFDAFKDKKTGSG
256	GVSENPFILEAKVRA
257	GWIGDYADPLTFLSI
258	GWNTNFLERFDLCQL
259	GYTQFSSHNYSNPEY
260	GYVLEGTLTAEKTTL

261	HNGSLLAGAYAISTL
262	HTKNYCTLKKKENST
263	IAAAIALRGMAKDGK
264	IAAKAIGKKIHQNNG
265	IAKGIKEIVEAAGGS
266	IAKKMREKLQRVHTK
267	IALRGMAKDGKFAVK
268	IAPIYIYGNSYLFRN
269	IATVDKLELKGTSDK
270	IDAIKKDAALKGVNF
271	IDEIAAKAIGKKIHQ
272	IEKDFPIAPIYIYGN
273	IFTQGYTQFSSHNYS
274	IGDYADPLTFLSIFT
275	IGEVVADADAAKVAD
276	IGKKIHQNNGLDTEN
277	IGLILALIACKQNVS
278	IIEKDFPIAPIYIYG
279	IIIEKDFPIAPIYIY
280	IITRADGTRLEYTGI
281	IKCNNKTFNNLLKLT
282	IKEIVEAAGGSEKLK
283	IKEVSELLDKLVKAV
284	IKGAAESAVRKVLGA
285	IKKDAALKGVNFDAF
286	IKKSDLELDPIKRQD
287	IKQKLDGLKNEGLKE
288	IKRQDILRQAEEIII
289	ILALIACKQNVSSLD
290	ILEAKVRATTVAEKF
291	ILLTTFFVFINCKSQ
292	ILMTLFLFISCNNSG
293	ILRQAEEIIIEKDFP
294	ILSAIVTAADAAEQD
295	ILVNLLISCGLTGAT
296	IQEMTKTVSDAAEEN
297	IQLGNGFLDVFTSFG
298	IRLERSAKDITDEID
299	ISCGLTGATKIRLER
300	ISKKITDSNAVLLAV
301	ISSAILLTTFFVFIN
302	ISTLIKQKLDGLKNE
303	ITDEIDAIKKDAALK
304	ITDSNAVLLAVKEVE

305	ITGLIGDAVSSGLRK
306	ITRADGTRLEYTGIK
307	ITVNSKKTKDLVFTK
308	ITVQQYDSNGTKLEG
309	IYGNSYLFRNDKWTG
310	IYIYGNSYLFRNDKW
311	KAAGAVSAVSGEQIL
312	KAAKEMLANSVKELT
313	KADKSKVKLTISDDL
314	KAEGAIKGAAESAVR
315	KAIGKKIHQNNGLDT
316	KAKEVLKGYVLEGTL
317	KASVKGIAKGIKEIV
318	KAVAAAKGENNKGAG
319	KAVKTAEGASSGTAA
320	KCNNKTFNNLLKLTI
321	KCSETFTNKLKEKHT
322	KDAALKGVNFDAFKD
323	KDDPTNKFYQSVIQL
324	KDDQIAAAIALRGMA
325	KDFPIAPIYIYGNSY
326	KDGKYDLIATVDKLE
327	KDLVFTKENTITVQQ
328	KEAILKTNGTKTKGA
329	KEDGKTLVSKKVTSK
330	KEGTVTLSKNISKSG
331	KEGVTDADAKEAILK
332	KEIVEAAGGSEKLKA
333	KEKIDAAKKCSETFT
334	KEMLANSVKELTSPV
335	KEVEALLSSIDEIAA
336	KEVLKGYVLEGTLTA
337	KEVSELLDKLVKAVK
338	KFVIAIEEEATKLKE
339	KFYQSVIQLGNGFLD
340	KGAAESAVRKVLGAI
341	KGAEELGKLFESVEV
342	KGAGKLFGKAGAAAH
343	KGIAKGIKEIVEAAG
344	KGPNLTEISKKITDS
345	KGVNFDAFKDKKTGS
346	KGYVLEGTLTAEKTT
347	KIHQNNGLDTENNHN
348	KIITRADGTRLEYTG

349	KIRLERSAKDITDEI
350	KISSAILLTTFFVFI
351	KITDSNAVLLAVKEV
352	KKCSETFTNKLKEKH
353	KKDAALKGVNFDAFK
354	KKDDQIAAAIALRGM
355	KKIHQNNGLDTENNH
356	KKISSAILLTTFFVF
357	KKITDSNAVLLAVKE
358	KKMREKLQRVHTKNY
359	KKNTLSAILMTLFLF
360	KKSDLELDPIKRQDI
361	KKSDVKTYFTTVAAK
362	KKTAAWNSGTSTLTI
363	KKTKDLVFTKENTIT
364	KKVTSKDKSSTEEKF
365	KKYLLGIGLILALIA
366	KLDGLKNEGLKEKID
367	KLEGSAVEITKLDEI
368	KLFESVEVLSKAAKE
369	KLFGKAGAAAHGDSE
370	KLGIQEMTKTVSDAA
371	KLKAVAAAKGENNKG
372	KLQRVHTKNYCTLKK
373	KLTILVNLLISCGLT
374	KLTISDDLGQTTLEV
375	KLVKAVKTAEGASSG
376	KMREKLQRVHTKNYC
377	KNISKSGEVSVELND
378	KNKDGKYDLIATVDK
379	KNNGSGVLEGVKADK
380	KNSVSVDLPGEMKVL
381	KNTLSAILMTLFLFI
382	KNYCTLKKKENSTFT
383	KPLQKLGIQEMTKTV
384	KQKLDGLKNEGLKEK
385	KQNVSSLDEKNSVSV
386	KRQDILRQAEEIIIE
387	KSDLELDPIKRQDIL
388	KSDVKTYFTTVAAKL
389	KSKVKLTISDDLGQT
390	KTAAWNSGTSTLTIT
391	KTDLNSLPKEKSDIS
392	KTFNNLLKLTILVNL

393	KTGSGVSENPFILEA
394	KTKDLVFTKENTITV
395	KTKGAEELGKLFESV
396	KTKTDLNSLPKEKSD
397	KTLVSKKVTSKDKSS
398	KTTLVVKEGTVTLSK
399	KTYFTTVAAKLEKTK
400	KVADKASVKGIAKGI
401	KVKLTISDDLGQTTL
402	KVLGAITGLIGDAVS
403	KVLVSKEKNKDGKYD
404	KVRATTVAEKFVIAI
405	KWTGWNTNFLERFDL
406	KYDLIATVDKLELKG
407	KYLLGIGLILALIAC
408	LAGAYAISTLIKQKL
409	LALIACKQNVSSLDE
410	LANSVKELTSPVVAE
411	LAVKEVEALLSSIDE
412	LDEKNSVSVDLPGEM
413	LDKLVKAVKTAEGAS
414	LDPIKRQDILRQAEE
415	LDVFTSFGGLVAEAF
416	LEAKVRATTVAEKFV
417	LEGSAVEITKLDEIK
418	LEGTLTAEKTTLVVK
419	LEGVKADKSKVKLTI
420	LEIAKKMREKLQRVH
421	LELDPIKRQDILRQA
422	LEVFKEDGKTLVSKK
423	LEYTGIKSDGSGKAK
424	LFESVEVLSKAAKEM
425	LFGKAGAAAHGDSEA
426	LFLFISCNNSGKDGN
427	LGAITGLIGDAVSSG
428	LGIGLILALIACKQN
429	LGIQEMTKTVSDAAE
430	LGKLFESVEVLSKAA
431	LGNGFLDVFTSFGGL
432	LIACKQNVSSLDEKN
433	LIATVDKLELKGTSD
434	LIGDAVSSGLRKVGD
435	LIKKSDLELDPIKRQ
436	LIKQKLDGLKNEGLK

437	LILALIACKQNVSSL
438	LISCGLTGATKIRLE
439	LKAVAAAKGENNKGA
440	LKEKIDAAKKCSETF
441	LKGVNFDAFKDKKTG
442	LKGYVLEGTLTAEKT
443	LKLTILVNLLISCGL
444	LLAGAYAISTLIKQK
445	LLAVKEVEALLSSID
446	LLDKLVKAVKTAEGA
447	LLGIGLILALIACKQ
448	LLISCGLTGATKIRL
449	LLKLTILVNLLISCG
450	LLSSIDEIAAKAIGK
451	LLTTFFVFINCKSQV
452	LMFEVSKPLQKLGIQ
453	LMTLFLFISCNNSGK
454	LPGEMKVLVSKEKNK
455	LQKLGIQEMTKTVSD
456	LQRVHTKNYCTLKKK
457	LRGMAKDGKFAVKDG
458	LRQAEEIIIEKDFPI
459	LSAILMTLFLFISCN
460	LSAIVTAADAAEQDG
461	LSIFTQGYTQFSSHN
462	LSKAAKEMLANSVKE
463	LSKNISKSGEVSVEL
464	LSSIDEIAAKAIGKK
465	LTAEKTTLVVKEGTV
466	LTEISKKITDSNAVL
467	LTFLSIFTQGYTQFS
468	LTGATKIRLERSAKD
469	LTILVNLLISCGLTG
470	LTISDDLGQTTLEVF
471	LTITVNSKKTKDLVF
472	LTTFFVFINCKSQVA
473	LVAEAFGFKSDPKKS
474	LVFTKENTITVQQYD
475	LVKAVKTAEGASSGT
476	LVNLLISCGLTGATK
477	LVSKKVTSKDKSSTE
478	LVVKEGTVTLSKNIS
479	MAKDGKFAVKDGEKE
480	MFEVSKPLQKLGIQE

481	MIKCNNKTFNNLLKL
482	MKKDDQIAAAIALRG
483	MKKISSAILLTTFFV
484	MKKNTLSAILMTLFL
485	MKKYLLGIGLILALI
486	MKVLVSKEKNKDGKY
487	MLANSVKELTSPVVA
488	MREKLQRVHTKNYCT
489	MTLFLFISCNNSGKD
490	MYDLMFEVSKPLQKL
491	NAVLLAVKEVEALLS
492	NDKWTGWNTNFLERF
493	NELIKKSDLELDPIK
494	NFLERFDLCQLKLKN
495	NGFLDVFTSFGGLVA
496	NGSGVLEGVKADKSK
497	NGSLLAGAYAISTLI
498	NGTKLEGSAVEITKL
499	NHNGSLLAGAYAIST
500	NKDGKYDLIATVDKL
501	NKFYQSVIQLGNGFL
502	NKGAGKLFGKAGAAA
503	NKTFNNLLKLTILVN
504	NLLISCGLTGATKIR
505	NLLKLTILVNLLISC
506	NLTEISKKITDSNAV
507	NNGSGVLEGVKADKS
508	NNHNGSLLAGAYAIS
509	NNKGAGKLFGKAGAA
510	NNKTFNNLLKLTILV
511	NNLLKLTILVNLLIS
512	NPEYNELIKKSDLEL
513	NPFILEAKVRATTVA
514	NSGTSTLTITVNSKK
515	NSVKELTSPVVAESP
516	NSVSVDLPGEMKVLV
517	NSYLFRNDKWTGWNT
518	NTITVQQYDSNGTKL
519	NTLSAILMTLFLFIS
520	NTNFLERFDLCQLKL
521	NVSSLDEKNSVSVDL
522	NYCTLKKKENSTFTD
523	NYSNPEYNELIKKSD
524	PEYNELIKKSDLELD

525	PFILEAKVRATTVAE
526	PGEMKVLVSKEKNKD
527	PIAPIYIYGNSYLFR
528	PIKRQDILRQAEEII
529	PIYIYGNSYLFRNDK
530	PKKSDVKTYFTTVAA
531	PLQKLGIQEMTKTVS
532	PLTFLSIFTQGYTQF
533	PNLTEISKKITDSNA
534	PPTTAQGVLEIAKKM
535	PTNKFYQSVIQLGNG
536	PTTAQGVLEIAKKMR
537	QAEEIIIEKDFPIAP
538	QDEMKKDDQIAAAIA
539	QDILRQAEEIIIEKD
540	QGVLEIAKKMREKLQ
541	QGYTQFSSHNYSNPE
542	QIAAAIALRGMAKDG
543	QILSAIVTAADAAEQ
544	QKLDGLKNEGLKEKI
545	QKLGIQEMTKTVSDA
546	QLGNGFLDVFTSFGG
547	QNVSSLDEKNSVSVD
548	QRVHTKNYCTLKKKE
549	QSVIQLGNGFLDVFT
550	QTTLEVFKEDGKTLV
551	RAGWIGDYADPLTFL
552	RATTVAEKFVIAIEE
553	REKLQRVHTKNYCTL
554	RGMAKDGKFAVKDGE
555	RKVLGAITGLIGDAV
556	RLEYTGIKSDGSGKA
557	RNDKWTGWNTNFLER
558	RQAEEIIIEKDFPIA
559	RQDILRQAEEIIIEK
560	RVHTKNYCTLKKKEN
561	SAILLTTFFVFINCK
562	SAILMTLFLFISCNN
563	SAIVTAADAAEQDGK
564	SAMYDLMFEVSKPLQ
565	SAVEITKLDEIKNAL
566	SAVRKVLGAITGLIG
567	SAVSGEQILSAIVTA
568	SCGLTGATKIRLERS

569	SDKNNGSGVLEGVKA
570	SDLELDPIKRQDILR
571	SDPKKSDVKTYFTTV
572	SDVKTYFTTVAAKLE
573	SEAASKAAGAVSAVS
574	SEKIITRADGTRLEY
575	SEKLKAVAAAKGENN
576	SELLDKLVKAVKTAE
577	SENPFILEAKVRATT
578	SETFTNKLKEKHTDL
579	SFGGLVAEAFGFKSD
580	SGEFSAMYDLMFEVS
581	SGEQILSAIVTAADA
582	SGKAKEVLKGYVLEG
583	SGLRKVGDSVKAASK
584	SGTSTLTITVNSKKT
585	SGVLEGVKADKSKVK
586	SGVSENPFILEAKVR
587	SIDEIAAKAIGKKIH
588	SIFTQGYTQFSSHNY
589	SKAAGAVSAVSGEQI
590	SKAAKEMLANSVKEL
591	SKKITDSNAVLLAVK
592	SKKTKDLVFTKENTI
593	SKKVTSKDKSSTEEK
594	SKNISKSGEVSVELN
595	SKPLQKLGIQEMTKT
596	SKVKLTISDDLGQTT
597	SLLAGAYAISTLIKQ
598	SNAVLLAVKEVEALL
599	SNGTKLEGSAVEITK
600	SNPEYNELIKKSDLE
601	SSAILLTTFFVFINC
602	SSGEFSAMYDLMFEV
603	SSIDEIAAKAIGKKI
604	STGSVGTAVEGAIKE
605	STLIKQKLDGLKNEG
606	STLTITVNSKKTKDL
607	SVEVLSKAAKEMLAN
608	SVIQLGNGFLDVFTS
609	SVKELTSPVVAESPK
610	SVKGIAKGIKEIVEA
611	SVKGPNLTEISKKIT
612	SVSVDLPGEMKVLVS

613	SYLFRNDKWTGWNTN
614	TAAIGEVVADADAAK
615	TAAWNSGTSTLTITV
616	TAEKTTLVVKEGTVT
617	TAQGVLEIAKKMREK
618	TAVEGAIKEVSELLD
619	TDEIDAIKKDAALKG
620	TDLNSLPKEKSDISS
621	TDSNAVLLAVKEVEA
622	TEISKKITDSNAVLL
623	TENNHNGSLLAGAYA
624	TFFVFINCKSQVADK
625	TFLSIFTQGYTQFSS
626	TFNNLLKLTILVNLL
627	TFTNKLKEKHTDLGK
628	TGATKIRLERSAKDI
629	TGIKSDGSGKAKEVL
630	TGLIGDAVSSGLRKV
631	TGSGVSENPFILEAK
632	TGSSGEFSAMYDLMF
633	TGWNTNFLERFDLCQ
634	TILVNLLISCGLTGA
635	TITVNSKKTKDLVFT
636	TITVQQYDSNGTKLE
637	TKDLVFTKENTITVQ
638	TKGAEELGKLFESVE
639	TKIRLERSAKDITDE
640	TKKTAAWNSGTSTLT
641	TKLEGSAVEITKLDE
642	TKNYCTLKKKENSTF
643	TKTDLNSLPKEKSDI
644	TLEVFKEDGKTLVSK
645	TLFLFISCNNSGKDG
646	TLIKQKLDGLKNEGL
647	TLSAILMTLFLFISC
648	TLSKNISKSGEVSVE
649	TLTAEKTTLVVKEGT
650	TLTITVNSKKTKDLV
651	TLVSKKVTSKDKSST
652	TLVVKEGTVTLSKNI
653	TNFLERFDLCQLKLK
654	TNKFYQSVIQLGNGF
655	TQGYTQFSSHNYSNP
656	TRLEYTGIKSDGSGK

657	TSDKNNGSGVLEGVK
658	TSFGGLVAEAFGFKS
659	TSTLTITVNSKKTKD
660	TTAQGVLEIAKKMRE
661	TTFFVFINCKSQVAD
662	TTLEVFKEDGKTLVS
663	TTLVVKEGTVTLSKN
664	TTVAEKFVIAIEEEA
665	TVAEKFVIAIEEEAT
666	TVQQYDSNGTKLEGS
667	TVTLSKNISKSGEVS
668	TYFTTVAAKLEKTKT
669	VAAKLEKTKTDLNSL
670	VADKASVKGIAKGIK
671	VAEKFVIAIEEEATK
672	VDLPGEMKVLVSKEK
673	VEAAGGSEKLKAVAA
674	VEALLSSIDEIAAKA
675	VEGAIKEVSELLDKL
676	VEVLSKAAKEMLANS
677	VFINCKSQVADKDDP
678	VFKEDGKTLVSKKVT
679	VFTKENTITVQQYDS
680	VFTSFGGLVAEAFGF
681	VGTAVEGAIKEVSEL
682	VHTKNYCTLKKKENS
683	VIQLGNGFLDVFTSF
684	VKAVKTAEGASSGTA
685	VKEGTVTLSKNISKS
686	VKELTSPVVAESPKK
687	VKEVEALLSSIDEIA
688	VKGIAKGIKEIVEAA
689	VKGPNLTEISKKITD
690	VKLTISDDLGQTTLE
691	VKTYFTTVAAKLEKT
692	VLEGTLTAEKTTLVV
693	VLEGVKADKSKVKLT
694	VLEIAKKMREKLQRV
695	VLGAITGLIGDAVSS
696	VLKGYVLEGTLTAEK
697	VLLAVKEVEALLSSI
698	VLSKAAKEMLANSVK
699	VNFDAFKDKKTGSGV
700	VNLLISCGLTGATKI

701	VRATTVAEKFVIAIE
702	VRKVLGAITGLIGDA
703	VSAVSGEQILSAIVT
704	VSEKIITRADGTRLE
705	VSELLDKLVKAVKTA
706	VSENPFILEAKVRAT
707	VSGEQILSAIVTAAD
708	VSKKVTSKDKSSTEE
709	VSKPLQKLGIQEMTK
710	VTLSKNISKSGEVSV
711	VVADADAAKVADKAS
712	VVKEGTVTLSKNISK
713	WIGDYADPLTFLSIF
714	WNTNFLERFDLCQLK
715	WTGWNTNFLERFDLC
716	YADPLTFLSIFTQGY
717	YAISTLIKQKLDGLK
718	YCTLKKKENSTFTDE
719	YDLIATVDKLELKGT
720	YDLMFEVSKPLQKLG
721	YFTTVAAKLEKTKTD
722	YGNSYLFRNDKWTGW
723	YIYGNSYLFRNDKWT
724	YLFRNDKWTGWNTNF
725	YLLGIGLILALIACK
726	YNELIKKSDLELDPI
727	YQSVIQLGNGFLDVF
728	YSNPEYNELIKKSDL
729	YTGIKSDGSGKAKEV
730	YTQFSSHNYSNPEYN
731	YVLEGTLTAEKTTLV
